# Catalyst-free four-component domino synthetic approach toward versatile multicyclic spirooxindole pyran scaffolds†

**DOI:** 10.1039/c9ra03214b

**Published:** 2019-05-28

**Authors:** Aref Mohammadi, Mohammad Bayat, Shima Nasri

**Affiliations:** Department of Chemistry, Faculty of Science, Imam Khomeini International University Qazvin Iran m.bayat@sci.ikiu.ac.ir bayat_mo@yahoo.com +98 28 33780040

## Abstract

A new versatile strategy involving a sequential four-component reaction of the nitroketene dithioacetals, alkylamine/benzylamine, isatin and various enolizable active methylene structures (pyrazolone, barbituric acid, 1,3-indandione and 2-hydroxy-1,4-naphthoquinone) as precursors under mild and catalyst-free conditions results in the synthesis of new functionalized spirooxindole pyrans named spiro[indoline-3,4′-pyrano[2,3-*c*]pyrazol], spiro[indoline-3,5′-pyrano[2,3-*d*]pyrimidine], spiro[indeno[1,2-*b*]pyran-4,3′-indoline] and spiro[benzo[*g*]chromene-4,3′-indoline] in moderate to good yields. The use of various active methylene compounds affords a range of skeletally distinct spirooxindole-based heterocycles with potential biological properties. The present strategy has many advantages, such as convenient one-pot operation, simple workup procedures and straightforward isolation without using tedious purification steps such as column chromatography, progress under catalyst-free condition and high molecular diversity.

## Abstract 2

Synthesis of spirooxindole pyrans by a sequential four-component reaction of nitroketene dithioacetals, alkylamine/benzylamine, isatin, and active methylene compounds.

## Graphical abstract

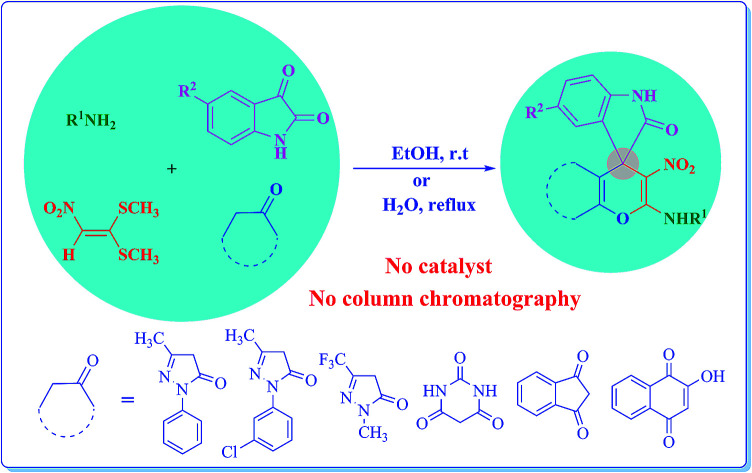

## Introduction

A significant challenge to explore biological targets is the design of an economical and highly efficient chemical reaction to supply structurally diverse compounds with excellent biological properties.^1^ Therefore, to satisfy the need for small molecules containing bioactive heterocycles as biological targets, access to a wide variety of compounds with potential biological activities is highly desirable.^2,3^ The 4*H*-pyran derivatives are defined as useful biological and pharmacological targets with biological properties such as antiviral,^4^ antibacterial,^5^ antitumor,^6^ and diuretic activities.^7^ In addition, isatin and its derivatives have attractive biological properties and are widely used in the synthesis of spirooxindole-based organic compounds.^8^ Spirooxindole system is one of the most phenomenal spirocycles and the central structural framework found in a diversity of natural products and bioactive molecules.^9^ The existence of a chiral spiro center around a biologically active scaffold leads to structural rigidity and complexity, which is an ongoing challenge for synthetic organic chemists.^10^ Hence, heterocyclic spirooxindole pyrans are attractive synthetic targets for synthetic chemists due to their biological activities such as antiviral, antibacterial, antifungal, anti-HIV, and anti-cancer activities.^11^ Various groups have developed synthetic approaches to access this scaffold.^3,11^ In spite of the development of versatile reactions to synthesize these structures, new, simple and highly efficient methods are still required.

In organic synthesis, development of synthetically useful and unique organic reaction methods in eco-friendly media that generate complex molecular libraries with a minimum number of synthetic steps is a major challenge that can be resolved by multicomponent reactions (MCRs) as the most efficient strategy in modern organic synthesis.^12^ The inherent advantages of MCRs including operational simplicity, step efficiency, lower energy consumption and costs, and high atom economy without waste production have resulted in designing novel MCRs using eco-friendly solvents as one of the goals of sustainable chemistry and synthetic chemistry.^13^

There are many reports on multicomponent entries in the synthesis of spirooxindole-4*H*-pyrans from readily available starting materials including isatin, malononitrile, and 1,3-dicarbonyl compounds. This class of reaction has been carried out under different conditions as follows: in the presence of *p*-toluenesulfonic acid (*p*-TSA) in aqueous media,^14^ [BMIm]BF_4_ as an ionic liquid catalyst at ambient temperature,^15^ piperidine under ultrasound irradiation,^16^ proline-derived thiourea catalyst,^3^ CuFe_2_O_4_ (10 mol%) as an inexpensive, magnetically recoverable, and reusable catalyst in refluxing water,^17^ SBA-Pr-NH_2_ as an efficient heterogeneous nanoporous solid basic catalyst in an aqueous medium,^18^ α-amylase extracted from hog pancreas,^19^ silica-bonded *N*-propyltriethylenetetramine as a heterogeneous solid base catalyst,^20^ triethylamine in ethanol,^21^ silica-bonded ionic liquids as a recyclable catalyst,^22^ 1 mol% of (DHQD)_2_PYR,^23^ ytterbium triflate as a catalyst at room temperature,^24^ under visible-light irradiation in water-ethyl lactate at room temperature,^25^ in the presence of a catalytic amount of 1,4-diazabicyclo[2.2.2]octane (DABCO) in ethanol or H_2_O/EtOH mixture under reflux conditions,^26^ in dimethylsulfoxide (DMSO) as a highly polar aprotic solvent,^11^ by dabco-based ionic liquids,^27^ using guanidine-functionalized magnetic Fe_3_O_4_ nanoparticles (MNPs) as an efficient heterogeneous catalyst.^28^

Although various catalysts have been applied for the synthesis of these compounds, the role of catalysts is the same in these reactions and it was to catalyze the Knoevenagel condensation, Michael addition, and cyclization reactions. Each of the procedures has its own benefits with at least one drawback, such as low yield, long reaction time, harsh reaction conditions, and use of toxic and expensive catalysts or solvents; hence, there is still a requirement for simple, efficient and economical methods.

**Scheme 1:**
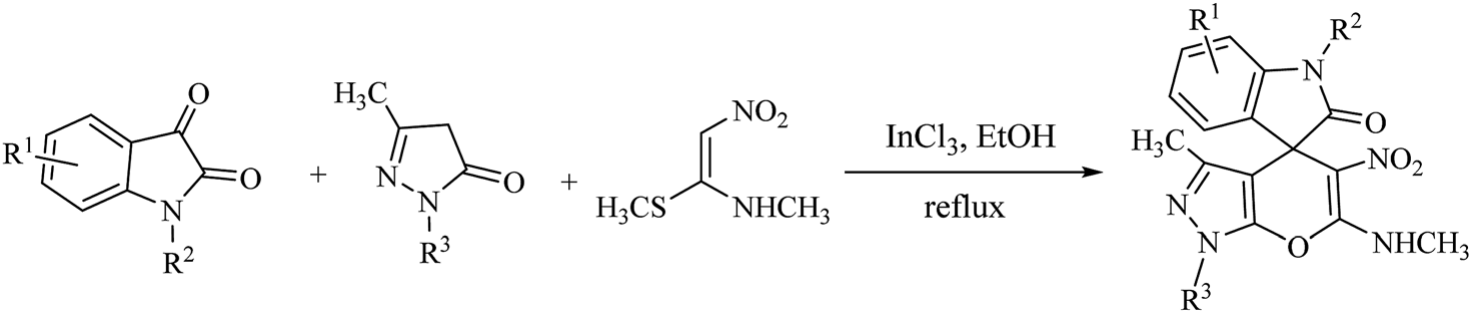
Synthesis of spiro(indoline-3,4′-pyrano[2,3-*c*]pyrazol)-2-one derivatives catalyzed by indium trichloride.

In recent years, ketene aminals (KAs) have been used as powerful and versatile enamine analogues with unique structural properties in the synthesis of various types of fused five- and six-membered heterocycles and drug-like compounds.^29^ In this way, specific strategies have been developed for the synthesis of substituted spirooxindole-4*H*-pyrans based on utilizing KAs as starting materials as follows. In 2014, Poomathi *et al.* reported a versatile high-yielding indium trichloride-mediated one-pot regioselective reaction for the synthesis of spiroxindoles *via* domino one-pot, three-component reaction of isatins, pyrazoles, and (*E*-)-*N*-methyl-1-(methylthio)-2-nitroethenamine (Scheme 1).^30^

**Scheme 2:**
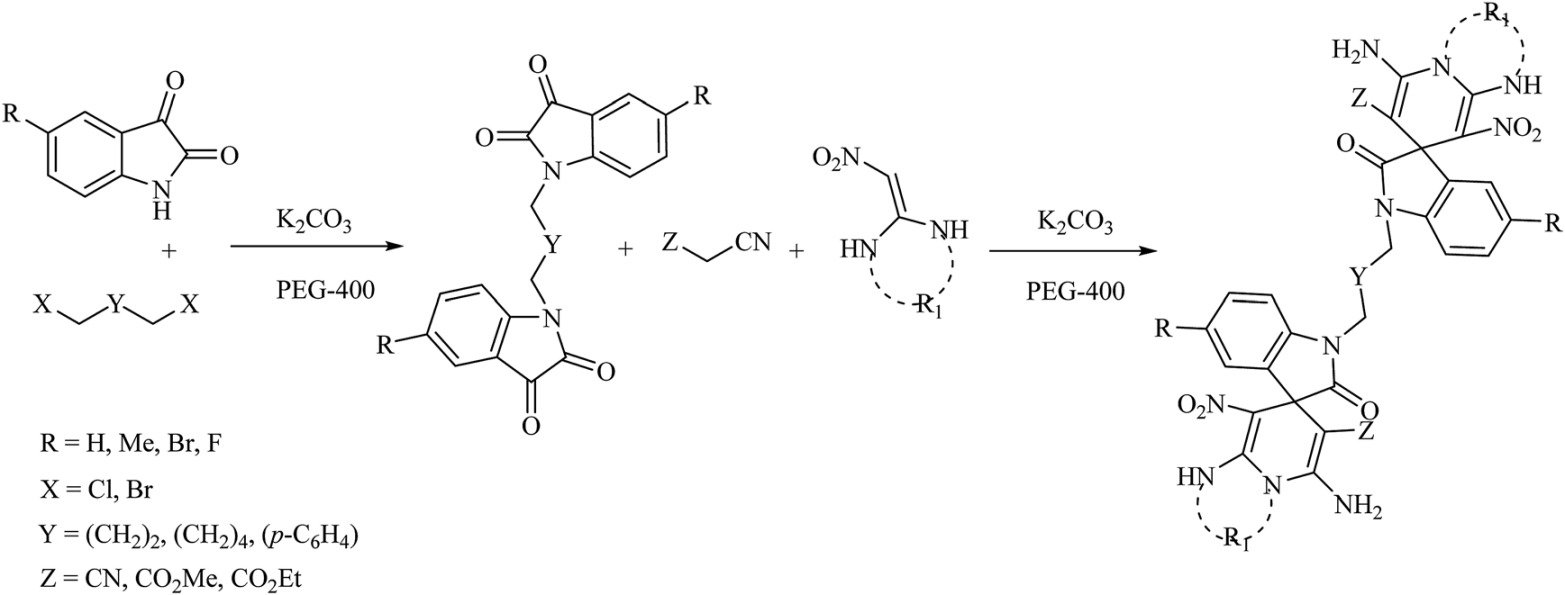
The one-pot, multi-component synthesis of bis-spirooxindoles in PEG-400/K_2_CO_3_ at room temperature.

In 2017, Safari *et al.* reported the synthesis of novel symmetrical bis-spirooxindole derivatives from the reaction of isatins, dihalides, malono derivatives and C–H activated carbonyl compounds or ketene aminal derivatives in the presence of potassium carbonate (K_2_CO_3_) in polyethylene glycol 400 (PEG-400) at room temperature (Scheme 2).^31^

With this article background and owing to our interest in catalyst-free approaches by using environmentally friendly solvents as a green and safe method for the formation of molecular architectures with a wide range of potential biological activities,^32–35^ herein, we designed and introduced an improved, catalyst-free, and easy method to access the spirooxindoles and 4*H*-pyrans, for which the results are shown in the Results and discussion section.

## Results and discussion

**Scheme 3:**
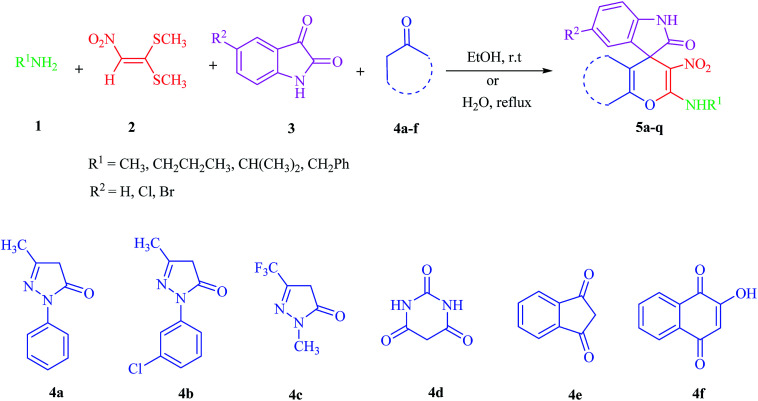
Synthetic approaches for the formation of spiro[indoline-3,4′-pyrano[2,3-*c*]pyrazol], spiro[indoline-3,5′-pyrano[2,3-*d*]pyrimidine], spiro[indeno[1,2-*b*]pyran-4,3′-indoline] and spiro[benzo[*g*]chromene-4,3′-indoline] (5a–q).

Due to the pharmacological activities of spirooxindole motifs and the bioactivity of pharmacophores such as naphthoquinone, indandion, pyrazolone, and pyrimidine derivatives, we synthesized products 5 which comprise both of spirooxindole and one of these pharmacophores. The hybrid molecules may inherit biological properties of both spirooxindole and pharmacophore structures. Synthesis of 5 could be easily achieved by tandem Knoevenagel condensation/Michael addition/imine–enamine tautomerism/*O*-cyclization of the reaction of amines 1, 1,1-bis(methylthio)-2-nitroethene 2, isatin 3 and enolizable active methylene structures 4a–f (pyrazolone, barbituric acid, 1,3-indandione and 2-hydroxy-1,4-naphthoquinone) (Scheme 3).

In this paper, KAs were derived from the addition of various alkylamine/benzylamine 1 to 1,1-bis(methylthio)-2-nitroethene 2, wherein the electron density of the α-carbon (C1) increased due to the conjugation effect of both the electron-donating nitrogen and sulfur atoms.^29^ Isatin, as an important distinguished building block, is a type of unsymmetrical cyclic ketone with excellent reactivity that has been used in the synthesis of different types of spirooxaindole skeletons.^36^ In this study, a variety of multicyclic spirooxindole pyran scaffolds (5a–q) were synthesized; these derivatives provide a class of structurally diverse compounds that demonstrate promise for future bioassays and medical therapy applications.

**Scheme 4:**
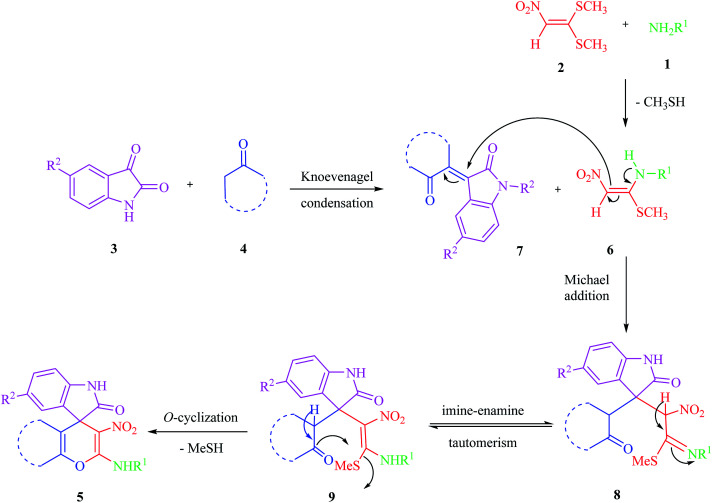
A plausible mechanism for the formation of 5 in catalyst-free conditions.

The acceptable reaction mechanism is designated in Scheme 4. In the case of the synthesis of 5, it is possible that initially, the formation of ketene aminal (KA) 6 occured through the addition of amine 1 to 1,1-bis (methylthio)-2-nitroethene 2. Then, Michael acceptor 7 formed through Knoevenagel condensation between isatin 3 and enolizable active methylene structures 4, which was followed by the loss of water molecules. Then the KA 6 as an enamine added to the Knoevenagel adduct 7 in a Michael addition type reaction to give open chain intermediate 8, which after successive imine–enamine tautomerization underwent *O*-cyclization *via* an attack of the enol form of C–H-activated compound on KA, leading to product 5 (Scheme 4).

We surveyed the scope of these reactions by varying the derivatives of amine 1, isatin 3, and the active methylene compounds 4 to produce product 5 (Table 1). The reaction proceeds cleanly and completely in the presence of different reagents to afford a series of spiro products 5a–q in 58–86% yields. It is noteworthy that the expected product 5 was obtained in good yield when pyrazolone was used, but using other C–H activated compounds, the expected product 5 was acquired in moderate yields. The substituents on the aromatic ring of the isatin like chlorine or bromine affected the reaction and gave desired products in a long time with fewer yields. The products 5d–5q are novel compounds (Table 1) that have not been reported in the previous literature.

**Table 1:** Substrate scope study of multicyclic spirooxindole pyran scaffolds with a series of amines, isatin derivatives, and active methylene structures

Entry	Active methylene structures	R^1^	R^2^	Product	Time^a^ (h)	Yield (%)
1	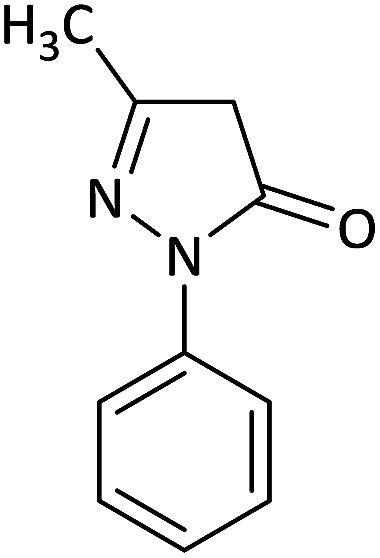	CH_3_	H	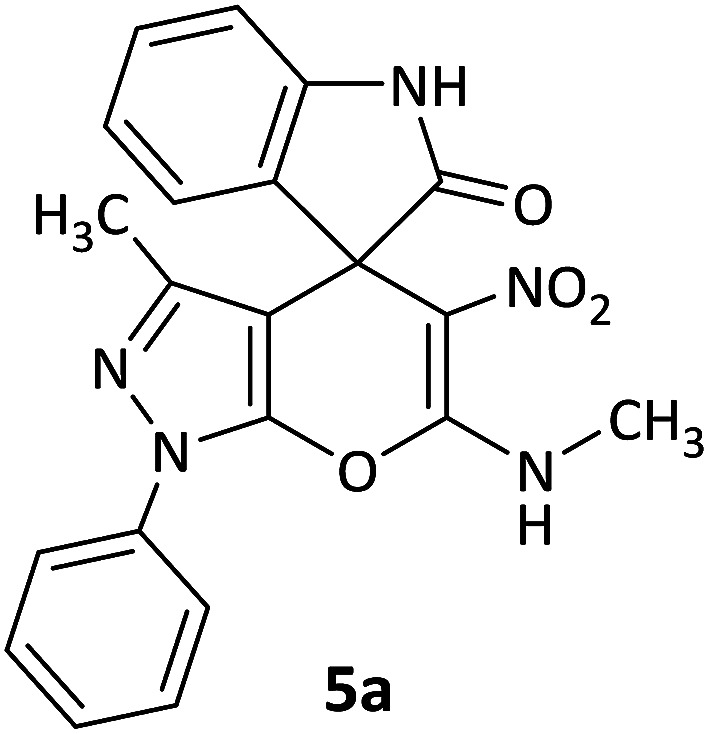	8	85
2	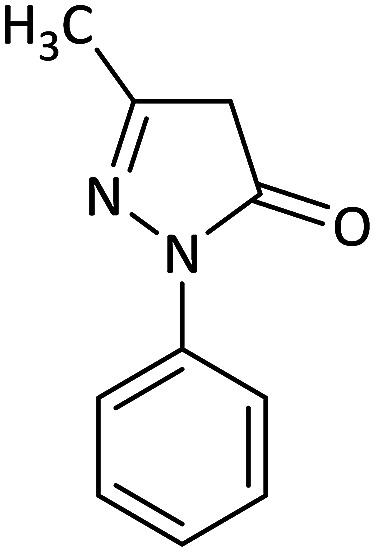	CH_3_	Cl	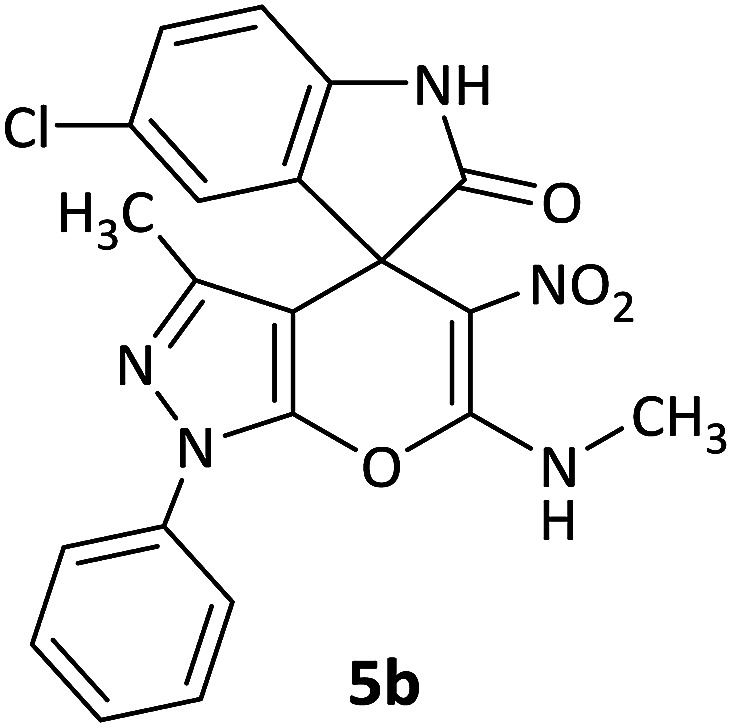	8	80
3	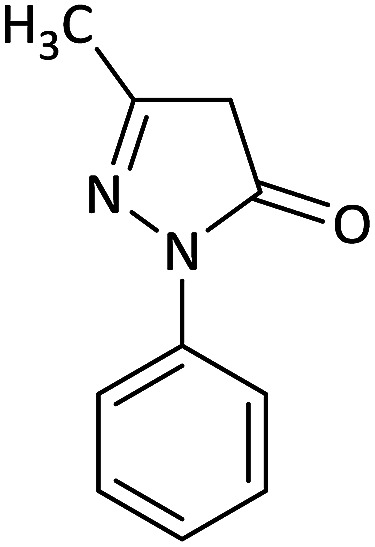	CH_3_	Br	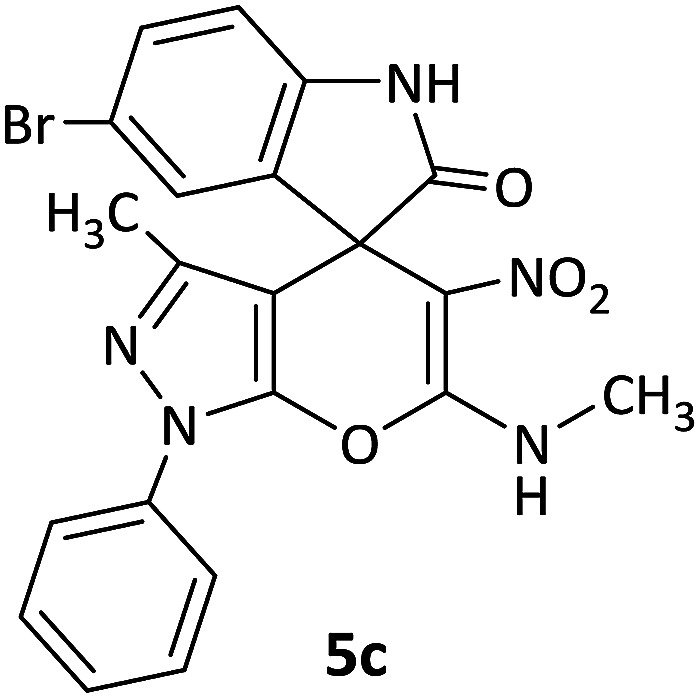	9	78
4	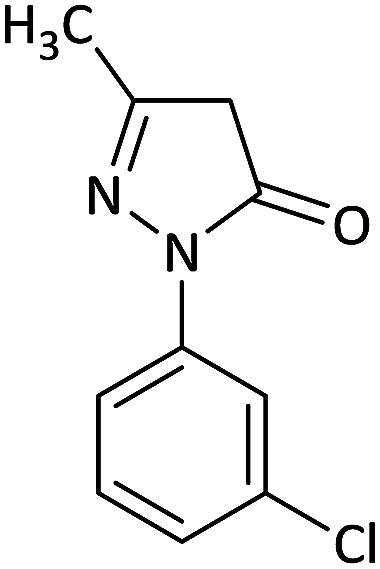	CH_3_	H	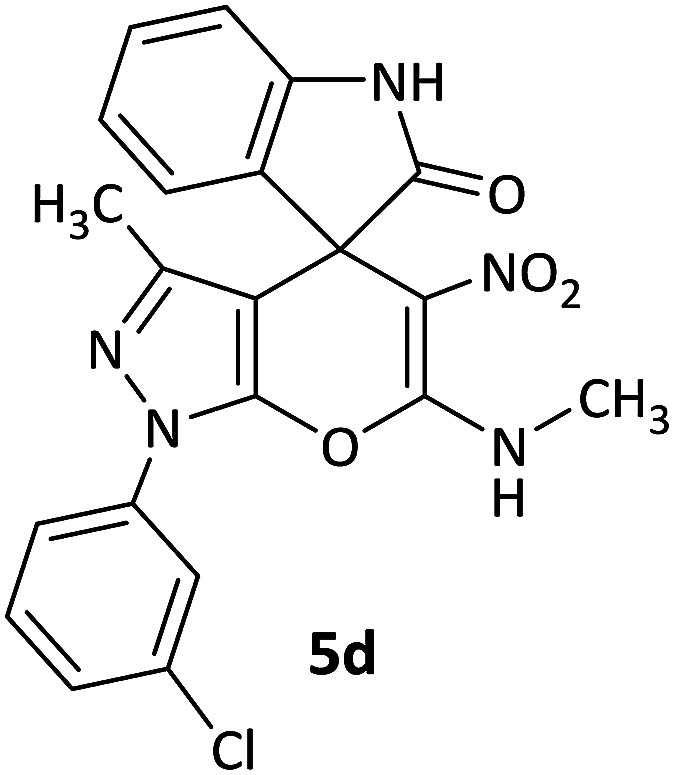	10	86
5	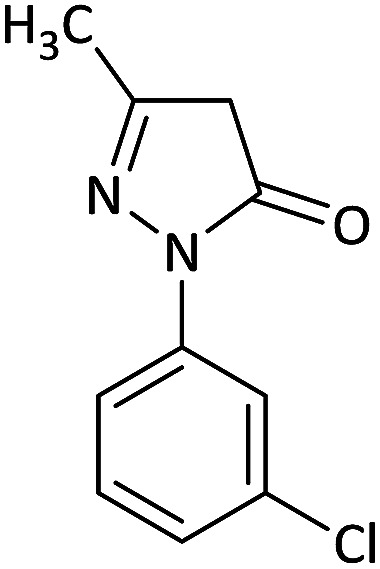	CH_3_	Br	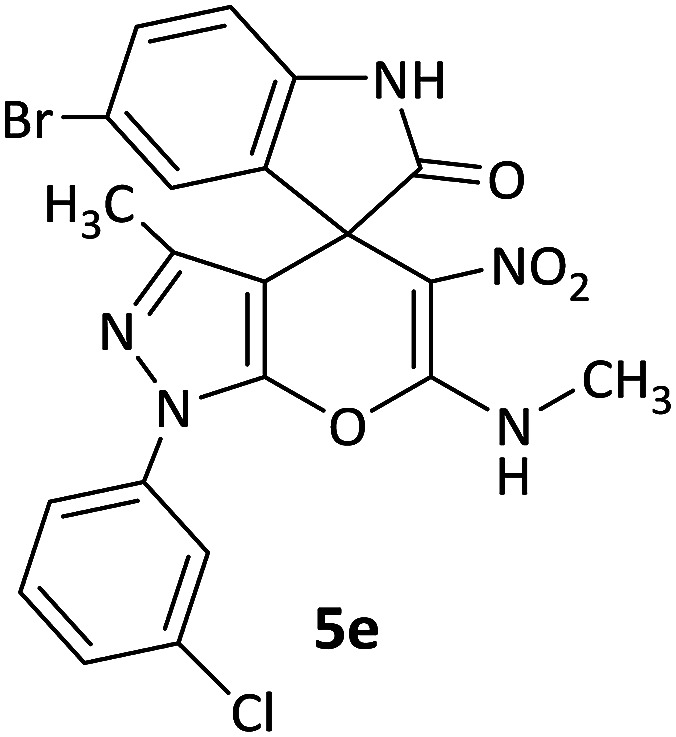	12	75
6	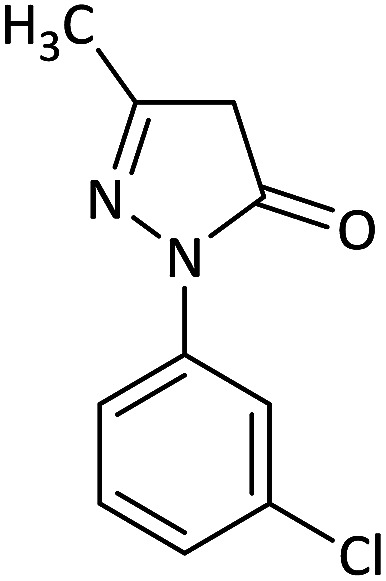	CH_3_	Cl	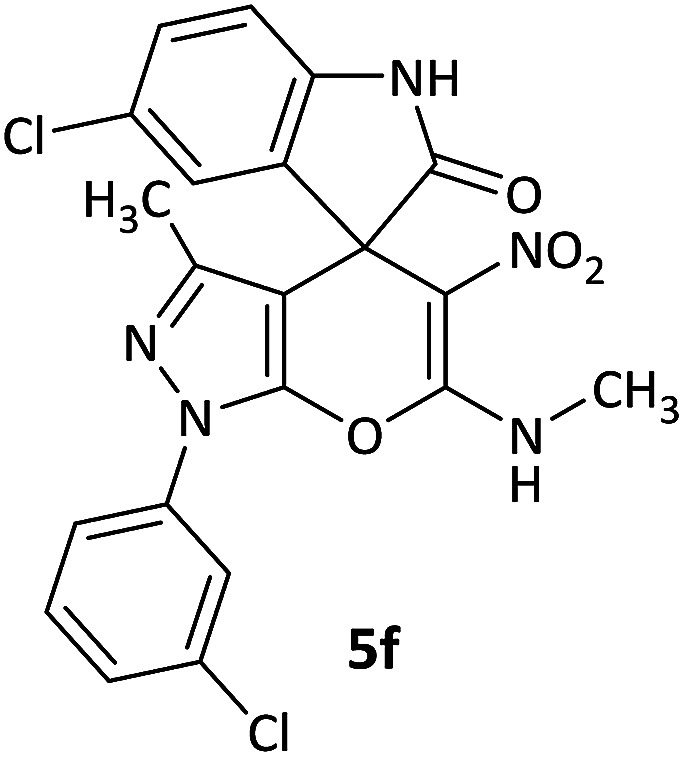	10	80
7	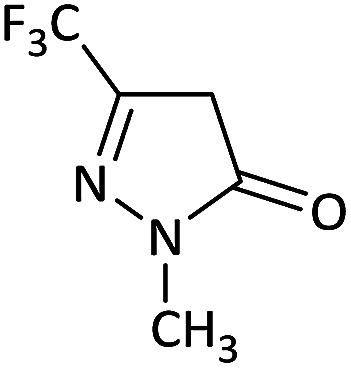	CH_3_	Br	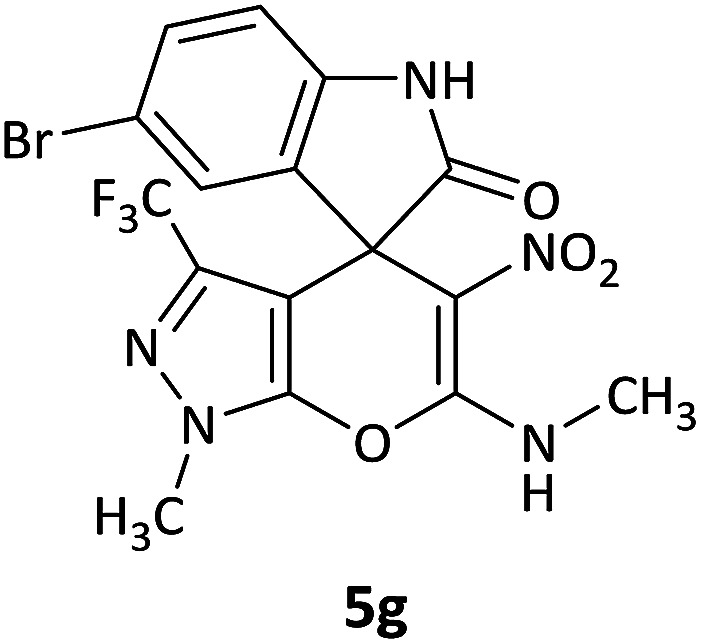	10	62
8	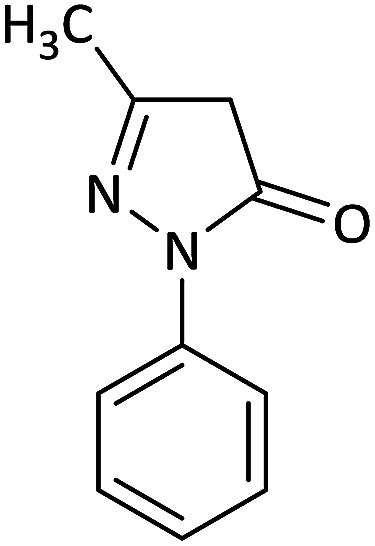	CH_2_Ph	Cl	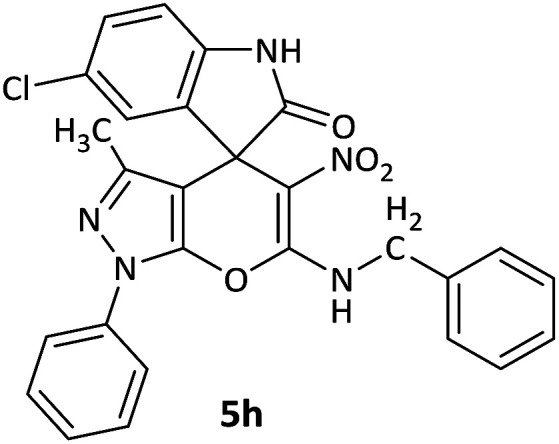	12	58
9	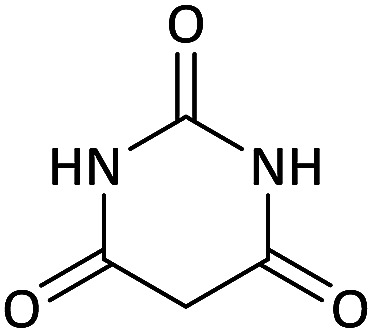	CH_3_	Cl	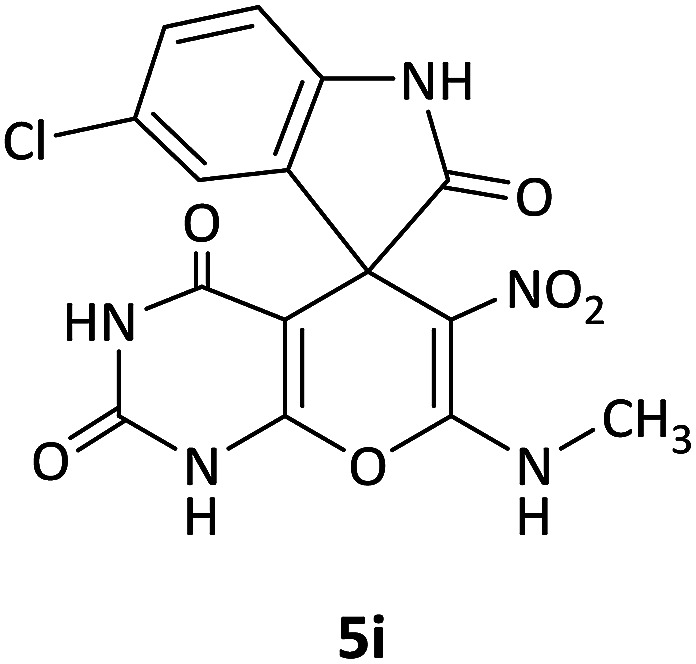	10	65
10	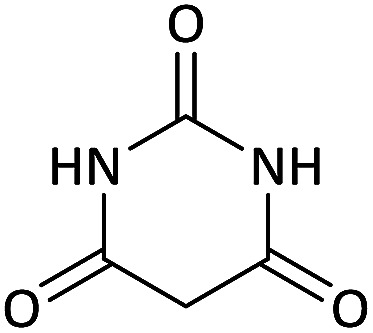	CH_2_CH_2_CH_3_	Cl	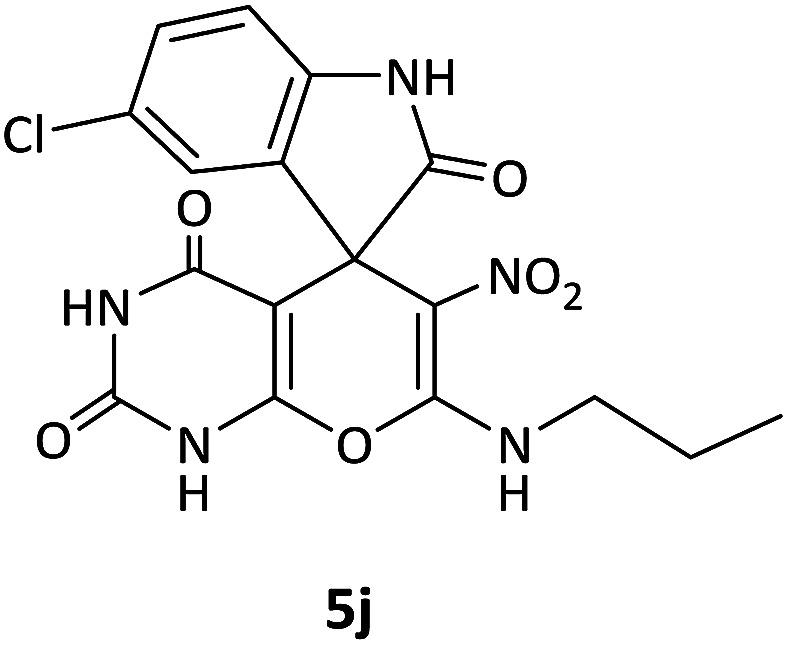	10	75
11	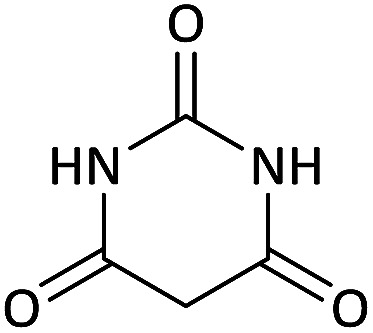	CH(CH_3_)_2_	Cl	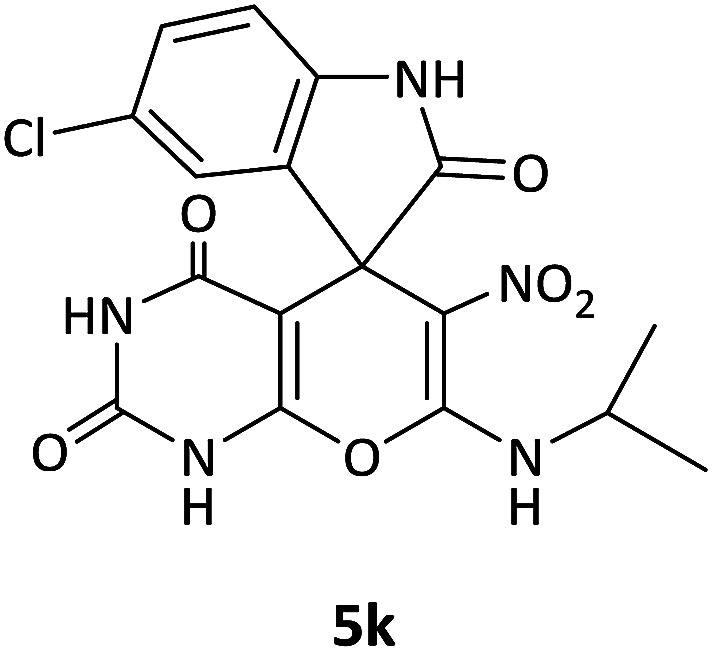	10	76
12	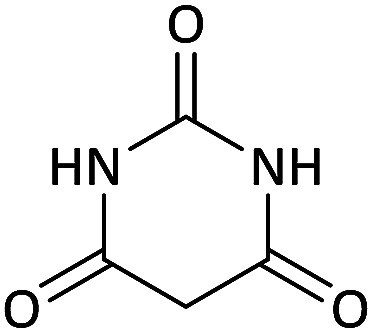	CH_2_CH_2_CH_3_	Br	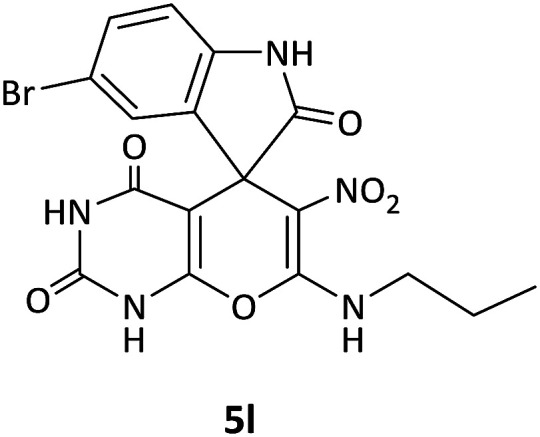	12	71
13	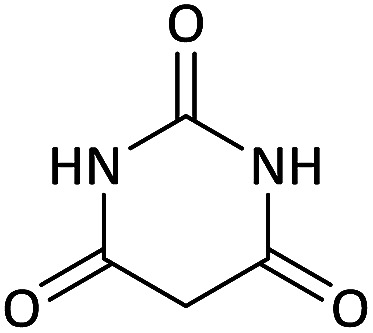	CH(CH_3_)_2_	Br	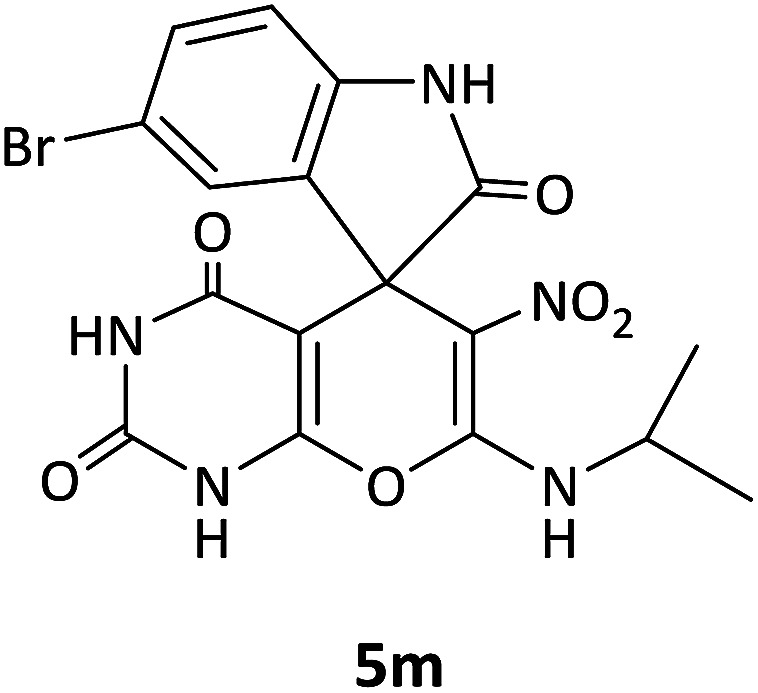	12	72
14	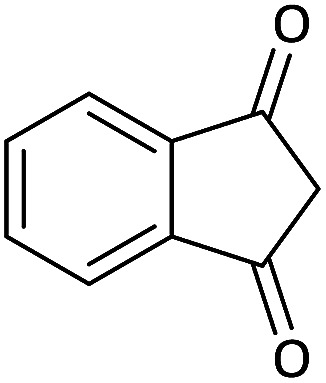	CH_3_	Cl	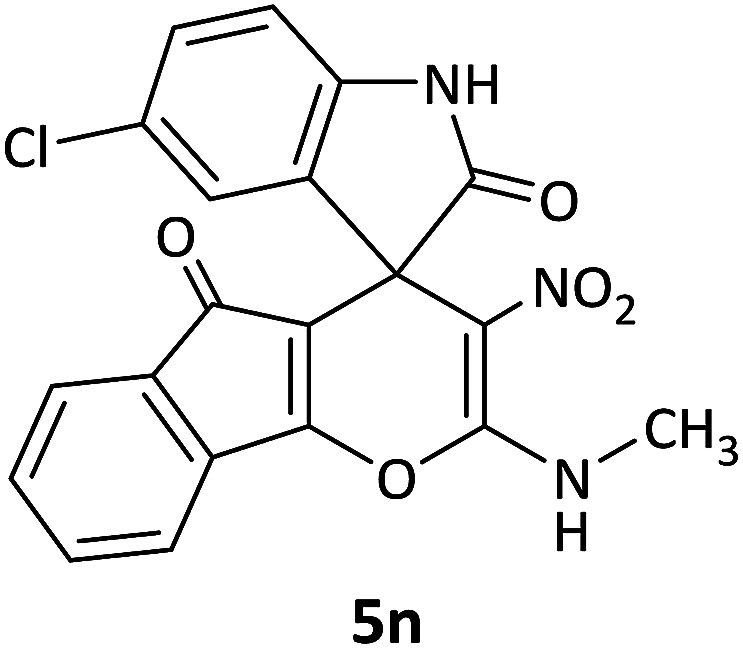	12	65
15	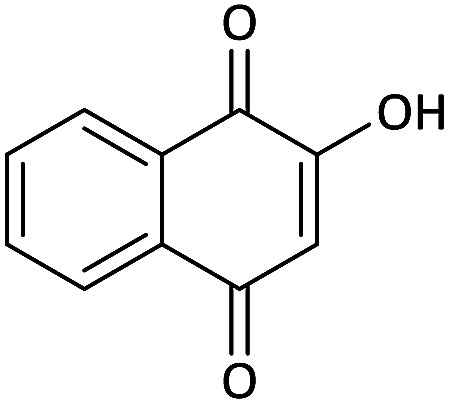	CH_2_Ph	H	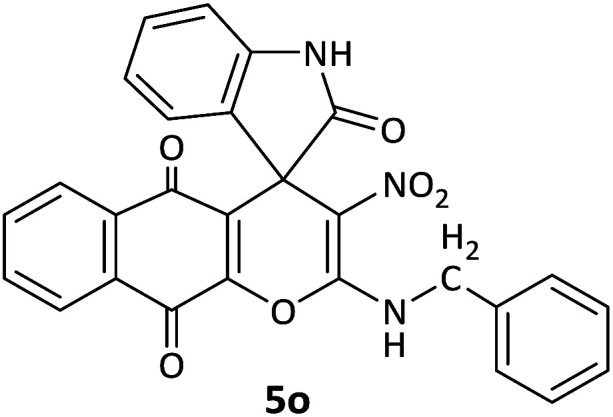	12	65
16	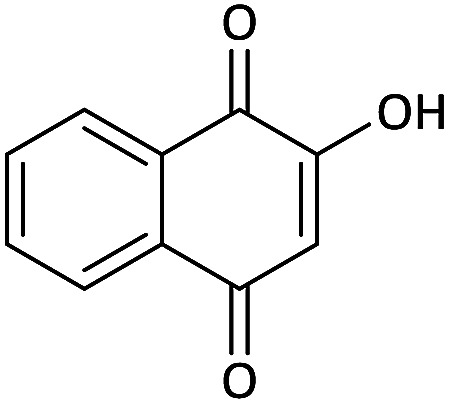	CH_2_CH_2_CH_3_	Cl	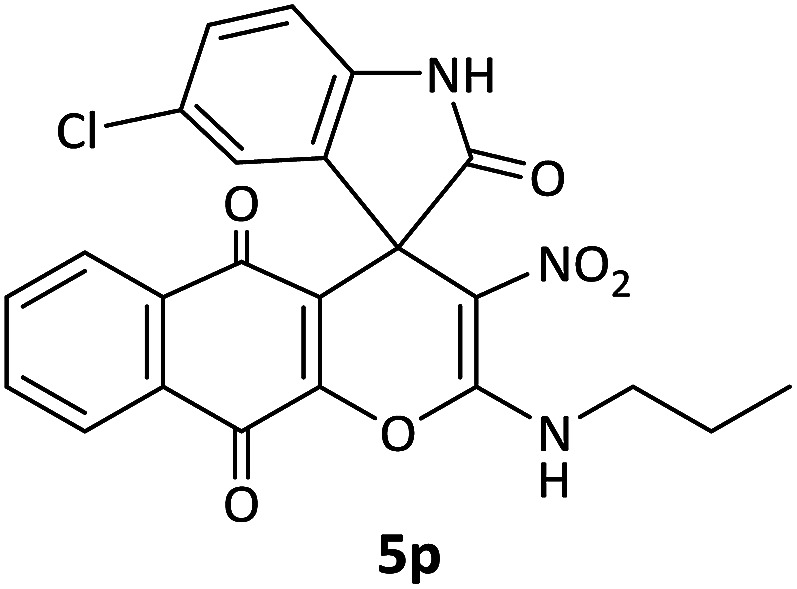	8	62
17	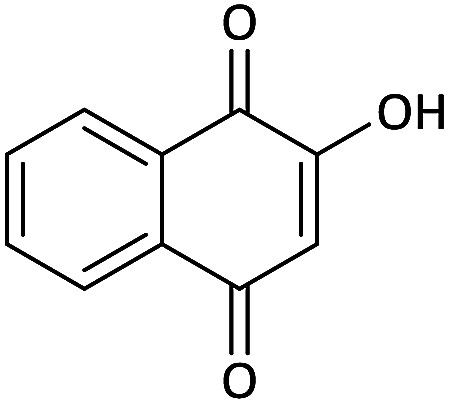	CH_2_CH_2_CH_3_	Br	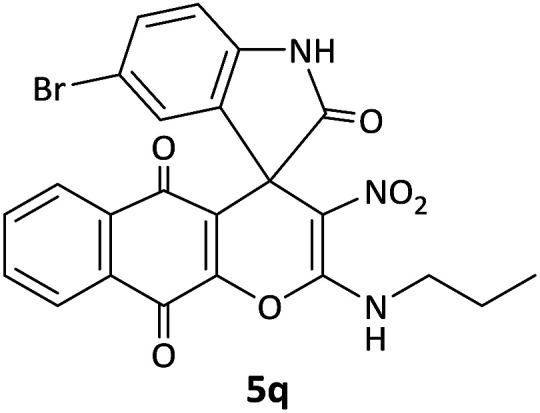	8	60

a The reaction time of the second step.

The structures of synthesized derivatives were concluded by elemental and spectral analysis such as FT-IR, ^1^H, ^13^C NMR and mass spectroscopy. The mass spectrum of compound 5a exhibited a molecular ion peak at *m*/*z* 403, which was in accordance with the offered structure. The ^1^H NMR spectrum of 5a displayed one singlet for CH_3_ group (*δ* 1.58 ppm), one doublet for 3H of CH_3_ group attached to NH (*δ* 3.17 ppm, ^3^*J*_HH_ = 4.8 Hz), aromatic region of the spectrum for the aromatic moieties (*δ* 6.87–7.73 ppm), one multiplet for NH attached to CH_3_ (*δ* 10.71–10.73 ppm) and one singlet for NH group of isatin (*δ* 10.74 ppm). The ^1^H-decoupled ^13^C NMR spectrum of 5a exhibited 21 distinguished resonances. One peak appeared at *δ* 176.6 ppm, which was attributed to one amide carbonyl group and the specific peaks of *C*_spiro_, NHCH_3_ and CH_3_, were assigned at *δ* 50.0, 29.4 and 12.2 ppm, which confirmed the selective synthesis of 5a.

## Conclusions

In General, we developed an environment friendly process for the synthesis of a library of spiro[indoline-3,4′-pyrano[2,3-*c*]pyrazol], spiro[indoline-3,5′-pyrano[2,3-*d*]pyrimidine], spiro[indeno[1,2-*b*]pyran-4,3′-indoline] and spiro[benzo[*g*]chromene-4,3′-indoline] compounds in moderate to good yields (58–86%). This reaction proceeded *via* sequential four-component reaction between alkylamine/benzylamine, 1,1-bis(methylthio)-2-nitro ethylene, isatin and various active methylene compounds in EtOH or H_2_O medium. Prominent advantages of this method are the diversity of molecular structure, an easy workup, the absence of a catalyst and operational simplicity.

## Experimental section

### General

The nitroketene dithioacetals, isatin derivatives, various amines, various active methylene compounds and solvents were obtained from Sigma Aldrich and Fluka Co., which were used without further purification. IR spectra: Bruker Tensor 27 spectrometer. NMR spectra: Bruker DRX-300 Avance instrument (300 MHz for ^1^H and 75.4 MHz for ^13^C) with DMSO-*d*_6_ as solvents. Chemical shifts are expressed in parts per million (ppm), and coupling constant (*J*) are reported in hertz (Hz). Mass spectra: Agilent 5975C VL MSD with Triple-Axis detector operating at an ionization potential of 70 eV. Elemental analyses for C, H and N: Heraeus CHNO-Rapid analyzer. Melting points: electrothermal 9100 apparatus.

### General procedure for the synthesis of 5a–h and 5n–q

A mixture of amine (1 mmol), 1,1-bis(methylthio)-2-nitro ethylene (0.165 g, 1 mmol), and 10 mL EtOH was taken in a 50 mL round-bottomed flask, and was stirred for 4 h. Then, isatin derivatives (1 mmol) and active methylene compounds (1 mmol) were added to the reaction mixture, and was stirred for a period of time shown in Table 1, which was monitored by TLC (ethyl acetate/*n*-hexane, 6 : 4). The reaction mixture was cooled to room temperature and the precipitate was filtered to obtain the crude product. The solid was washed with 96% ethanol and dried in an oven at 150 °C to yield product 5 and analyzed by ^1^H NMR and ^13^C NMR spectroscopy. Only in the case of 5n, the round-bottom flask was fitted with a reflux condenser and heated with stirring in an oil-bath at reflux temperature.

### General procedure for the synthesis of 5i–m

In the case of the synthesis of compounds 5i–m, H_2_O was used as the reaction medium and the round-bottom flask was fitted with a reflux condenser and heated with stirring in an oil-bath at reflux temperature for a period of time shown in Table 1.

#### 3′-Methyl-6′-(methylamino)-5′-nitro-1′-phenyl-1′H-spiro[indoline-3,4′-pyrano[2,3-*c*]pyrazol]-2-one (5a)

White powder, mp: 268–272 °C, yield 0.342 g (85%); IR (KBr) (*ν*_max_/cm^−1^): 3186, 3060 (NH), 1686 (C=O), 1532 and 1386 (NO_2_), 1223 (C–N), 1015 (C–O) 766 (Ar). ^1^H NMR (300 MHz, DMSO): *δ* 1.58 (3H, s, CH_3_), 3.17 (3H, d, ^3^*J*_HH_ = 4.8 Hz, NCH_3_), 6.87–6.93 (2H, m, ArH), 7.11 (1H, d, ^3^*J*_HH_ = 7.2 Hz, ArH), 7.18 (1H, d, ^3^*J*_HH_ = 7.5 Hz, ArH), 7.34–7.39 (1H, m, ArH), 7.51–7.57 (2H, m, ArH), 7.73 (2H, d, ^3^*J*_HH_ = 8.1 Hz, ArH), 10.71–10.73 (1H, m, NH), 10.74 (1H, s, NH). ^13^C NMR (75.4 MHz, DMSO): *δ* 12.2 (CH_3_), 29.4 (CH_3_N), 50.0 (C_spiro_), 98.3 (C–NO_2_), 107.5 (**C**=C–N), 109.7, 121.1, 121.2, 122.4, 122.6, 123.6, 127.5, 128.9, 130.0, 130.1, 132.1, 137.3 (Ar), 142.9 (C=N), 144.7 (N–N–C–O), 159.4 (O–C–N), 176.6 (CO); MS (EI, 70 eV): *m*/*z* (%) = 403 (9) [M]^+^, 387 (32), 342 (78), 303 (67), 274 (67), 115 (35), 91 (55), 77 (100). Anal. calcd for C_21_H_17_N_5_O_4_ (403.13): C, 62.53; H, 4.25; N, 17.36. Found C, 62.11; H, 3.90; N, 17.18.

#### 5-Chloro-3′-methyl-6′-(methylamino)-5′-nitro-1′-phenyl-1′*H*-spiro[indoline-3,4′-pyrano[2,3-*c*]pyrazol]-2-one (5b)

White powder, mp: 285–287 °C, yield 0.349 g (80%); IR (KBr) (*ν*_max_/cm^−1^): 3372, 3025 (NH), 1686 (C=O), 1498 and 1363 (NO_2_), 1213 (C–N), 1102 (C–O) 776 (Ar), 585 (C–Cl). ^1^H NMR (300 MHz, DMSO): *δ* 1.65 (3H, s, CH_3_), 3.16 (3H, d, ^3^*J*_HH_ = 4.8 Hz, NCH_3_), 6.90 (1H, d, ^3^*J*_HH_ = 8.1 Hz, ArH), 7.24 (1H, d, ^3^*J*_HH_ = 8.4 Hz, ArH), 7.33–7.40 (1H, m, ArH), 7.34 (1H, s, ArH), 7.52–7.57 (2H, m, ArH), 7.72 (2H, d, ^3^*J*_HH_ = 8.1 Hz, ArH), 10.74–10.76 (1H, m, NH), 10.90 (1H, s, NH). ^13^C NMR (75.4 MHz, DMSO): *δ* 12.1 (CH_3_), 29.3 (CH_3_N), 49.9 (C_spiro_), 98.7 (C–NO_2_), 107.4 (**C**=C–N), 109.7, 119.4, 120.8, 122.4, 123.6, 127.2, 128.9, 131.8, 131.9, 134.3, 138.4, 142.9 (Ar), 143.2 (C=N), 145.3 (N–N–C–O), 159.3 (O–C–N), 176.4 (CO). Anal. calcd for C_21_H_16_ClN_5_O_4_ (437.09): C, 57.61; H, 3.68; N, 16.00. Found C, 57.50; H, 3.92; N, 16.41.

#### 5-Bromo-3′-methyl-6′-(methylamino)-5′-nitro-1′-phenyl-1′*H*-spiro[indoline-3,4′-pyrano[2,3-*c*]pyrazol]-2-one (5c)

White powder, mp: 280–282 °C, yield 0.375 g (78%); IR (KBr) (*ν*_max_/cm^−1^): 3340, 3170 (NH), 1687 (C=O), 1489 and 1370 (NO_2_), 1245 (C–N), 1035 (C–O) 776 (Ar), 524 (C–Br). ^1^H NMR (300 MHz, DMSO): *δ* 1.62 (3H, s, CH_3_), 3.16 (3H, d, ^3^*J*_HH_ = 5.1 Hz, NCH_3_), 6.85 (1H, d, ^3^*J*_HH_ = 8.1 Hz, ArH), 7.32–7.42 (3H, m, ArH), 7.46 (1H, s, ArH), 7.62 (1H, d, ^3^*J*_HH_ = 8.4 Hz, ArH), 7.73 (1H, d, ^3^*J*_HH_ = 8.4 Hz, ArH), 10.74–10.76 (1H, m, NH), 10.90 (1H, s, NH). ^13^C NMR (75.4 MHz, DMSO): *δ* 12.1 (CH_3_), 29.4 (CH_3_N), 50.1 (C_spiro_), 97.7 (C–NO_2_), 107.2 (**C**=C–N), 111.6, 114.2, 121.2, 126.7, 127.6, 129.4, 130.1, 131.6, 131.8, 134.6, 137.2, 142.2 (Ar), 143.1 (C=N), 144.5 (N–N–C–O), 159.4 (O–C–N), 176.2 (CO).

#### 1′-(3-Chlorophenyl)-3′-methyl-6′-(methylamino)-5′-nitro-1′*H*-spiro[indoline-3,4′-pyrano[2,3-*c*]pyrazol]-2-one (5d)

White powder, mp: 272–275 °C, yield 0.375 g (86%); IR (KBr) (*ν*_max_/cm^−1^): 3130, 3052 (OH), 1701 (C=O), 1584 and 1397 (NO_2_), 1217 (C–N), 1026 (C–O) 772 (Ar), 542 (C–Cl). ^1^H NMR (300 MHz, DMSO): *δ* 1.60 (3H, s, CH_3_), 3.17 (3H, d, ^3^*J*_HH_ = 6.3 Hz, NCH_3_), 6.87–6.93 (2H, m, ArH), 7.10 (1H, d, ^3^*J*_HH_ = 7.2 Hz, ArH), 7.17–7.22 (1H, m, ArH), 7.43 (1H, d, ^3^*J*_HH_ = 8.1 Hz, ArH), 7.57 (1H, d, ^3^*J*_HH_ = 8.1 Hz, ArH), 7.78 (1H, d, ^3^*J*_HH_ = 7.2 Hz, ArH), 7.84 (1H, s, ArH), 10.70–10.72 (1H, m, NH), 10.75 (1H, s, NH). ^13^C NMR (75.4 MHz, DMSO): *δ* 12.2 (CH_3_), 29.4 (CH_3_N), 50.2 (C_spiro_), 97.7 (C–NO_2_), 107.2 (**C**=C–N), 111.1, 121.1, 121.2, 124.0, 126.5, 127.6, 128.7, 130.0, 130.1, 134.2, 141.8 (Ar), 143.1 (C=N), 144.5 (N–N–C–O), 159.4 (O–C–N), 176.4 (CO); MS (EI, 70 eV): *m*/*z* (%) = 438 (1) [M + 1]^+^, 437 (3) [M]^+^, 391 (25), 376 (100), 363 (23), 209 (24), 111 (32).

#### 5-Bromo-1′-(3-chlorophenyl)-3′-methyl-6′-(methylamino)-5′-nitro-1′*H*-spiro[indoline-3,4′-pyrano[2,3-*c*]pyrazol]-2-one (5e)

White powder, mp: 268–271 °C, yield 0.386 g (75%). ^1^H NMR (300 MHz, DMSO): *δ* 1.62 (3H, s, CH_3_), 3.26 (3H, d, ^3^*J*_HH_ = 4.5 Hz, NCH_3_), 6.85 (1H, d, ^3^*J*_HH_ = 7.8 Hz, ArH), 7.38 (1H, d, ^3^*J*_HH_ = 8.1 Hz, ArH), 7.45 (1H, d, ^3^*J*_HH_ = 8.1 Hz, ArH), 7.50 (1H, s, ArH), 7.56–7.61 (1H, m, ArH), 7.72 (1H, d, ^3^*J*_HH_ = 8.1 Hz, ArH), 7.83 (1H, s, ArH), 10.74–10.83 (1H, m, NH), 10.90 (1H, s, NH). ^13^C NMR (75.4 MHz, DMSO): *δ* 12.2 (CH_3_), 29.4 (CH_3_N), 50.0 (C_spiro_), 98.2 (C–NO_2_), 107.2 (**C**=C–N), 111.7, 114.2, 119.4, 120.8, 126.7, 127.2, 131.7, 131.8, 134.3, 134.4, 138.3, 142.2 (Ar), 143.4 (C=N), 145.2 (N–N–C–O), 159.3 (O–C–N), 176.1 (CO); MS (EI, 70 eV): *m*/*z* (%) = 517 (6) [M + 2]^+^, 515 (5) [M]^+^, 501 (24), 456 (100), 342 (38), 125 (46), 111 (84).

#### 5-Chloro-1′-(3-chlorophenyl)-3′-methyl-6′-(methylamino)-5′-nitro-1′*H*-spiro[indoline-3,4′-pyrano[2,3-*c*]pyrazol]-2-one (5f)

White powder, mp: 254–258 °C, yield 0.376 g (80%). ^1^H NMR (300 MHz, DMSO): *δ* 1.65 (3H, s, CH_3_), 3.18 (3H, d, ^3^*J*_HH_ = 3.6 Hz, NCH_3_), 6.90 (1H, d, ^3^*J*_HH_ = 8.4 Hz, ArH), 7.25 (1H, d, ^3^*J*_HH_ = 8.1 Hz, ArH), 7.32 (1H, s, ArH), 7.45 (1H, d, ^3^*J*_HH_ = 7.5 Hz, ArH), 7.55–7.61 (1H, m, ArH), 7.71 (1H, d, ^3^*J*_HH_ = 8.4 Hz, ArH), 7.83 (1H, s, ArH), 10.68–10.79 (1H, m, NH), 10.89 (1H, s, NH). ^13^C NMR (75.4 MHz, DMSO): *δ* 12.2 (CH_3_), 29.4 (CH_3_N), 50.0 (C_spiro_), 98.1 (C–NO_2_), 107.2 (**C**=C–N), 111.1, 119.5, 120.9, 124.0, 126.5, 127.3, 128.8, 131.8, 134.1, 134.3, 138.3, 141.8 (Ar), 143.4 (C=N), 145.2 (N–N–C–O), 159.3 (O–C–N), 176.2 (CO); MS (EI, 70 eV): *m*/*z* (%) = 472 (5) [M + 1]^+^, 471 (7) [M]^+^, 425 (20), 410 (100), 336 (17), 192 (22), 166 (24), 125 (33), 111 (66).

#### 5-Bromo-1′-methyl-6′-(methylamino)-5′-nitro-3′-(trifluoromethyl)-1′*H*-spiro[indoline-3,4′-pyrano[2,3-*c*]pyrazol]-2-one (5g)

White powder, mp: 298–301 °C, yield 0.292 g (62%). ^1^H NMR (300 MHz, DMSO): *δ* 3.20 (3H, d, ^3^*J*_HH_ = 3.6 Hz, NHCH_3_), 3.86 (3H, s, NCH_3_), 7.04 (1H, d, ^3^*J*_HH_ = 7.2 Hz, ArH), 7.34 (1H, d, ^3^*J*_HH_ = 7.5 Hz, ArH), 7.39 (1H, s, ArH), 10.71–10.76 (1H, m, NH), 10.80 (1H, s, NH). ^13^C NMR (75.4 MHz, DMSO): *δ* 29.2 (CH_3_), 35.6 (CH_3_N), 49.9 (C_spiro_), 96.1 (C–NO_2_), 107.5 (**C**=C–N), 125.0, 111.5, 113.6, 126.6, 131.7, 134.5, 142.7 (Ar), 134.0 (C=N), 145.2 (N–N–C–O), 158.5 (O–C–N), 176.1 (CO).

#### 6′-(Benzylamino)-5-chloro-3′-methyl-5′-nitro-1′-phenyl-1′*H*-spiro[indoline-3,4′-pyrano[2,3-*c*]pyrazol]-2-one (5h)

Red powder, mp: 271–274 °C, yield 0.328 g (58%). ^1^H NMR (300 MHz, DMSO): *δ* 3.10 (3H, d, ^3^*J*_HH_ = 4.2 Hz, NHCH_3_), 3.33–3.45 (2H, m, CH_2_), 7.14–7.64 (8H, m, ArH), 11.20–11.31 (1H, m, NH), 12.53 (1H, s, NH). ^13^C NMR (75.4 MHz, DMSO): *δ* 25.6 (CH_2_), 47.9 (C_spiro_), 99.2 (C–NO_2_), 117.0 (**C**=C–N), 119.8, 120.2, 121.0, 123.5, 130.7, 131.5, 132.2, 132.6, 134.2, 135.7, 136.7 (Ar), 149.9 (C=N), 156.9 (N–N–C–O), 164.7 (O–C–N), 176.0 (CO).

#### 5-Chloro-7′-(methylamino)-6′-nitrospiro[indoline-3,5′-pyrano[2,3-*d*]pyrimidine]-2,2′,4′(1′*H*,3′*H*)-trione (5i)

White powder, mp: 287–293 °C, yield 0.254 g (65%); IR (KBr) (*ν*_max_/cm^−1^): 3410, 3196 (NH), 1730, 1653 (C=O), 1472 and 1371 (NO_2_), 1241 (C–N), 1126 (C–O) 768 (Ar), 560 (C–Cl). ^1^H NMR (300 MHz, DMSO): *δ* 3.08 (3H, d, ^3^*J*_HH_ = 4.8 Hz, NCH_3_), 6.96 (1H, d, ^3^*J*_HH_ = 8.1 Hz, ArH), 7.11 (1H, d, ^3^*J*_HH_ = 8.1 Hz, ArH), 7.34 (1H, s, ArH), 10.51–10.53 (1H, m, NH), 10.66 (1H, s, NH), 11.23 (1H, s, NH), 12.52 (1H, s, NH). ^13^C NMR (75.4 MHz, DMSO): *δ* 29.3 (CH_3_N), 48.7 (C_spiro_), 88.9 (C–NO_2_), 107.5 (**C**=C–O), 110.1, 123.8, 125.4, 128.3, 133.2, 143.9 (Ar), 149.4 (NCON), 152.0 (N–C–O), 157.0 (O–**C**–NCH_3_), 161.3 (CO), 176.6 (CO). Anal. calcd for C_15_H_10_ClN_5_O_6_ (391.03): C, 45.99; H, 2.57; N, 17.88. Found C, 46.11; H, 2.38; N, 18.01.

#### 5-Chloro-6′-nitro-7′-(propylamino)spiro[indoline-3,5′-pyrano[2,3-*d*]pyrimidine]-2,2′,4′(1′*H*,3′*H*)-trione (5j)

White powder, mp: 240–242 °C, yield 0.314 g (75%); IR (KBr) (*ν*_max_/cm^−1^): 3393, 3140 (NH), 1702 (C=O), 1607 (C=O), 1472 and 1372 (NO_2_), 1179 (C–N), 1040 (C–O), 599 (C–Cl). ^1^H NMR (300 MHz, DMSO): *δ* 0.87 (3H, t, ^3^*J*_HH_ = 7.8 Hz, CH_3_), 1.44–1.57 (2H, m, CH_2_), 2.68–2.74 (2H, m, NCH_2_), 6.60 (1H, d, ^3^*J*_HH_ = 8.1 Hz, ArH), 6.92 (1H, s, ArH), 7.03 (1H, d, ^3^*J*_HH_ = 8.1 Hz, ArH), 7.41–7.69 (1H, m, NH), 9.31 (1H, s, NH), 9.80 (1H, s, NH), 9.90 (1H, s, NH). ^13^C NMR (75.4 MHz, DMSO): *δ* 11.3 (CH_3_), 20.8 (CH_2_), 41.0 (NCH_2_), 56.5 (C_spiro_), 77.2 (C–NO_2_), 84.0 (**C**=C–O), 110.5, 123.3, 125.2, 126.1, 127.6, 130.1 (Ar), 139.3 (NCON), 141.6 (N–C–O), 151.9 (O–**C**–NCH_3_), 152.2 (CO), 179.5 (CO); MS (EI, 70 eV): *m*/*z* (%) = 419 (0.01) [M]^+^, 181 (47), 153 (100), 128 (53), 90 (19), 63 (40).

#### 5-Chloro-7′-(isopropylamino)-6′-nitrospiro[indoline-3,5′-pyrano[2,3-*d*]pyrimidine]-2,2′,4′(1′*H*,3′*H*)-trione (5k)

White powder, mp: 270–272 °C, yield 0.318 g (76%); IR (KBr) (*ν*_max_/cm^−1^): 3161, 3046 (NH), 1729, 1650, 1601 (C=O), 1466 and 1373 (NO_2_), 1239 (C–N), 1021 (C–O) 758 (Ar), 550 (C–Cl). ^1^H NMR (300 MHz, DMSO): *δ* 1.12 (6H, d, ^3^*J*_HH_ = 6.6 Hz, CH_3_), 3.19–3.28 (1H, m, CH), 6.61 (1H, d, ^3^*J*_HH_ = 8.1 Hz, ArH), 6.92 (1H, s, ArH), 7.03 (1H, d, ^3^*J*_HH_ = 8.1 Hz, ArH), 7.42–7.68 (1H, m, NH), 9.36 (1H, s, NH), 9.82 (1H, s, NH), 9.88 (1H, s, NH). ^13^C NMR (75.4 MHz, DMSO): *δ* 20.8 (CH_3_), 43.5 (CH), 77.2 (C–NO_2_), 83.9 (**C**=C–O), 110.5, 123.3, 125.2, 127.6 (Ar), 139.3 (NCON), 141.6 (N–C–O), 152.2 (O–**C**–NCH_3_), 152.2 (CO), 179.5 (CO); MS (EI, 70 eV): *m*/*z* (%) = 419 (0.01) [M]^+^, 181 (48), 153 (100), 128 (53), 110 (4), 90 (18), 63 (36).

#### 5-Bromo-6′-nitro-7′-(propylamino)spiro[indoline-3,5′-pyrano[2,3-*d*]pyrimidine]-2,2′,4′(1′*H*,3′*H*)-trione (5l)

White powder, mp: 235–239 °C, yield 0.328 g (71%). ^1^H NMR (300 MHz, DMSO): *δ* 0.90 (3H, t, ^3^*J*_HH_ = 8.1 Hz, CH_3_), 1.47–1.54 (2H, m, CH_2_), 2.65–2.73 (2H, m, NCH_2_), 6.57 (1H, d, ^3^*J*_HH_ = 8.1 Hz, ArH), 7.03 (1H, s, ArH), 7.16 (1H, d, ^3^*J*_HH_ = 8.1 Hz, ArH), 7.38–7.78 (1H, m, NH), 9.30 (1H, s, NH), 9.81 (1H, s, NH), 9.90 (1H, s, NH). ^13^C NMR (75.4 MHz, DMSO): *δ* 11.3 (CH_3_), 20.9 (CH_2_), 41.0 (NCH_2_), 56.7 (C_spiro_), 77.2 (C–NO_2_), 83.8 (**C**=C–O), 111.0, 112.8, 125.5, 126.0, 130.4 (Ar), 139.9 (NCON), 142.0 (N–C–O), 143.8 (O–**C**–NCH_3_), 152.1 (CO), 179.3 (CO).

#### 5-Bromo-7′-(isopropylamino)-6′-nitrospiro[indoline-3,5′-pyrano[2,3-*d*]pyrimidine]-2,2′,4′(1′*H*,3′*H*)-trione (5m)

White powder, mp: 260–262 °C, yield 0.333 g (72%). ^1^H NMR (300 MHz, DMSO): *δ* 1.12 (6H, d, ^3^*J*_HH_ = 6.3 Hz, CH_3_), 3.21–3.33 (1H, m, CH), 6.67 (1H, d, ^3^*J*_HH_ = 8.1 Hz, ArH), 7.03 (1H, s, ArH), 7.16 (1H, d, ^3^*J*_HH_ = 8.1 Hz, ArH), 7.55–7.81 (1H, m, NH), 9.32 (1H, s, NH), 9.82 (1H, s, NH), 9.89 (1H, s, NH). ^13^C NMR (75.4 MHz, DMSO): *δ* 20.8 (CH_3_), 43.5 (CH), 44.3 (C_spiro_), 77.2 (C–NO_2_), 83.9 (**C**=C–O), 111.1, 112.9, 117.2, 124.0, 126.1, 130.4 (Ar), 139.7 (NCON), 142.0 (N–C–O), 152.1 (O–**C**–NCH_3_), 162.0 (CO), 179.3 (CO).

#### 25′-Chloro-2-(methylamino)-3-nitro-5*H*-spiro[indeno[1,2-*b*]pyran-4,3′-indoline]-2′,5-dione (5n)

Red powder, mp: 238–240 °C, yield 0.265 g (65%). ^1^H NMR (300 MHz, DMSO): *δ* 3.39 (3H, d, ^3^*J*_HH_ = 7.2 Hz, CH_3_), 7.11–7.63 (7H, m, ArH), 11.21–11.32 (1H, m, NH), 12.53 (1H, s, NH). ^13^C NMR (75.4 MHz, DMSO): *δ* 25.6 (CH_3_), 48.0 (C_spiro_), 89.8 (C–NO_2_), 99.1 (**C**=C–O), 119.7, 120.2, 120.9, 127.9, 129.0, 131.5, 132.2, 134.5, 135.8, 136.3, 149.8 (Ar), 156.9 (O–C–N), 164.7 (C=**C**–O), 174.9 (NCO), 188.6 (CO).

#### 2-(Benzylamino)-3-nitrospiro[benzo[*g*]chromene-4,3′-indoline]-2′,5,10-trione (5o)

Red powder, mp: >300 °C, yield 0.311 g (65%). ^1^H NMR (300 MHz, DMSO): *δ* 4.79–4.89 (2H, m, CH_2_), 6.76–6.81 (1H, m, ArH), 7.13 (1H, d, ^3^*J*_HH_ = 6.6 Hz, ArH), 7.32 (1H, d, ^3^*J*_HH_ = 6.3 Hz, ArH), 7.37–7.41 (1H, m, ArH), 7.58 (1H, d, ^3^*J*_HH_ = 7.5 Hz, ArH), 7.71–7.84 (2H, m, ArH), 8.05 (1H, d, ^3^*J*_HH_ = 6.9 Hz, ArH), 10.74 (1H, s, NH), 10.92–11.03 (1H, m, NH). ^13^C NMR (75.4 MHz, DMSO): *δ* 45.6 (CH_3_), 50.2 (C_spiro_), 107.5 (C–NO_2_), 109.4 (**C**=C–O), 121.7, 124.1, 126.5, 126.8, 128.2, 129.1, 129.3, 131.2, 131.3, 135.4, 137.9, 144.7 (Ar), 148.6 (O–C–N), 156.3 (C=**C**–O), 176.2 (NCO), 176.5 (CO), 181.4 (CO).

#### 5′-Chloro-3-nitro-2-(propylamino)spiro[benzo[*g*]chromene-4,3′-indoline]-2′,5,10-trione (5p)

Red powder, mp: >300 °C, yield 0.288 g (62%); IR (KBr) (*ν*_max_/cm^−1^): 3214, 3061 (NH), 1731, 1651, 1605 (C=O), 1524 and 1352 (NO_2_), 1214 (C–N), 1067 (C–O) 768 (Ar), 542 (C–Cl). ^1^H NMR (300 MHz, DMSO): *δ* 0.85 (3H, t, ^3^*J*_HH_ = 8.1 Hz, CH_3_), 1.50–1.60 (2H, m, CH_2_), 2.62–2.77 (2H, m, NCH_2_), 6.88 (1H, d, ^3^*J*_HH_ = 8.4 Hz, ArH), 7.30 (1H, d, ^3^*J*_HH_ = 8.4 Hz, ArH), 7.41 (1H, s, ArH), 7.60 (1H, d, ^3^*J*_HH_ = 7.8 Hz, ArH), 7.67–7.72 (2H, m, ArH), 7.82 (1H, d, ^3^*J*_HH_ = 7.8 Hz, ArH), 10.28–10.67 (1H, m, NH), 10.89 (1H, s, NH). ^13^C NMR (75.4 MHz, DMSO): *δ* 11.5 (CH_3_), 21.1 (CH_2_), 41.2 (NCH_2_), 55.0 (C_spiro_), 104.2 (C–NO_2_), 112.7 (**C**=C–O), 125.7, 126.1, 128.1, 131.4, 131.8, 133.4, 134.5 (Ar), 147.6 (O–C–N), 155.5 (C=**C**–O), 176.5 (NCO), 177.0 (CO), 182.0 (CO).

#### 5′-Bromo-3-nitro-2-(propylamino)spiro[benzo[*g*]chromene-4,3′-indoline]-2′,5,10-trione (5q)

Red powder, mp: >300 °C, yield 0.305 g (60%). ^1^H NMR (300 MHz, DMSO): *δ* 0.82 (3H, t, ^3^*J*_HH_ = 8.4 Hz, CH_3_), 1.50–1.60 (2H, m, CH_2_), 2.62–2.80 (2H, m, NCH_2_), 6.83 (1H, d, ^3^*J*_HH_ = 8.4 Hz, ArH), 7.43 (1H, d, ^3^*J*_HH_ = 8.4 Hz, ArH), 7.53 (1H, s, ArH), 7.55 (1H, d, ^3^*J*_HH_ = 7.5 Hz, ArH), 7.62–7.72 (2H, m, ArH), 7.82 (1H, d, ^3^*J*_HH_ = 7.2 Hz, ArH), 10.28–10.65 (1H, m, NH), 10.90 (1H, s, NH). ^13^C NMR (75.4 MHz, DMSO): *δ* 11.4 (CH_3_), 20.2 (CH_2_), 41.0 (NCH_2_), 56.5 (C_spiro_), 101.9 (C–NO_2_), 112.7 (**C**=C–O), 125.7, 126.1, 128.1, 131.4, 131.8, 133.4, 134.5 (Ar), 142.8 (O–C–N), 155.2 (C=**C**–O), 173.0 (NCO), 176.5 (CO), 207.0 (CO).

## Conflicts of interest

The authors declare no competing financial interest.

## Supplementary Material

RA-009-C9RA03214B-s001
