# Supplementary material for: Catalyst-free four-component domino synthetic approach toward versatile multicyclic spirooxindole pyran scaffolds
Source: RSC Adv. 2019 May 28;9(29):16525–33. doi: 10.1039/c9ra03214b (PMC9064459; doi:10.1039/c9ra03214b)
Supplement: RA-009-C9RA03214B-s001 [file RA-009-C9RA03214B-s001.pdf]

Electronic Supplementary Material (ESI) for RSC Advances.
This journal is © The Royal Society of Chemistry 2019

# Supporting Information

## Catalyst-free four-component domino synthetic approach toward versatile multicyclic spirooxindole pyran scaffolds

Aref Mohammadi, Mohammad Bayat*, Shima Nasri

*Department of Chemistry, Imam Khomeini International University, Qazvin, Iran.*

*bayat_mo@yahoo.com*

**The Table of Contents**


| Title | Page |
|---|---|
| Title, author's name, address and table of contents | 1 |
| General remarks and **FIGURE 1**. | 2 |
| $^{1}H$ and $^{13}C$ NMR and IR and Mass spectra of **5a** | 3-6 |
| $^{1}H$ and $^{13}C$ NMR spectra of **5b** | 7-8 |
| $^{1}H$ and $^{13}C$ NMR spectra of **5c** | 9-10 |
| $^{1}H$ and $^{13}C$ NMR and IR and Mass spectra of **5d** | 11-14 |
| $^{1}H$ and $^{13}C$ NMR and Mass spectra of **5e** | 15-17 |
| $^{1}H$ and $^{13}C$ NMR and Mass spectra of **5f** | 18-20 |
| $^{1}H$ and $^{13}C$ NMR spectra of **5g** | 21-22 |
| $^{1}H$ and $^{13}C$ NMR spectra of **5h** | 23-24 |
| $^{1}H$ and $^{13}C$ NMR and IR spectra of **5i** | 25-27 |
| $^{1}H$ and $^{13}C$ NMR and IR and Mass spectra of **5j** | 28-31 |
| $^{1}H$ and $^{13}C$ NMR and IR and Mass spectra of **5k** | 32-35 |
| $^{1}H$ and $^{13}C$ NMR spectra of **5l** | 36-37 |
| $^{1}H$ and $^{13}C$ NMR spectra of **5m** | 38-39 |
| $^{1}H$ and $^{13}C$ NMR spectra of **5n** | 40-41 |
| $^{1}H$ and $^{13}C$ NMR spectra of **5o** | 42-43 |
| $^{1}H$ NMR and IR spectra of **5p** | 44-45 |
| $^{1}H$ and $^{13}C$ NMR spectra of **5q** | 46-47 |

## Experimental Section

### General remarks:

The nitroketene dithioacetals, isatin derivatives, various amines, various active methylene compounds and solvents were obtained from Sigma Aldrich and Fluka Co. which used without further purification. IR spectra: Bruker Tensor 27 spectrometer. NMR spectra: Bruker DRX-300 Avance instrument (300 MHz for $^{1}H$ and 75.4 MHz for $^{13}C$) with DMSO-$d_6$ as solvents. Chemical shifts are expressed in parts per million (ppm), and coupling constant (*J*) are reported in hertz (Hz). Mass spectra: Agilent 5975C VL MSD with Triple-Axis detector operating at an ionization potential of 70 eV. Elemental analyses for C, H and N: Heraeus CHNO-Rapid analyzer. Melting points: electrothermal 9100 apparatus.

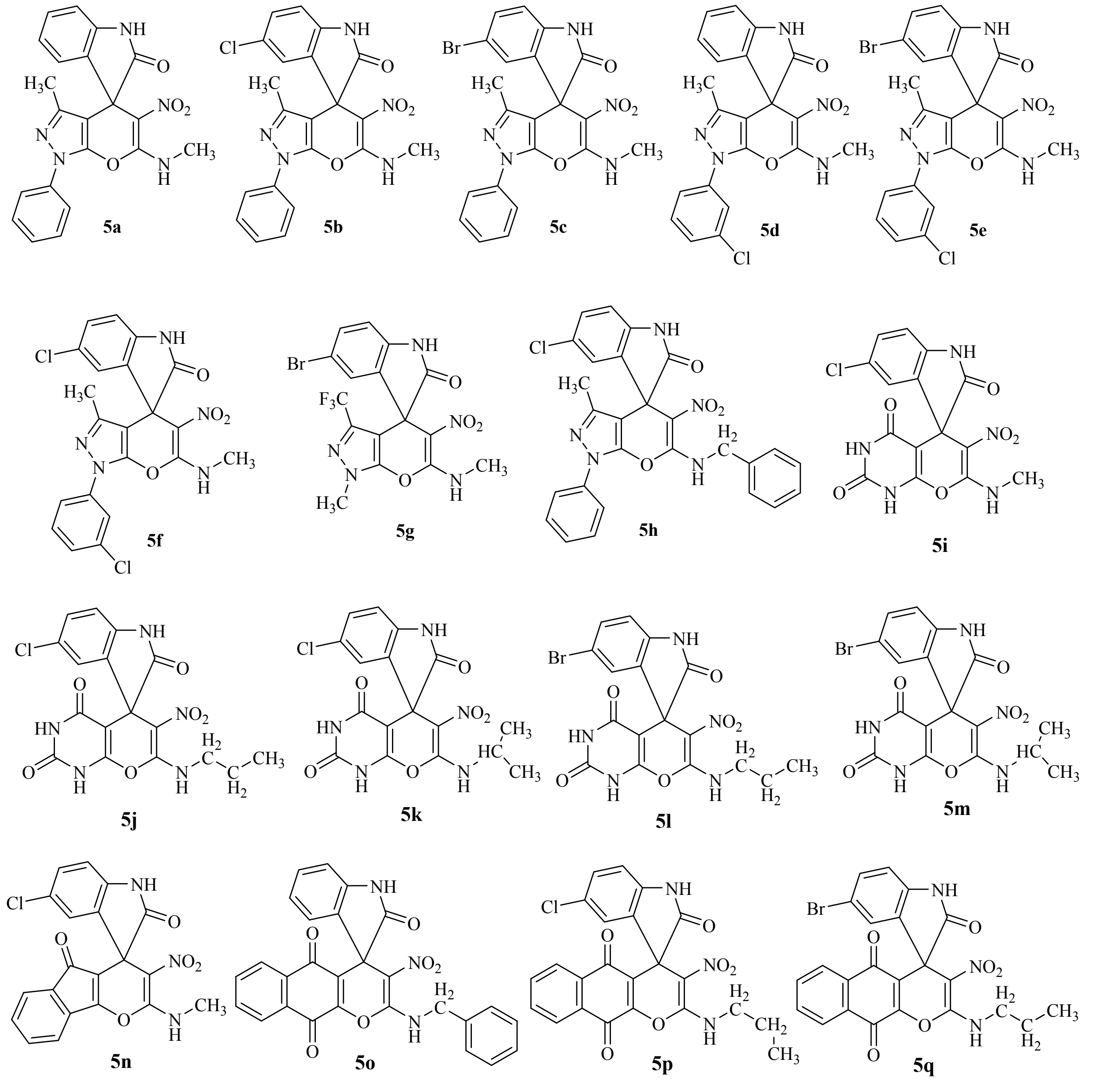


**FIGURE 1**. Structure of all products **5a-q**.

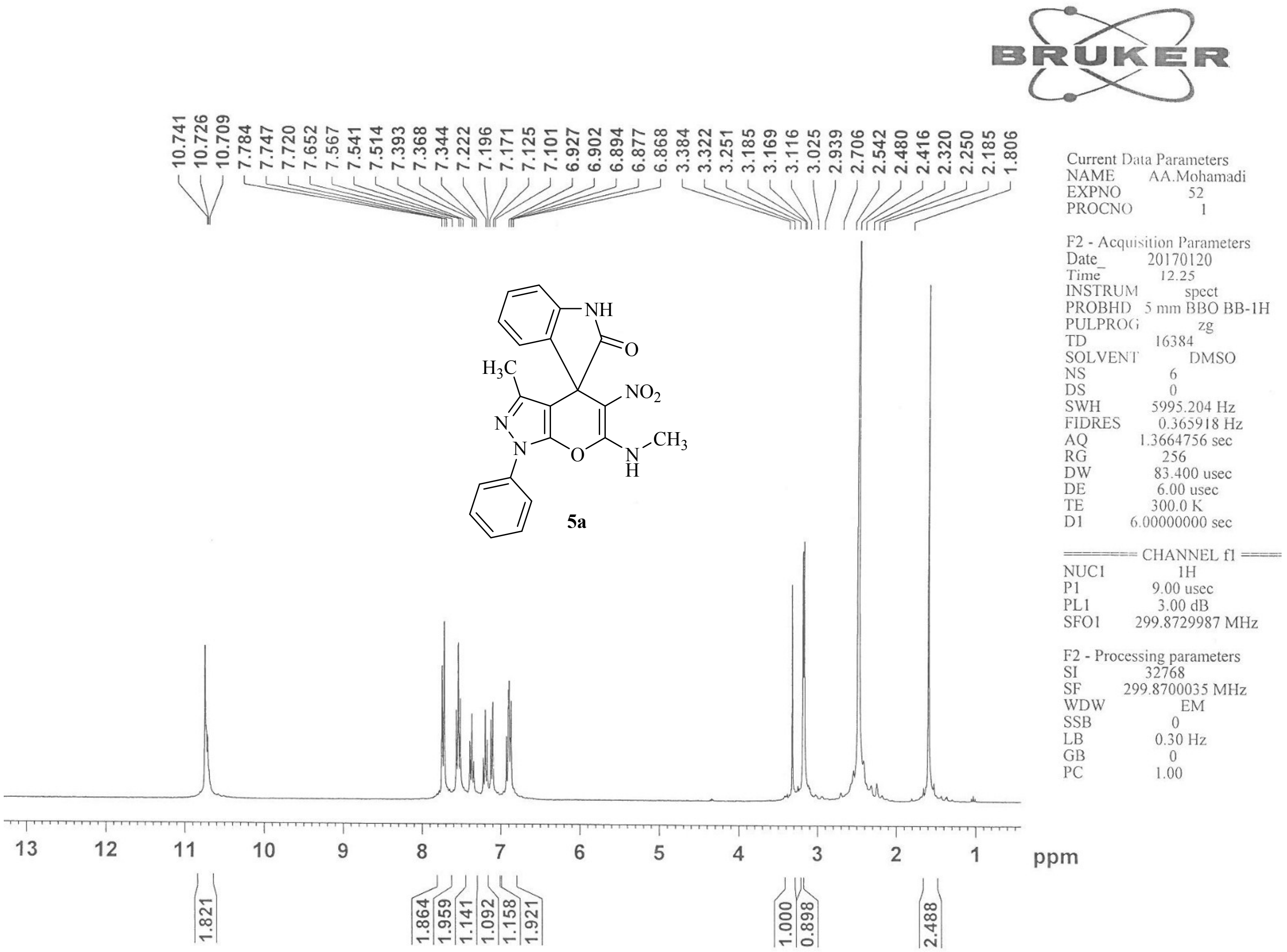

10.741 10.726 10.709 7.784 7.747 7.720 7.652 7.567 7.541 7.514 7.393 7.368 7.344 7.222 7.196 7.171 7.125 7.101 6.927 6.902 6.894 6.877 6.868 3.384 3.322 3.251 3.185 3.169 3.116 3.025 2.939 2.706 2.542 2.480 2.416 2.320 2.250 2.185 1.806
NH
O
$H_3C$
$NO_2$
N
N
O
N
$CH_3$
H
5a
Current Data Parameters
NAME AA.Mohamadi
EXPNO 52
PROCNO 1
F2 - Acquisition Parameters
Date_ 20170120
Time 12.25
INSTRUM spect
PROBHD 5 mm BBO BB-1H
PULPROG zg
TD 16384
SOLVENT DMSO
NS 6
DS 0
SWH 5995.204 Hz
FIDRES 0.365918 Hz
AQ 1.3664756 sec
RG 256
DW 83.400 usec
DE 6.00 usec
TE 300.0 K
D1 6.00000000 sec
======== CHANNEL f1 ========
NUC1 1H
P1 9.00 usec
PL1 3.00 dB
SFO1 299.8729987 MHz
F2 - Processing parameters
SI 32768
SF 299.8700035 MHz
WDW EM
SSB 0
LB 0.30 Hz
GB 0
PC 1.00
13 12 11 10 9 8 7 6 5 4 3 2 1 ppm
1.821
1.864 1.959 1.141 1.092 1.158 1.921
1.000 0.898
2.488


**$^1$H NMR of 5a**

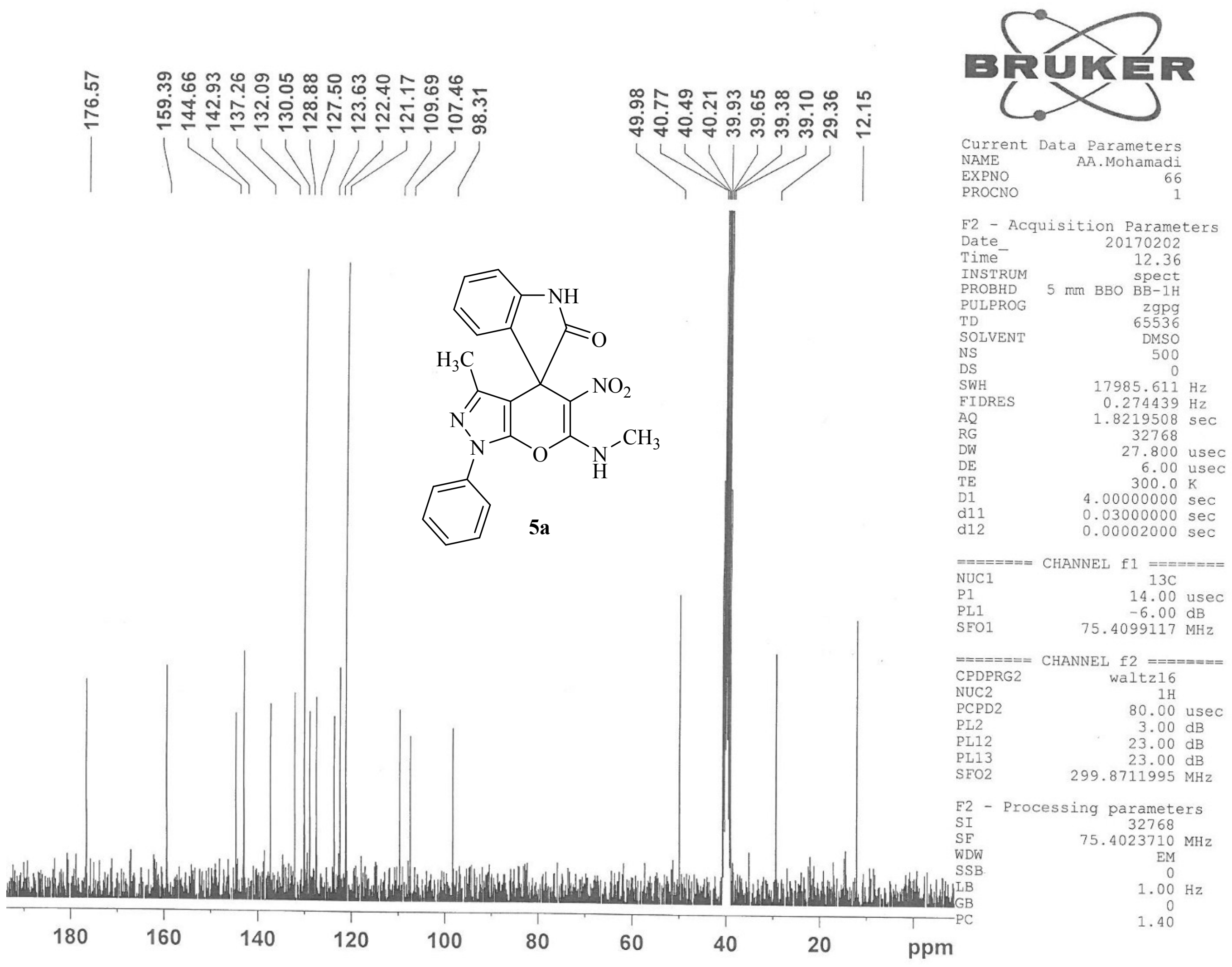

176.57
159.39
144.66
142.93
137.26
132.09
130.05
128.88
127.50
123.63
122.40
121.17
109.69
107.46
98.31
49.98
40.77
40.49
40.21
39.93
39.65
39.38
39.10
29.36
12.15
NH
O
H3C
NO2
N
N
O
N
H
CH3
5a
BRUKER
Current Data Parameters
NAME AA.Mohamadi
EXPNO 66
PROCNO 1
F2 - Acquisition Parameters
Date_ 20170202
Time 12.36
INSTRUM spect
PROBHD 5 mm BBO BB-1H
PULPROG zgpg
TD 65536
SOLVENT DMSO
NS 500
DS 0
SWH 17985.611 Hz
FIDRES 0.274439 Hz
AQ 1.8219508 sec
RG 32768
DW 27.800 usec
DE 6.00 usec
TE 300.0 K
D1 4.00000000 sec
d11 0.03000000 sec
d12 0.00002000 sec
======== CHANNEL f1 ========
NUC1 13C
P1 14.00 usec
PL1 -6.00 dB
SFO1 75.4099117 MHz
======== CHANNEL f2 ========
CPDPRG2 waltz16
NUC2 1H
PCPD2 80.00 usec
PL2 3.00 dB
PL12 23.00 dB
PL13 23.00 dB
SFO2 299.8711995 MHz
F2 - Processing parameters
SI 32768
SF 75.4023710 MHz
WDW EM
SSB 0
LB 1.00 Hz
GB 0
PC 1.40
180
160
140
120
100
80
60
40
20
ppm


**$^{13}$C NMR of 5a**

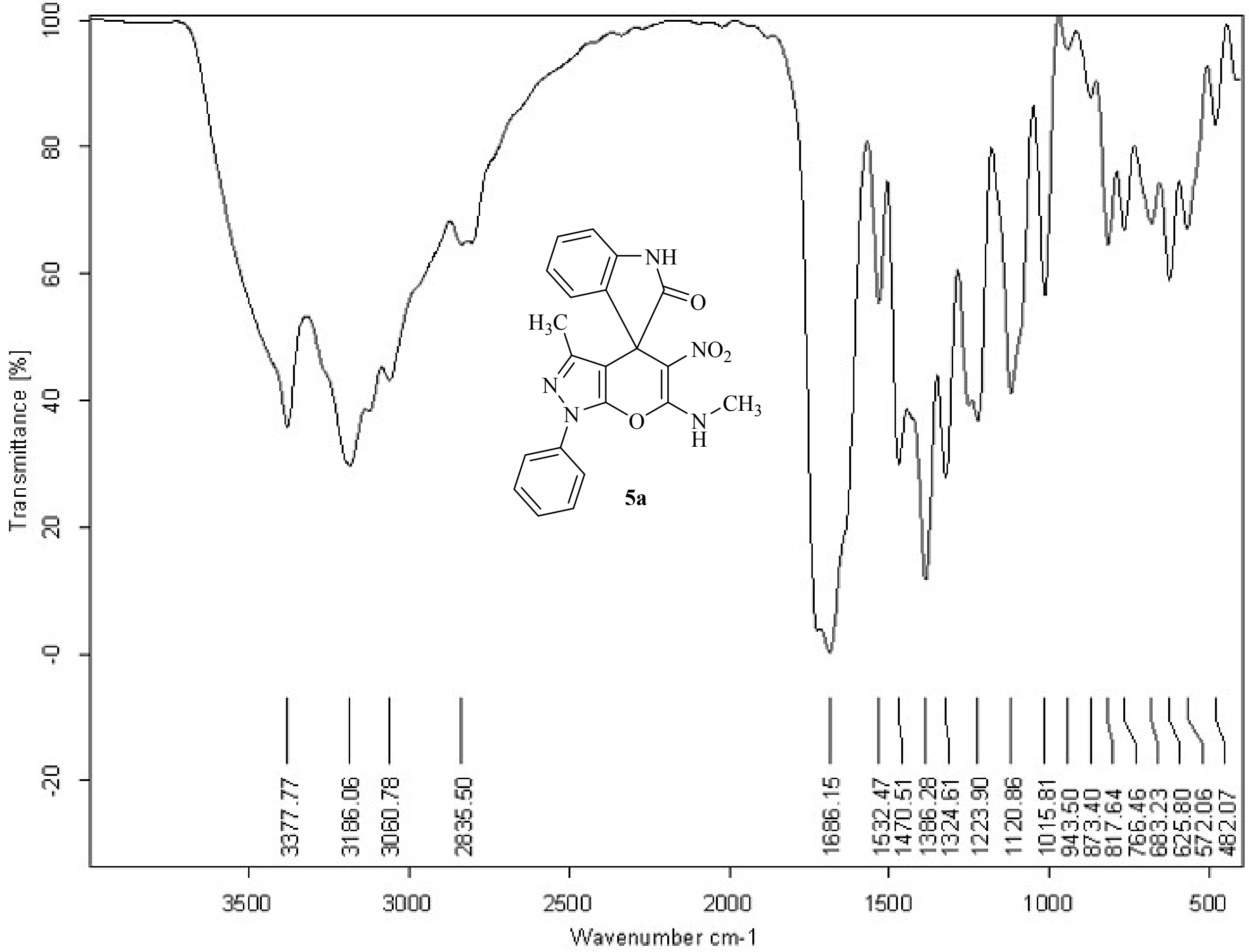

100
80
60
40
20
-0
-20
Transmittance [%]
3377.77
3186.06
3060.78
2835.50
1686.15
1532.47
1470.51
1386.28
1324.61
1223.90
1120.86
1015.81
943.50
873.40
817.64
766.46
683.23
625.80
572.06
482.07
3500
3000
2500
2000
1500
1000
500
Wavenumber cm-1
NH
O
H3C
NO2
N
N
O
N
CH3
H
5a


**IR of 5a**

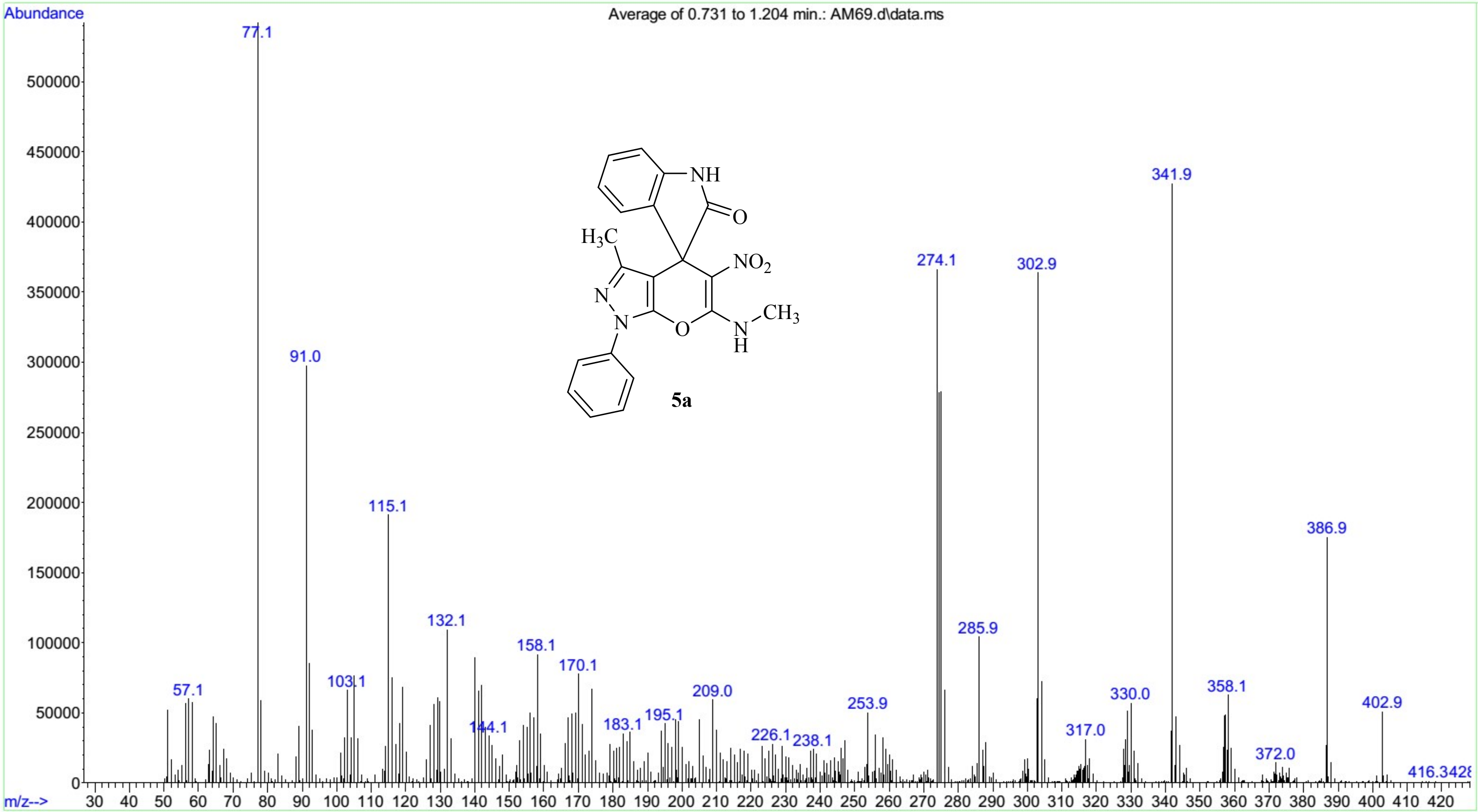
Abundance
Average of 0.731 to 1.204 min.: AM69.d\data.ms
77.1
91.0
115.1
132.1
158.1
170.1
57.1
103.1
144.1
183.1
195.1
209.0
226.1
238.1
253.9
274.1
285.9
302.9
317.0
330.0
341.9
358.1
372.0
386.9
402.9
416.3428
NH
O
$H_3C$
$NO_2$
N
N
O
N
H
$CH_3$
5a
m/z-->


**Mass of 5a**

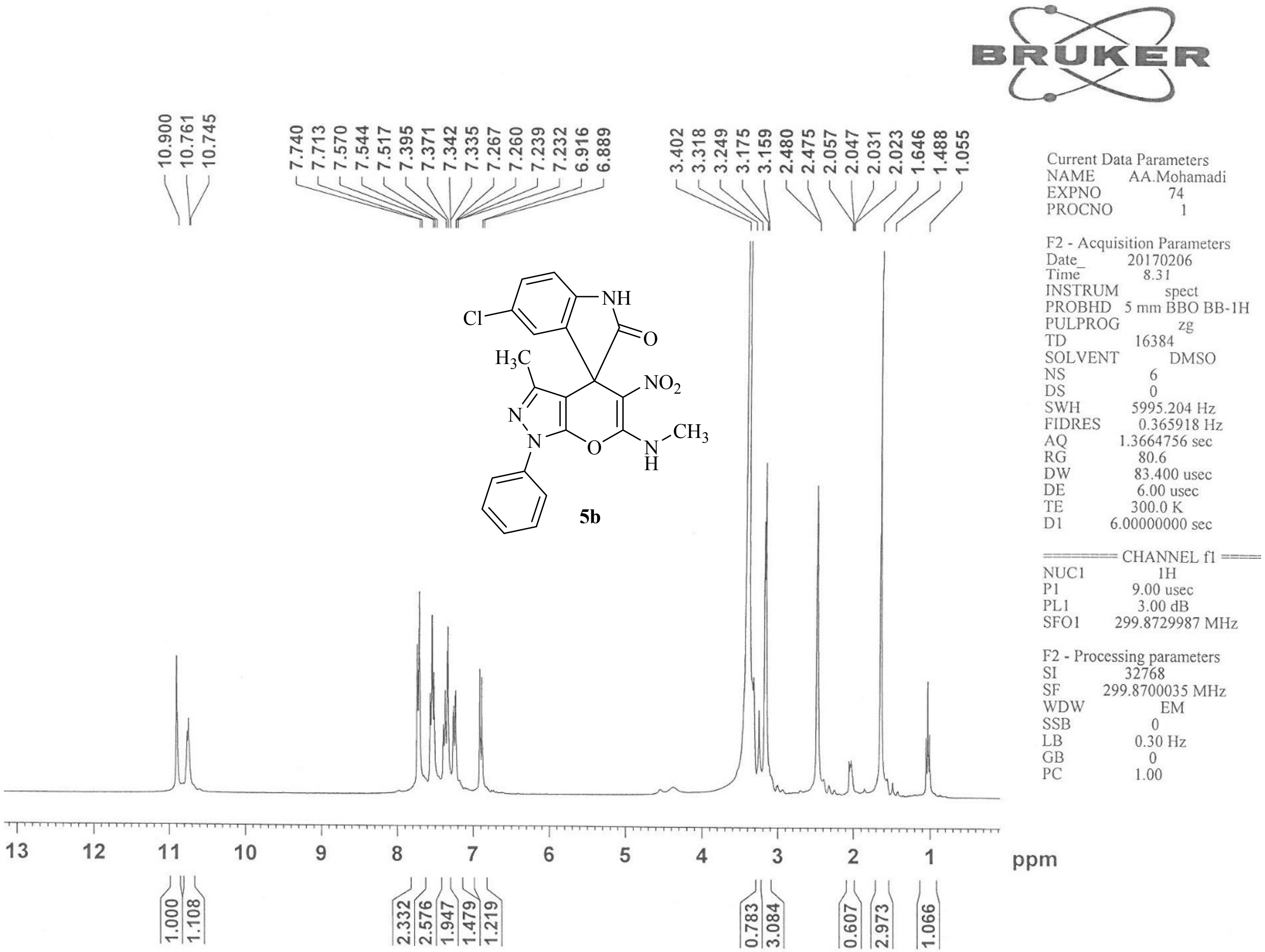
BRUKER
10.900
10.761
10.745
7.740
7.713
7.570
7.544
7.517
7.395
7.371
7.342
7.335
7.267
7.260
7.239
7.232
6.916
6.889
3.402
3.318
3.249
3.175
3.159
2.480
2.475
2.057
2.047
2.031
2.023
1.646
1.488
1.055
Current Data Parameters
NAME AA.Mohamadi
EXPNO 74
PROCNO 1
F2 - Acquisition Parameters
Date_ 20170206
Time 8.31
INSTRUM spect
PROBHD 5 mm BBO BB-1H
PULPROG zg
TD 16384
SOLVENT DMSO
NS 6
DS 0
SWH 5995.204 Hz
FIDRES 0.365918 Hz
AQ 1.3664756 sec
RG 80.6
DW 83.400 usec
DE 6.00 usec
TE 300.0 K
D1 6.00000000 sec
CHANNEL f1
NUC1 1H
P1 9.00 usec
PL1 3.00 dB
SFO1 299.8729987 MHz
F2 - Processing parameters
SI 32768
SF 299.8700035 MHz
WDW EM
SSB 0
LB 0.30 Hz
GB 0
PC 1.00
Cl
NH
O
H3C
NO2
N
N
O
N
H
CH3
5b
13
12
11
10
9
8
7
6
5
4
3
2
1
ppm
1.000
1.108
2.332
2.576
1.947
1.479
1.219
0.783
3.084
0.607
2.973
1.066

**$^1$H NMR of 5b**

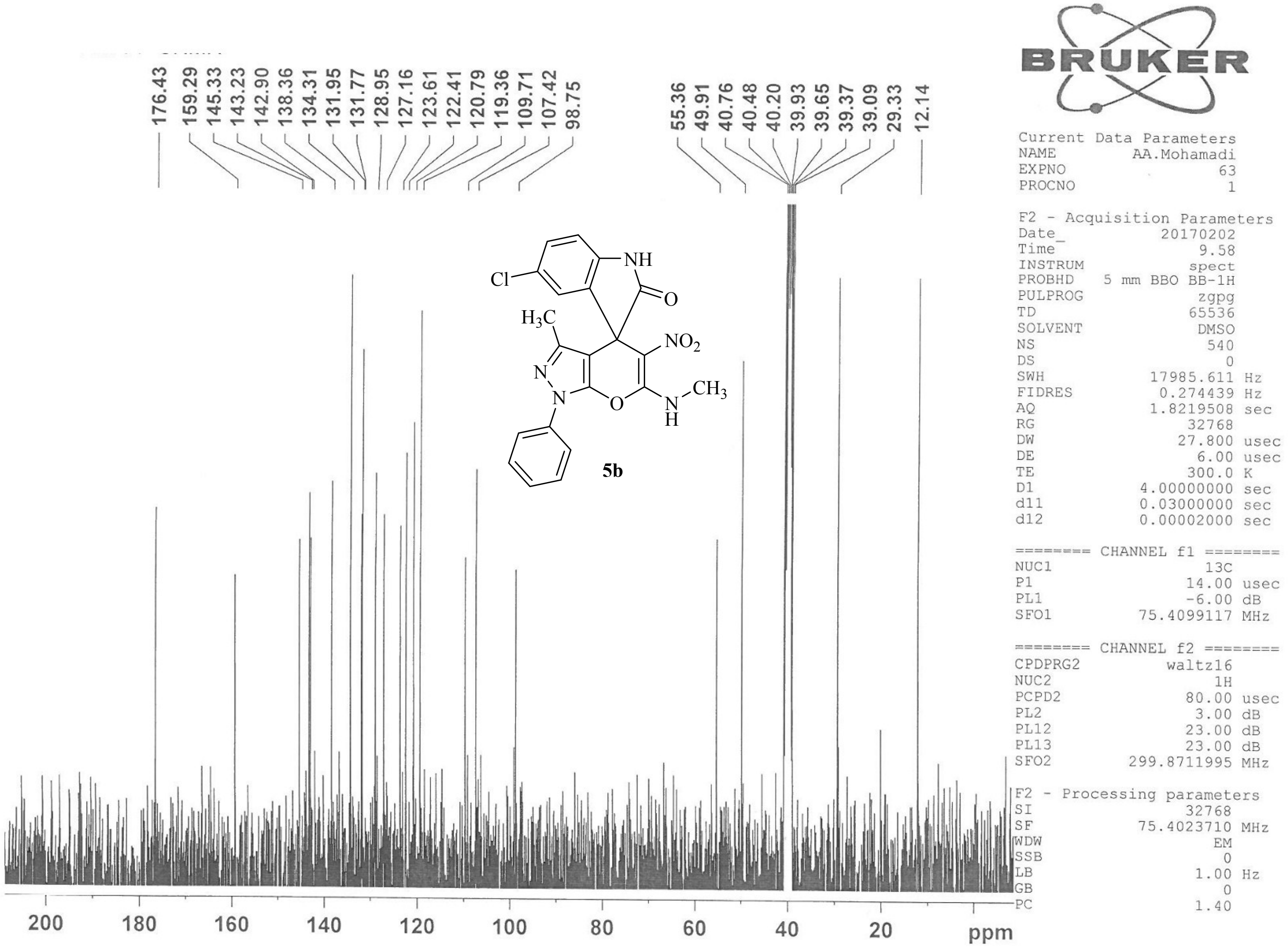
176.43
159.29
145.33
143.23
142.90
138.36
134.31
131.95
131.77
128.95
127.16
123.61
122.41
120.79
119.36
109.71
107.42
98.75
55.36
49.91
40.76
40.48
40.20
39.93
39.65
39.37
39.09
29.33
12.14
NH
O
Cl
$H_3C$
$NO_2$
N
N
O
N
$CH_3$
H
5b
BRUKER
Current Data Parameters
NAME AA.Mohamadi
EXPNO 63
PROCNO 1
F2 - Acquisition Parameters
Date_ 20170202
Time 9.58
INSTRUM spect
PROBHD 5 mm BBO BB-1H
PULPROG zgpg
TD 65536
SOLVENT DMSO
NS 540
DS 0
SWH 17985.611 Hz
FIDRES 0.274439 Hz
AQ 1.8219508 sec
RG 32768
DW 27.800 usec
DE 6.00 usec
TE 300.0 K
D1 4.00000000 sec
d11 0.03000000 sec
d12 0.00002000 sec
======== CHANNEL f1 ========
NUC1 13C
P1 14.00 usec
PL1 -6.00 dB
SFO1 75.4099117 MHz
======== CHANNEL f2 ========
CPDPRG2 waltz16
NUC2 1H
PCPD2 80.00 usec
PL2 3.00 dB
PL12 23.00 dB
PL13 23.00 dB
SFO2 299.8711995 MHz
F2 - Processing parameters
SI 32768
SF 75.4023710 MHz
WDW EM
SSB 0
LB 1.00 Hz
GB 0
PC 1.40
200
180
160
140
120
100
80
60
40
20
ppm

**$^{13}$C NMR of 5b**

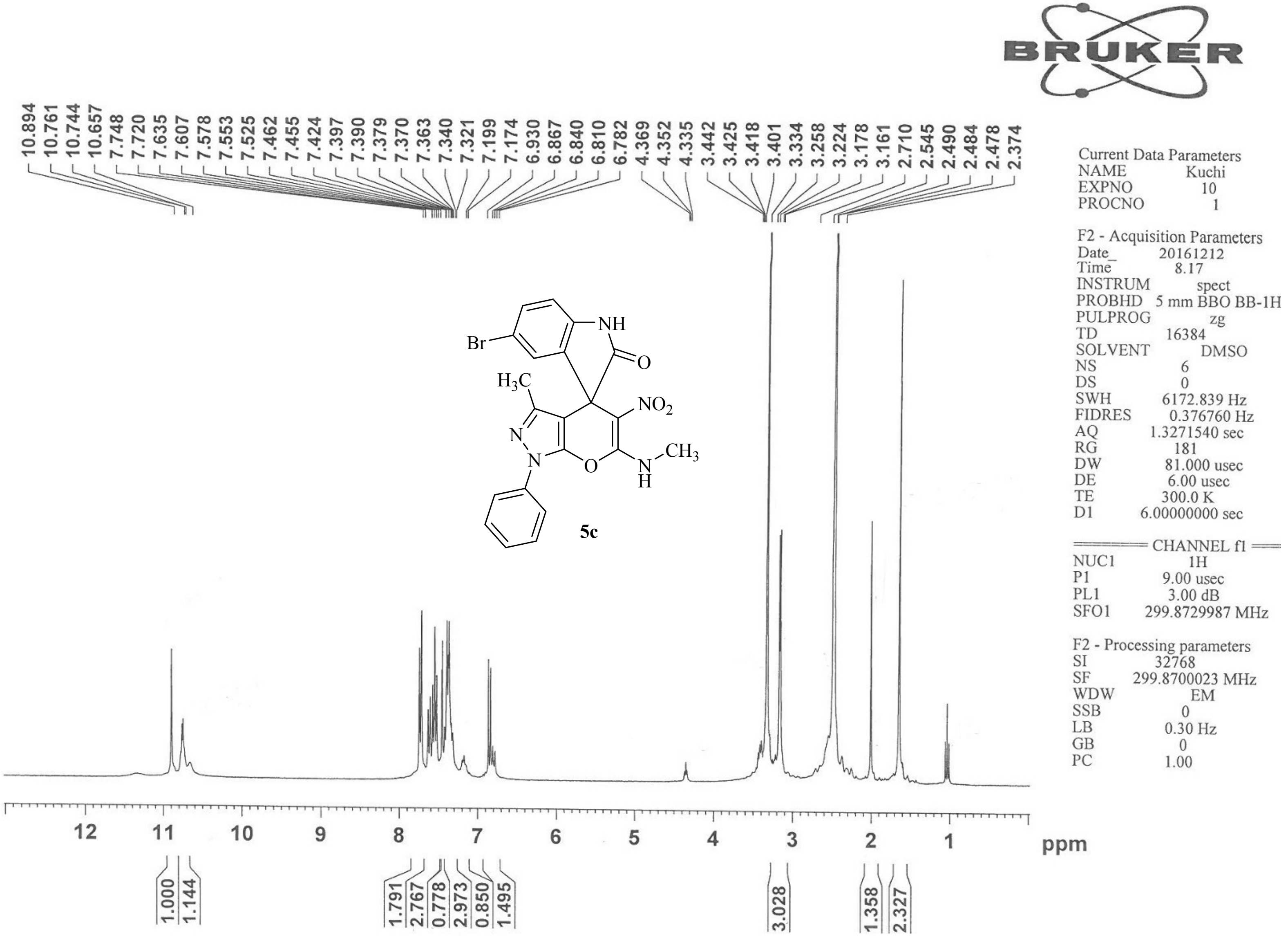

**$^{1}$H NMR of 5c**

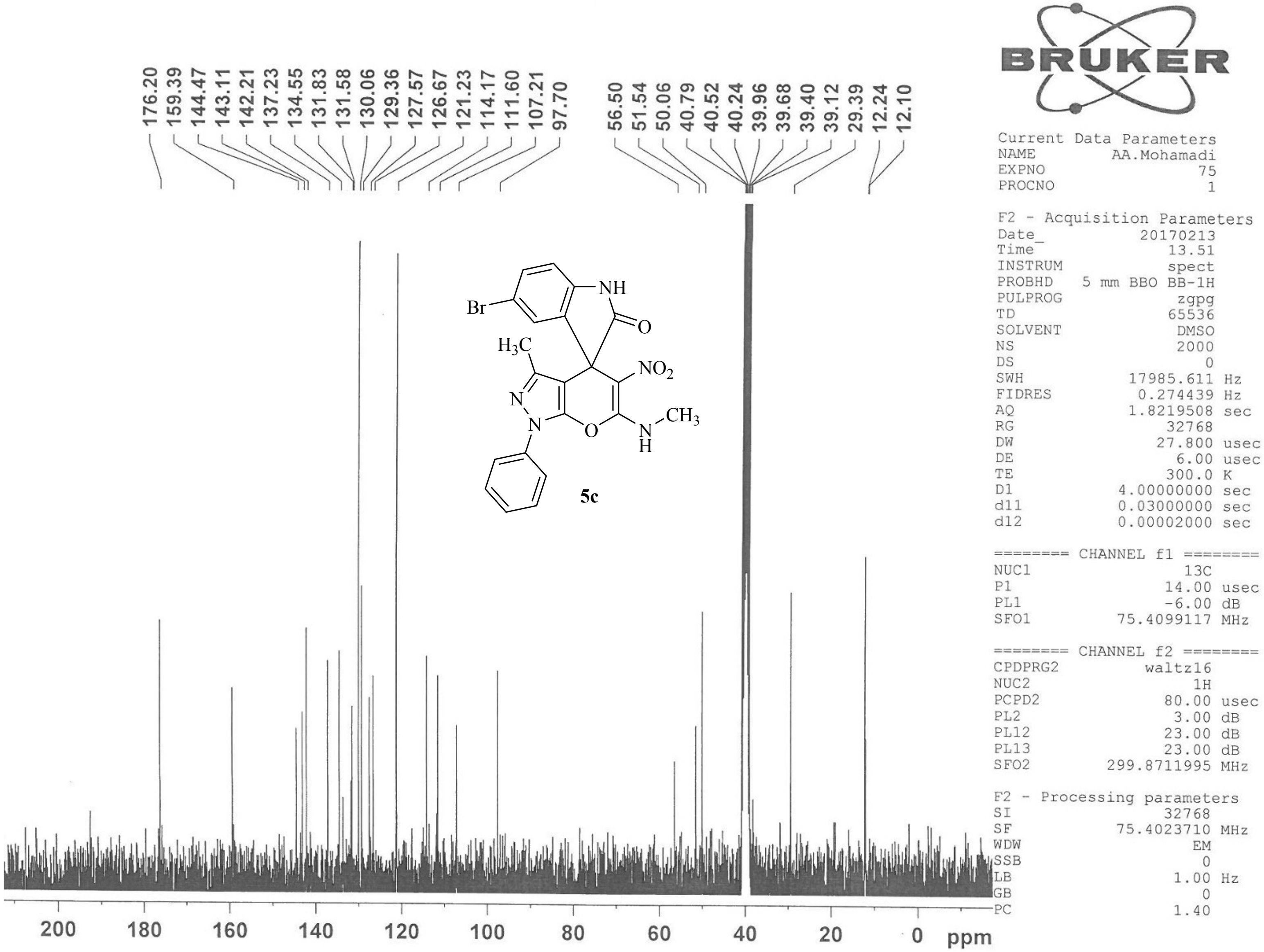
176.20
159.39
144.47
143.11
142.21
137.23
134.55
131.83
131.58
130.06
129.36
127.57
126.67
121.23
114.17
111.60
107.21
97.70
56.50
51.54
50.06
40.79
40.52
40.24
39.96
39.68
39.40
39.12
29.39
12.24
12.10
Br
NH
O
$H_3C$
$NO_2$
N
N
O
N
H
$CH_3$
5c
200
180
160
140
120
100
80
60
40
20
0
ppm
BRUKER
Current Data Parameters
NAME AA.Mohamadi
EXPNO 75
PROCNO 1
F2 - Acquisition Parameters
Date_ 20170213
Time 13.51
INSTRUM spect
PROBHD 5 mm BBO BB-1H
PULPROG zgpg
TD 65536
SOLVENT DMSO
NS 2000
DS 0
SWH 17985.611 Hz
FIDRES 0.274439 Hz
AQ 1.8219508 sec
RG 32768
DW 27.800 usec
DE 6.00 usec
TE 300.0 K
D1 4.00000000 sec
d11 0.03000000 sec
d12 0.00002000 sec
======== CHANNEL f1 ========
NUC1 13C
P1 14.00 usec
PL1 -6.00 dB
SFO1 75.4099117 MHz
======== CHANNEL f2 ========
CPDPRG2 waltz16
NUC2 1H
PCPD2 80.00 usec
PL2 3.00 dB
PL12 23.00 dB
PL13 23.00 dB
SFO2 299.8711995 MHz
F2 - Processing parameters
SI 32768
SF 75.4023710 MHz
WDW EM
SSB 0
LB 1.00 Hz
GB 0
PC 1.40

**$^{13}$C NMR of 5c**

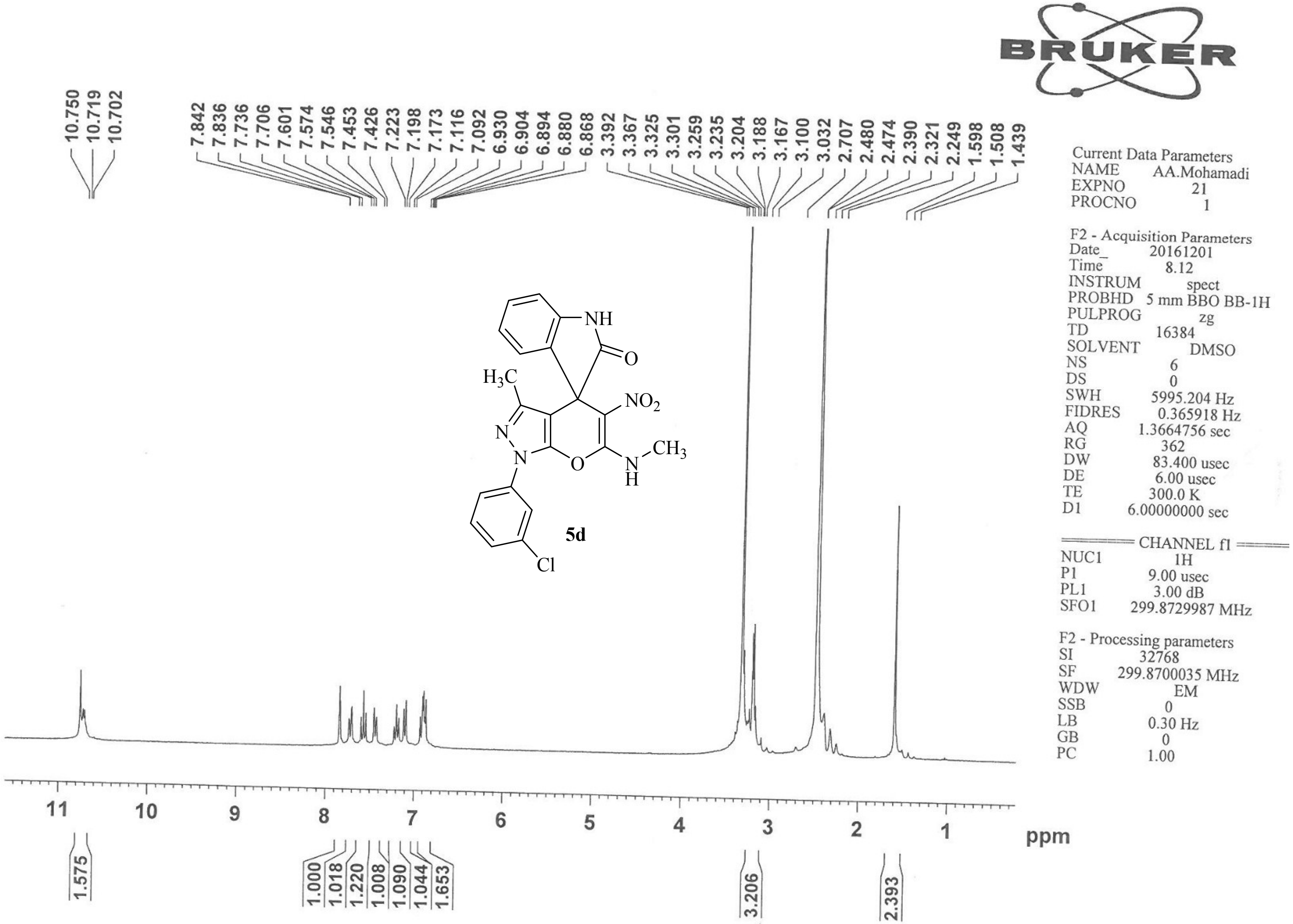
BRUKER
10.750
10.719
10.702
7.842
7.836
7.736
7.706
7.601
7.574
7.546
7.453
7.426
7.223
7.198
7.173
7.116
7.092
6.930
6.904
6.894
6.880
6.868
3.392
3.367
3.325
3.301
3.259
3.235
3.204
3.188
3.167
3.100
3.032
2.707
2.480
2.474
2.390
2.321
2.249
1.598
1.508
1.439
NH
O
$H_3C$
$NO_2$
N
N
O
N
$CH_3$
H
Cl
5d
Current Data Parameters
NAME AA.Mohamadi
EXPNO 21
PROCNO 1
F2 - Acquisition Parameters
Date_ 20161201
Time 8.12
INSTRUM spect
PROBHD 5 mm BBO BB-1H
PULPROG zg
TD 16384
SOLVENT DMSO
NS 6
DS 0
SWH 5995.204 Hz
FIDRES 0.365918 Hz
AQ 1.3664756 sec
RG 362
DW 83.400 usec
DE 6.00 usec
TE 300.0 K
D1 6.00000000 sec
CHANNEL f1
NUC1 1H
P1 9.00 usec
PL1 3.00 dB
SFO1 299.8729987 MHz
F2 - Processing parameters
SI 32768
SF 299.8700035 MHz
WDW EM
SSB 0
LB 0.30 Hz
GB 0
PC 1.00
11 10 9 8 7 6 5 4 3 2 1 ppm
1.575
1.000
1.018
1.220
1.008
1.090
1.044
1.653
3.206
2.393

**$^1$H NMR of 5d**

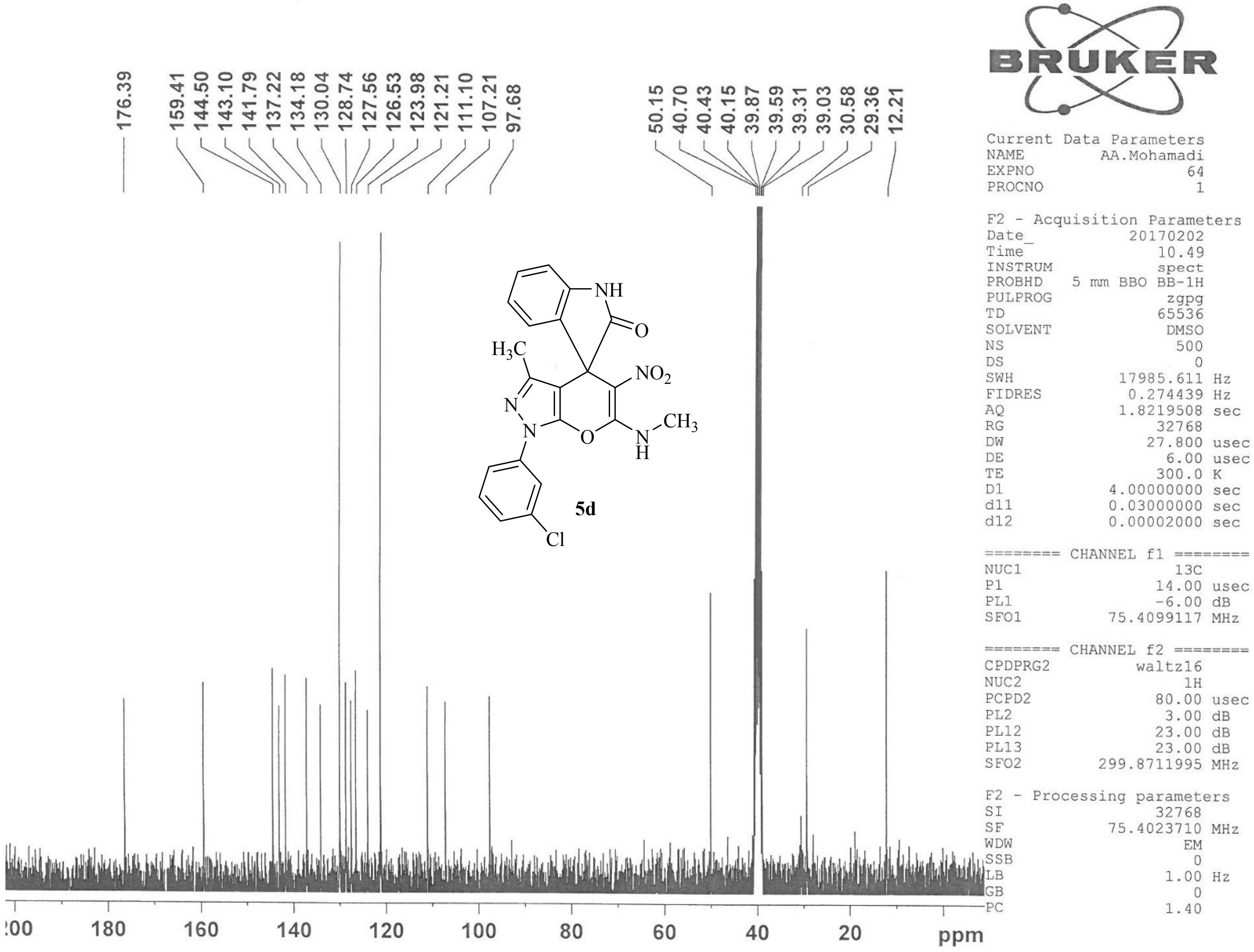
176.39
159.41
144.50
143.10
141.79
137.22
134.18
130.04
128.74
127.56
126.53
123.98
121.21
111.10
107.21
97.68
50.15
40.70
40.43
40.15
39.87
39.59
39.31
39.03
30.58
29.36
12.21
NH
O
$H_3C$
$NO_2$
N
N
O
N
H
$CH_3$
5d
Cl
BRUKER
Current Data Parameters
NAME AA.Mohamadi
EXPNO 64
PROCNO 1
F2 - Acquisition Parameters
Date_ 20170202
Time 10.49
INSTRUM spect
PROBHD 5 mm BBO BB-1H
PULPROG zgpg
TD 65536
SOLVENT DMSO
NS 500
DS 0
SWH 17985.611 Hz
FIDRES 0.274439 Hz
AQ 1.8219508 sec
RG 32768
DW 27.800 usec
DE 6.00 usec
TE 300.0 K
D1 4.00000000 sec
d11 0.03000000 sec
d12 0.00002000 sec
======== CHANNEL f1 ========
NUC1 13C
P1 14.00 usec
PL1 -6.00 dB
SFO1 75.4099117 MHz
======== CHANNEL f2 ========
CPDPRG2 waltz16
NUC2 1H
PCPD2 80.00 usec
PL2 3.00 dB
PL12 23.00 dB
PL13 23.00 dB
SFO2 299.8711995 MHz
F2 - Processing parameters
SI 32768
SF 75.4023710 MHz
WDW EM
SSB 0
LB 1.00 Hz
GB 0
PC 1.40
200
180
160
140
120
100
80
60
40
20
ppm

**$^{13}C$ NMR of 5d**

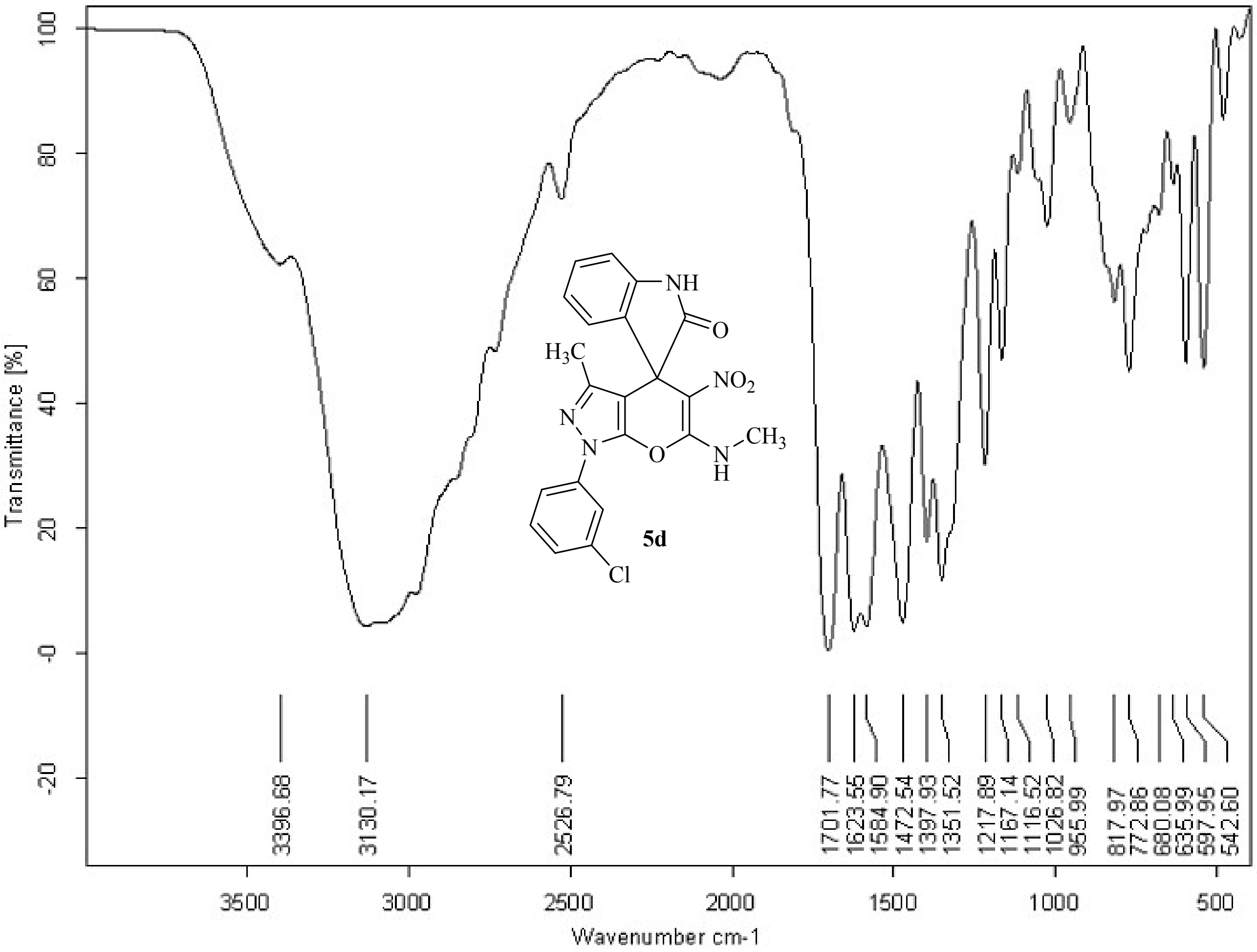
Transmittance [%]
Wavenumber cm-1
100
80
60
40
20
-0
-20
3500
3000
2500
2000
1500
1000
500
3396.68
3130.17
2526.79
1701.77
1623.55
1584.90
1472.54
1397.93
1351.52
1217.89
1167.14
1116.52
1026.82
955.99
817.97
772.86
680.08
635.99
597.95
542.60
NH
O
$H_3C$
$NO_2$
N
N
O
N
H
$CH_3$
Cl
5d

**IR of 5d**

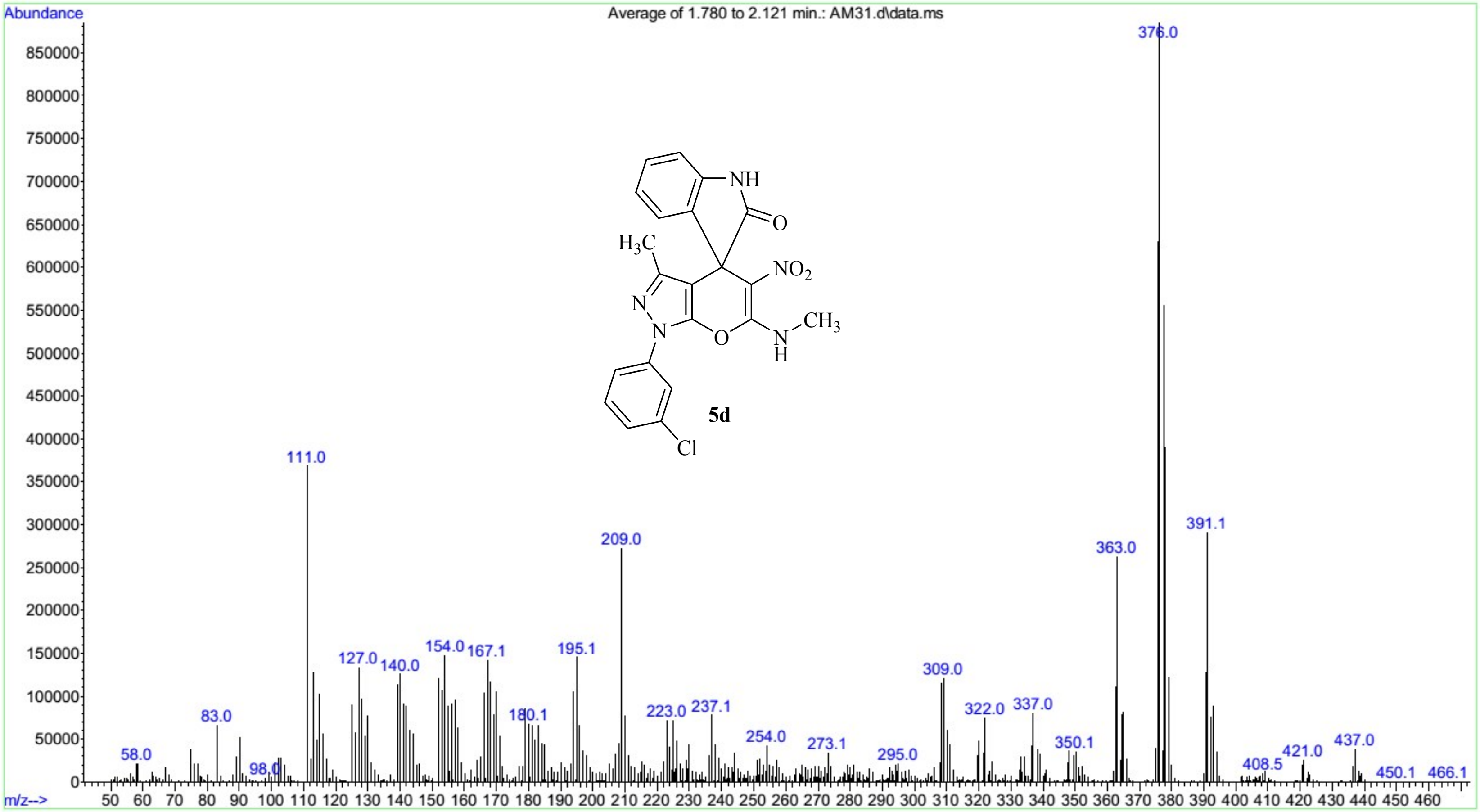
Abundance
Average of 1.780 to 2.121 min.: AM31.d\data.ms
850000
800000
750000
700000
650000
600000
550000
500000
450000
400000
350000
300000
250000
200000
150000
100000
50000
0
m/z-->
376.0
391.1
363.0
209.0
111.0
127.0
140.0
154.0
167.1
195.1
180.1
83.0
58.0
98.0
223.0
237.1
254.0
273.1
295.0
309.0
322.0
337.0
350.1
408.5
421.0
437.0
450.1
466.1
NH
O
$H_3C$
$NO_2$
N
N
O
N
H
$CH_3$
Cl
5d

**Mass of 5d**

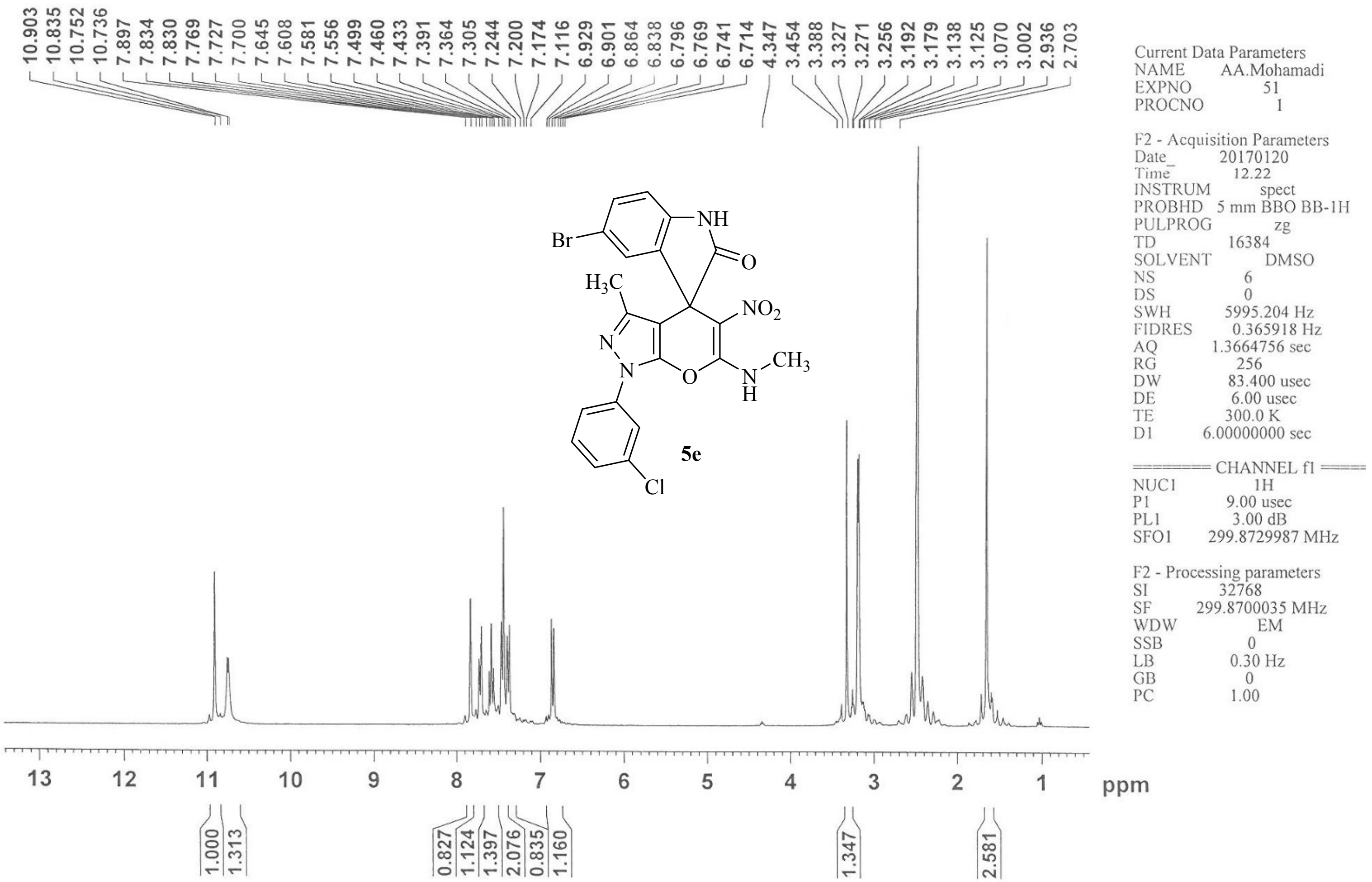


**5e**

**$^{1}$H NMR of 5e**

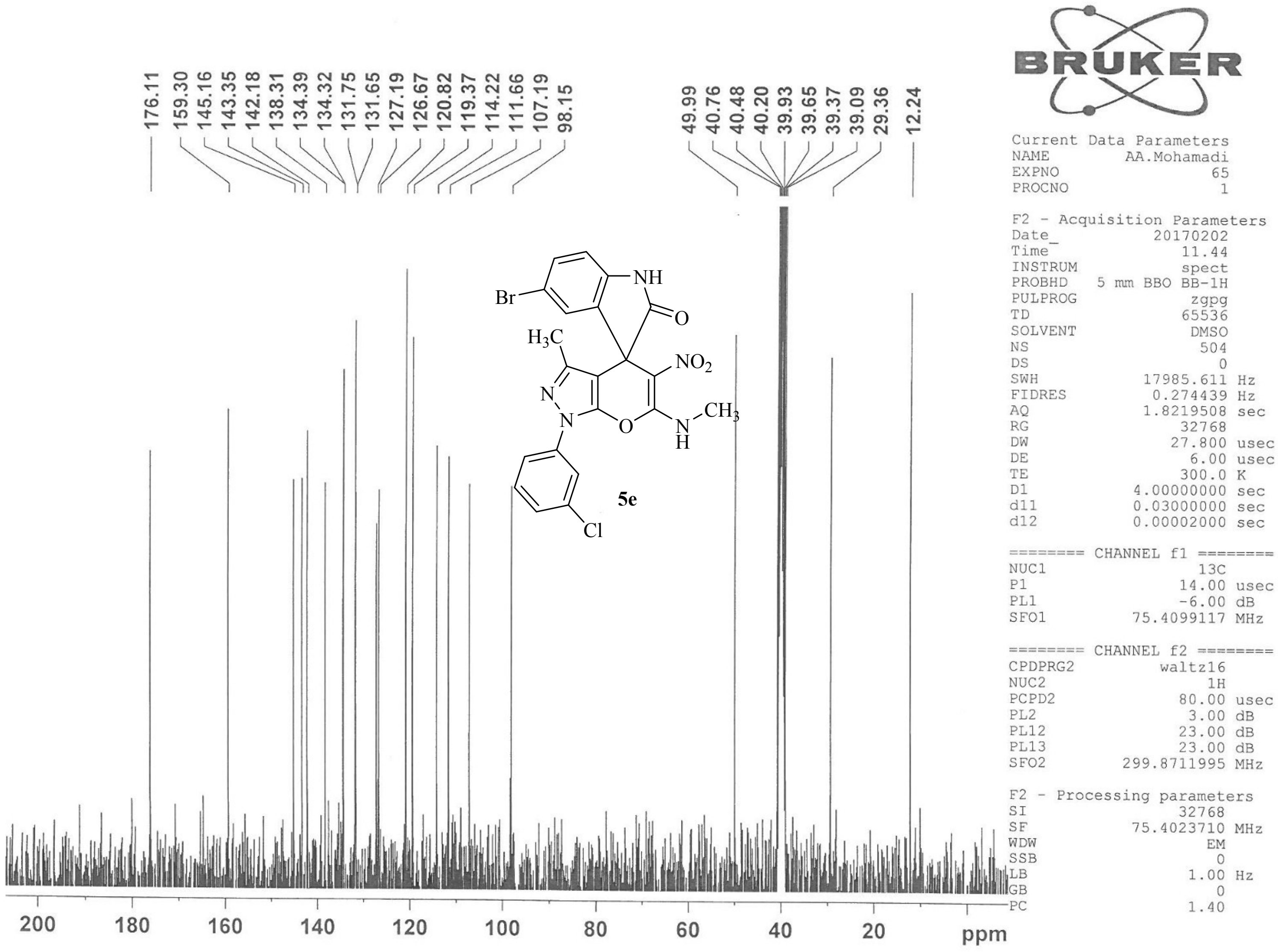

176.11
159.30
145.16
143.35
142.18
138.31
134.39
134.32
131.75
131.65
127.19
126.67
120.82
119.37
114.22
111.66
107.19
98.15
49.99
40.76
40.48
40.20
39.93
39.65
39.37
39.09
29.36
12.24
Br
NH
O
H3C
NO2
N
N
O
N
H
CH3
Cl
5e
Current Data Parameters
NAME AA.Mohamadi
EXPNO 65
PROCNO 1
F2 - Acquisition Parameters
Date_ 20170202
Time 11.44
INSTRUM spect
PROBHD 5 mm BBO BB-1H
PULPROG zgpg
TD 65536
SOLVENT DMSO
NS 504
DS 0
SWH 17985.611 Hz
FIDRES 0.274439 Hz
AQ 1.8219508 sec
RG 32768
DW 27.800 usec
DE 6.00 usec
TE 300.0 K
D1 4.00000000 sec
d11 0.03000000 sec
d12 0.00002000 sec
======== CHANNEL f1 ========
NUC1 13C
P1 14.00 usec
PL1 -6.00 dB
SFO1 75.4099117 MHz
======== CHANNEL f2 ========
CPDPRG2 waltz16
NUC2 1H
PCPD2 80.00 usec
PL2 3.00 dB
PL12 23.00 dB
PL13 23.00 dB
SFO2 299.8711995 MHz
F2 - Processing parameters
SI 32768
SF 75.4023710 MHz
WDW EM
SSB 0
LB 1.00 Hz
GB 0
PC 1.40
200
180
160
140
120
100
80
60
40
20
ppm


**$^{13}$C NMR of 5e**

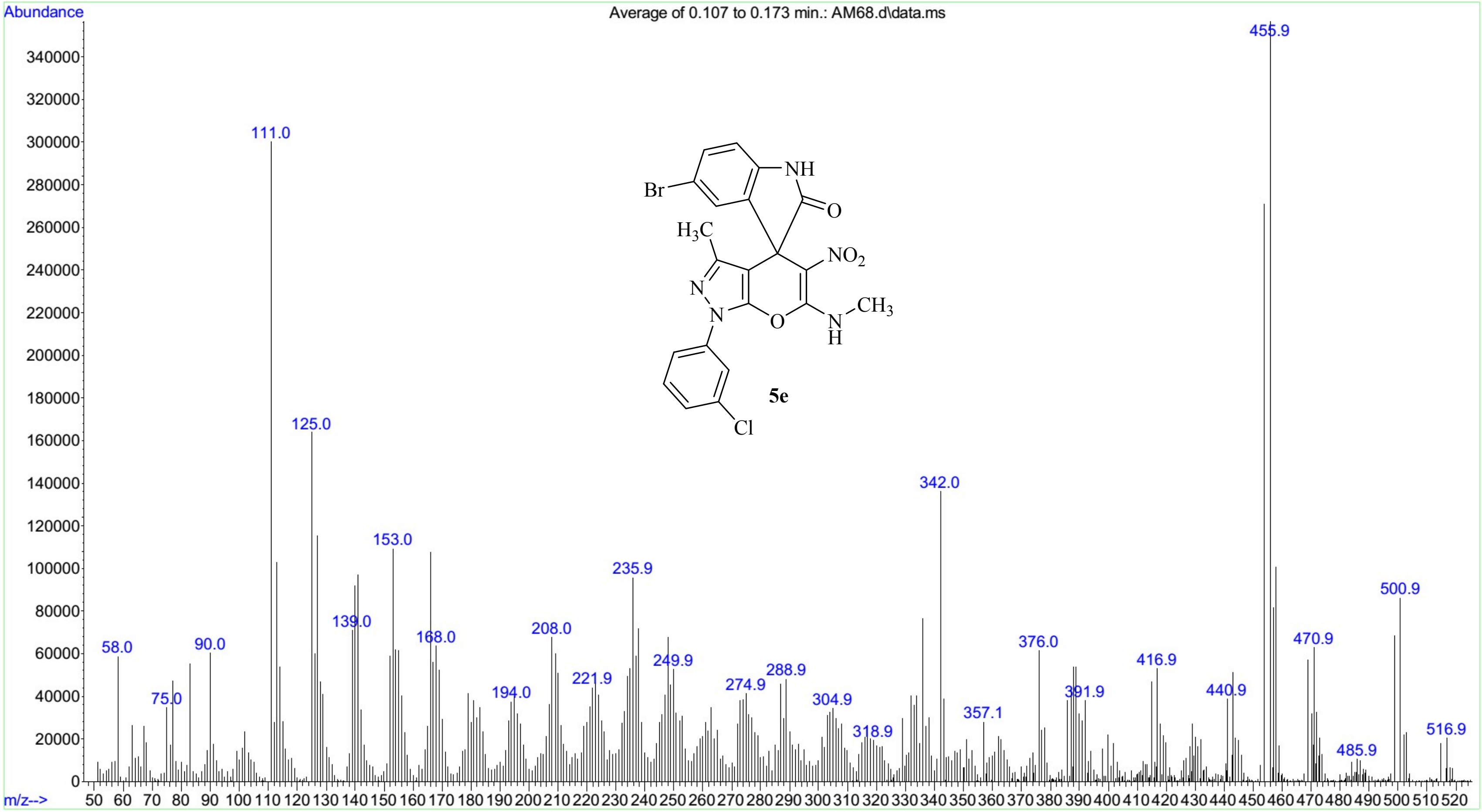
Abundance
Average of 0.107 to 0.173 min.: AM68.d\data.ms
m/z-->
111.0
125.0
153.0
139.0
168.0
208.0
235.9
342.0
455.9
500.9
470.9
416.9
376.0
58.0
75.0
90.0
194.0
221.9
249.9
274.9
288.9
304.9
318.9
357.1
391.9
440.9
485.9
516.9
5e

**Mass of 5e**

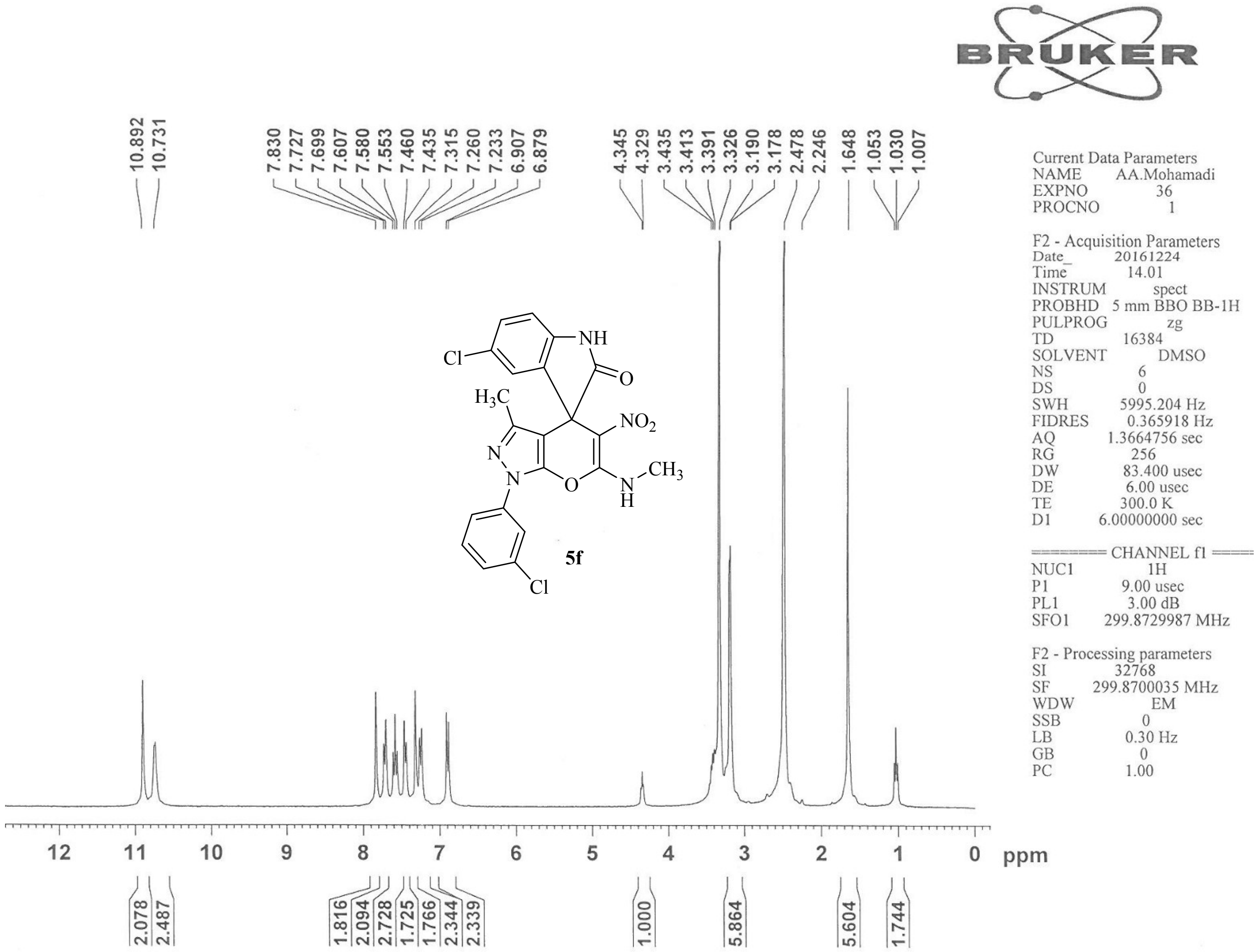
BRUKER
Current Data Parameters
NAME AA.Mohamadi
EXPNO 36
PROCNO 1
F2 - Acquisition Parameters
Date_ 20161224
Time 14.01
INSTRUM spect
PROBHD 5 mm BBO BB-1H
PULPROG zg
TD 16384
SOLVENT DMSO
NS 6
DS 0
SWH 5995.204 Hz
FIDRES 0.365918 Hz
AQ 1.3664756 sec
RG 256
DW 83.400 usec
DE 6.00 usec
TE 300.0 K
D1 6.00000000 sec
CHANNEL f1
NUC1 1H
P1 9.00 usec
PL1 3.00 dB
SFO1 299.8729987 MHz
F2 - Processing parameters
SI 32768
SF 299.8700035 MHz
WDW EM
SSB 0
LB 0.30 Hz
GB 0
PC 1.00
5f
ppm

**$^1$H NMR of 5f**

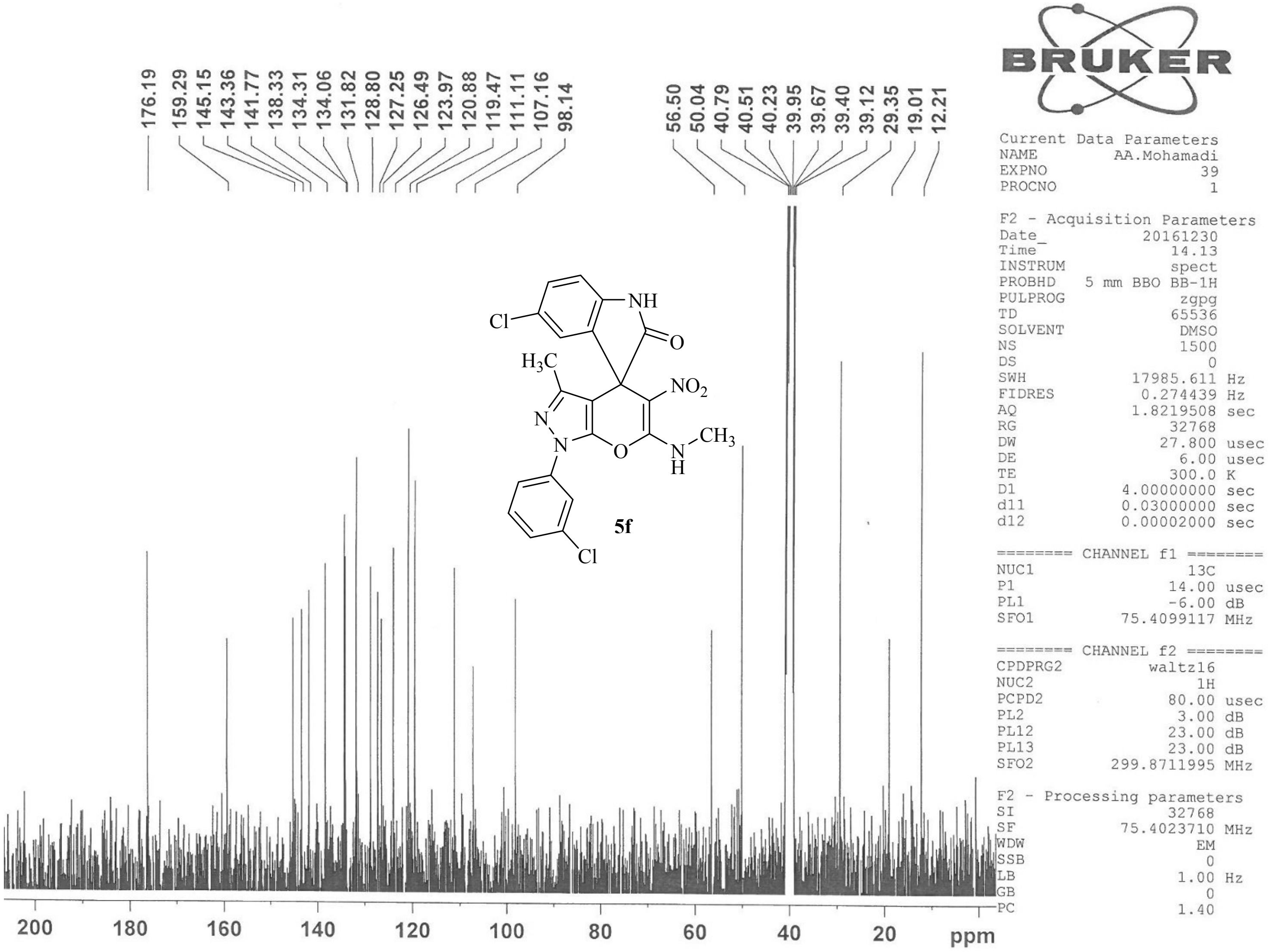
176.19
159.29
145.15
143.36
141.77
138.33
134.31
134.06
131.82
128.80
127.25
126.49
123.97
120.88
119.47
111.11
107.16
98.14
56.50
50.04
40.79
40.51
40.23
39.95
39.67
39.40
39.12
29.35
19.01
12.21
BRUKER
Current Data Parameters
NAME AA.Mohamadi
EXPNO 39
PROCNO 1
F2 - Acquisition Parameters
Date_ 20161230
Time 14.13
INSTRUM spect
PROBHD 5 mm BBO BB-1H
PULPROG zgpg
TD 65536
SOLVENT DMSO
NS 1500
DS 0
SWH 17985.611 Hz
FIDRES 0.274439 Hz
AQ 1.8219508 sec
RG 32768
DW 27.800 usec
DE 6.00 usec
TE 300.0 K
D1 4.00000000 sec
d11 0.03000000 sec
d12 0.00002000 sec
======== CHANNEL f1 ========
NUC1 13C
P1 14.00 usec
PL1 -6.00 dB
SFO1 75.4099117 MHz
======== CHANNEL f2 ========
CPDPRG2 waltz16
NUC2 1H
PCPD2 80.00 usec
PL2 3.00 dB
PL12 23.00 dB
PL13 23.00 dB
SFO2 299.8711995 MHz
F2 - Processing parameters
SI 32768
SF 75.4023710 MHz
WDW EM
SSB 0
LB 1.00 Hz
GB 0
PC 1.40
Cl
NH
O
H3C
NO2
N
N
O
N
H
CH3
Cl
5f
200
180
160
140
120
100
80
60
40
20
ppm

**$^{13}$C NMR of 5f**

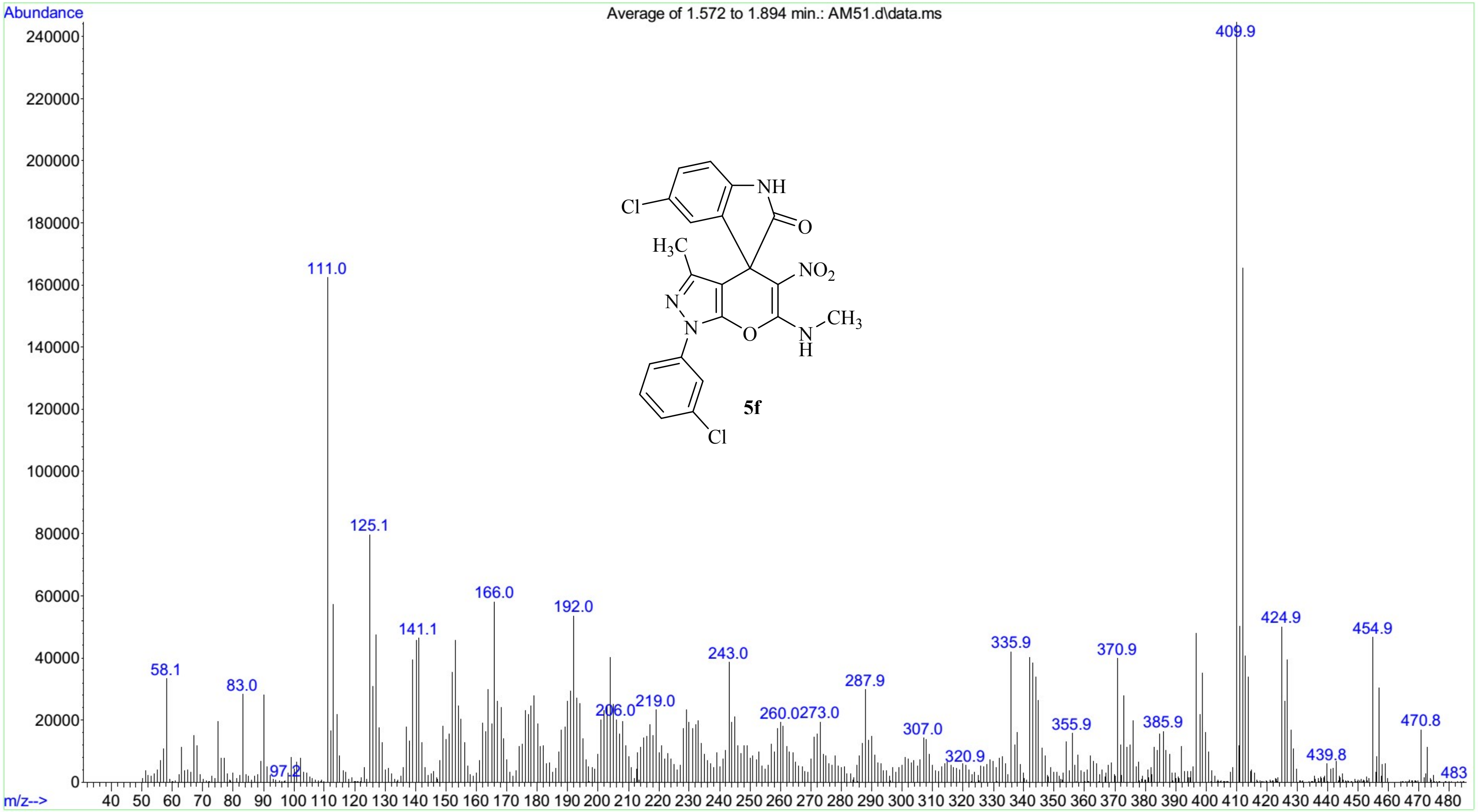

Abundance
Average of 1.572 to 1.894 min.: AM51.d\data.ms
240000
220000
200000
180000
160000
140000
120000
100000
80000
60000
40000
20000
0
m/z-->
40 50 60 70 80 90 100 110 120 130 140 150 160 170 180 190 200 210 220 230 240 250 260 270 280 290 300 310 320 330 340 350 360 370 380 390 400 410 420 430 440 450 460 470 480
409.9
111.0
125.1
166.0
192.0
141.1
58.1
83.0
97.2
206.0
219.0
243.0
260.0
273.0
287.9
307.0
320.9
335.9
355.9
370.9
385.9
424.9
439.8
454.9
470.8
483
Cl
NH
O
$H_3C$
$NO_2$
N
N
O
N
H
$CH_3$
Cl
5f


**Mass of 5f**

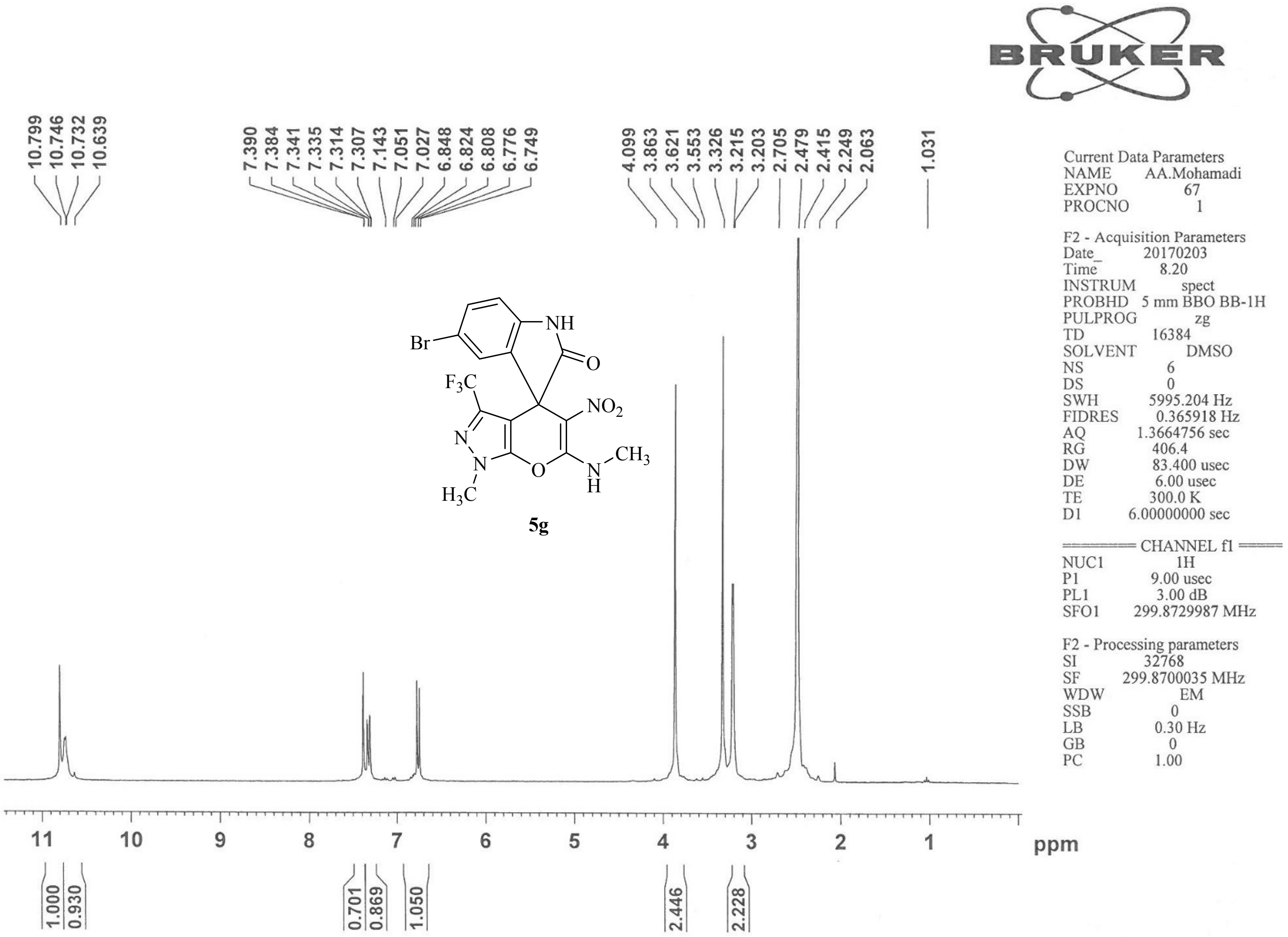
10.799
10.746
10.732
10.639
7.390
7.384
7.341
7.335
7.314
7.307
7.143
7.051
7.027
6.848
6.824
6.808
6.776
6.749
4.099
3.863
3.621
3.553
3.326
3.215
3.203
2.705
2.479
2.415
2.249
2.063
1.031
Br
NH
O
F3C
NO2
N
N
O
N
H
CH3
H3C
5g
Current Data Parameters
NAME AA.Mohamadi
EXPNO 67
PROCNO 1
F2 - Acquisition Parameters
Date_ 20170203
Time 8.20
INSTRUM spect
PROBHD 5 mm BBO BB-1H
PULPROG zg
TD 16384
SOLVENT DMSO
NS 6
DS 0
SWH 5995.204 Hz
FIDRES 0.365918 Hz
AQ 1.3664756 sec
RG 406.4
DW 83.400 usec
DE 6.00 usec
TE 300.0 K
D1 6.00000000 sec
CHANNEL f1
NUC1 1H
P1 9.00 usec
PL1 3.00 dB
SFO1 299.8729987 MHz
F2 - Processing parameters
SI 32768
SF 299.8700035 MHz
WDW EM
SSB 0
LB 0.30 Hz
GB 0
PC 1.00
11
10
9
8
7
6
5
4
3
2
1
ppm
1.000
0.930
0.701
0.869
1.050
2.446
2.228

**$^1$H NMR of 5g**

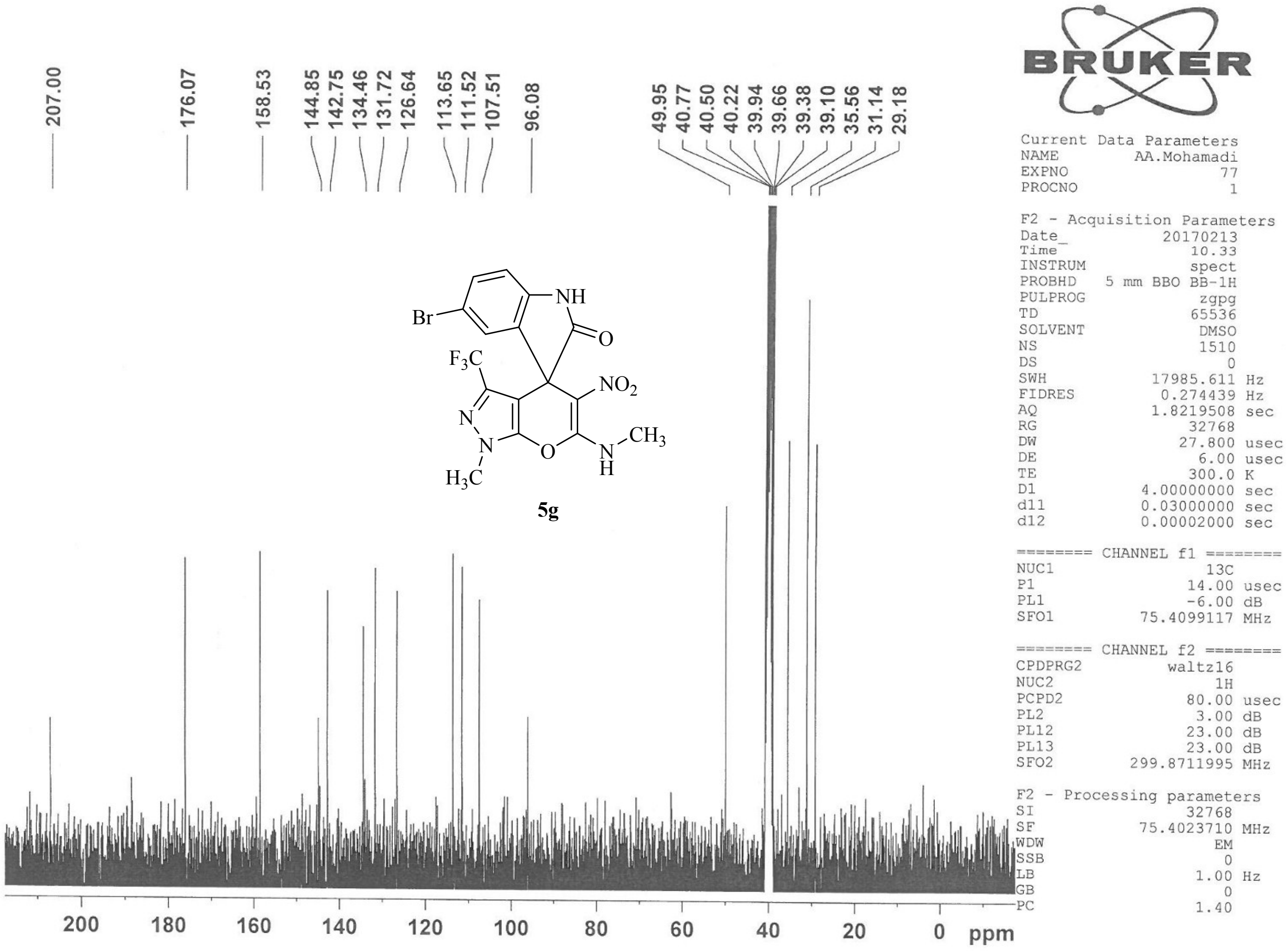
207.00
176.07
158.53
144.85
142.75
134.46
131.72
126.64
113.65
111.52
107.51
96.08
49.95
40.77
40.50
40.22
39.94
39.66
39.38
39.10
35.56
31.14
29.18
Br
NH
O
$F_3C$
$NO_2$
N
N
O
$CH_3$
H
$H_3C$
5g
200 180 160 140 120 100 80 60 40 20 0 ppm
BRUKER
Current Data Parameters
NAME AA.Mohamadi
EXPNO 77
PROCNO 1
F2 - Acquisition Parameters
Date_ 20170213
Time 10.33
INSTRUM spect
PROBHD 5 mm BBO BB-1H
PULPROG zgpg
TD 65536
SOLVENT DMSO
NS 1510
DS 0
SWH 17985.611 Hz
FIDRES 0.274439 Hz
AQ 1.8219508 sec
RG 32768
DW 27.800 usec
DE 6.00 usec
TE 300.0 K
D1 4.00000000 sec
d11 0.03000000 sec
d12 0.00002000 sec
======== CHANNEL f1 ========
NUC1 13C
P1 14.00 usec
PL1 -6.00 dB
SFO1 75.4099117 MHz
======== CHANNEL f2 ========
CPDPRG2 waltz16
NUC2 1H
PCPD2 80.00 usec
PL2 3.00 dB
PL12 23.00 dB
PL13 23.00 dB
SFO2 299.8711995 MHz
F2 - Processing parameters
SI 32768
SF 75.4023710 MHz
WDW EM
SSB 0
LB 1.00 Hz
GB 0
PC 1.40

**$^{13}C$ NMR of 5g**

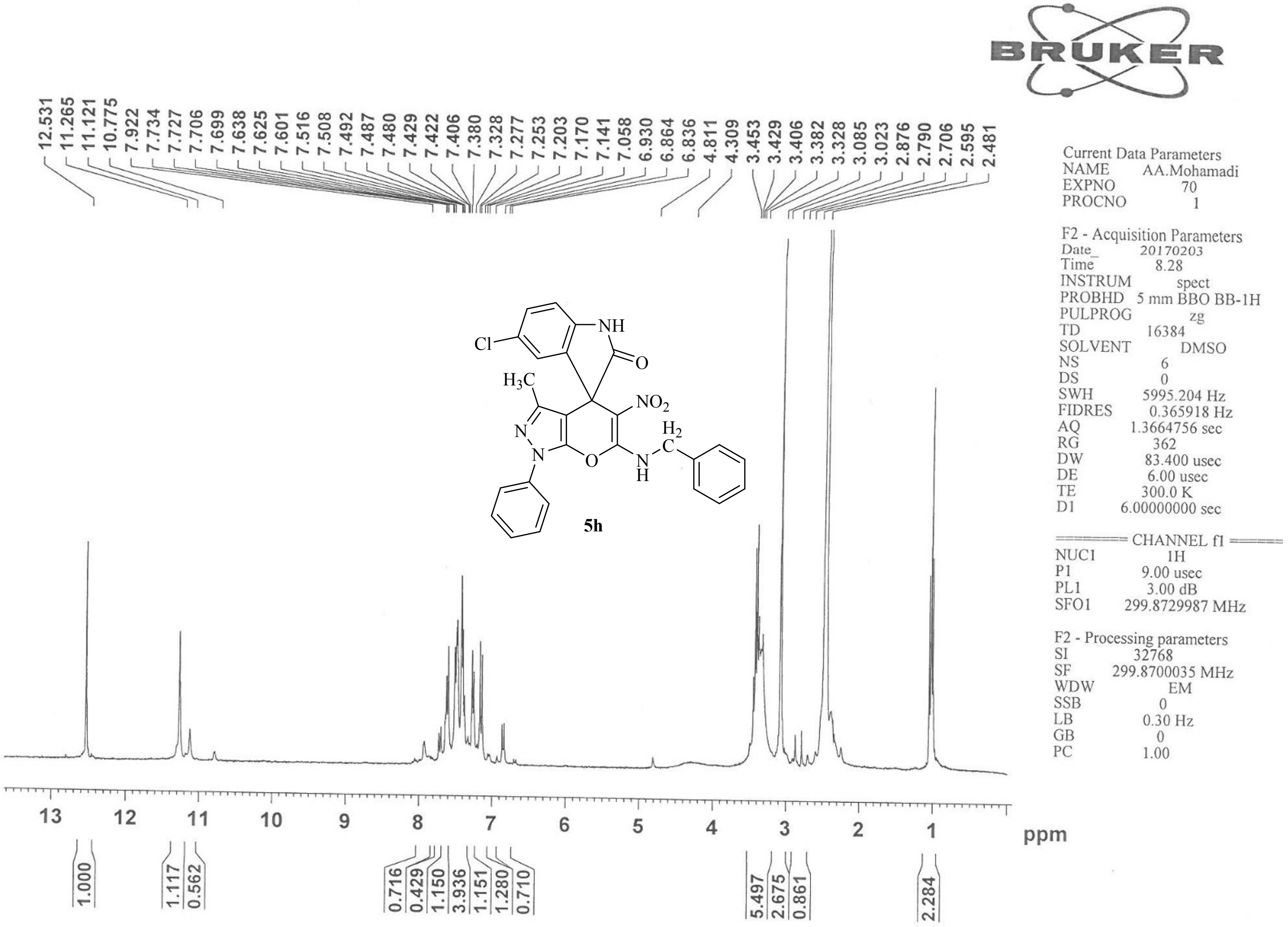

12.531
11.265
11.121
10.775
7.922
7.734
7.727
7.706
7.699
7.638
7.625
7.601
7.516
7.508
7.492
7.487
7.480
7.429
7.422
7.406
7.380
7.328
7.277
7.253
7.203
7.170
7.141
7.058
6.930
6.864
6.836
4.811
4.309
3.453
3.429
3.406
3.382
3.328
3.085
3.023
2.876
2.790
2.706
2.595
2.481
BRUKER
Current Data Parameters
NAME AA.Mohamadi
EXPNO 70
PROCNO 1
F2 - Acquisition Parameters
Date_ 20170203
Time 8.28
INSTRUM spect
PROBHD 5 mm BBO BB-1H
PULPROG zg
TD 16384
SOLVENT DMSO
NS 6
DS 0
SWH 5995.204 Hz
FIDRES 0.365918 Hz
AQ 1.3664756 sec
RG 362
DW 83.400 usec
DE 6.00 usec
TE 300.0 K
D1 6.00000000 sec
CHANNEL f1
NUC1 1H
P1 9.00 usec
PL1 3.00 dB
SFO1 299.8729987 MHz
F2 - Processing parameters
SI 32768
SF 299.8700035 MHz
WDW EM
SSB 0
LB 0.30 Hz
GB 0
PC 1.00
Cl
NH
O
H3C
NO2
N
N
O
N
H
H2
C
5h
13
12
11
10
9
8
7
6
5
4
3
2
1
ppm
1.000
1.117
0.562
0.716
0.429
1.150
3.936
1.151
1.280
0.710
5.497
2.675
0.861
2.284


**$^1$H NMR of 5h**

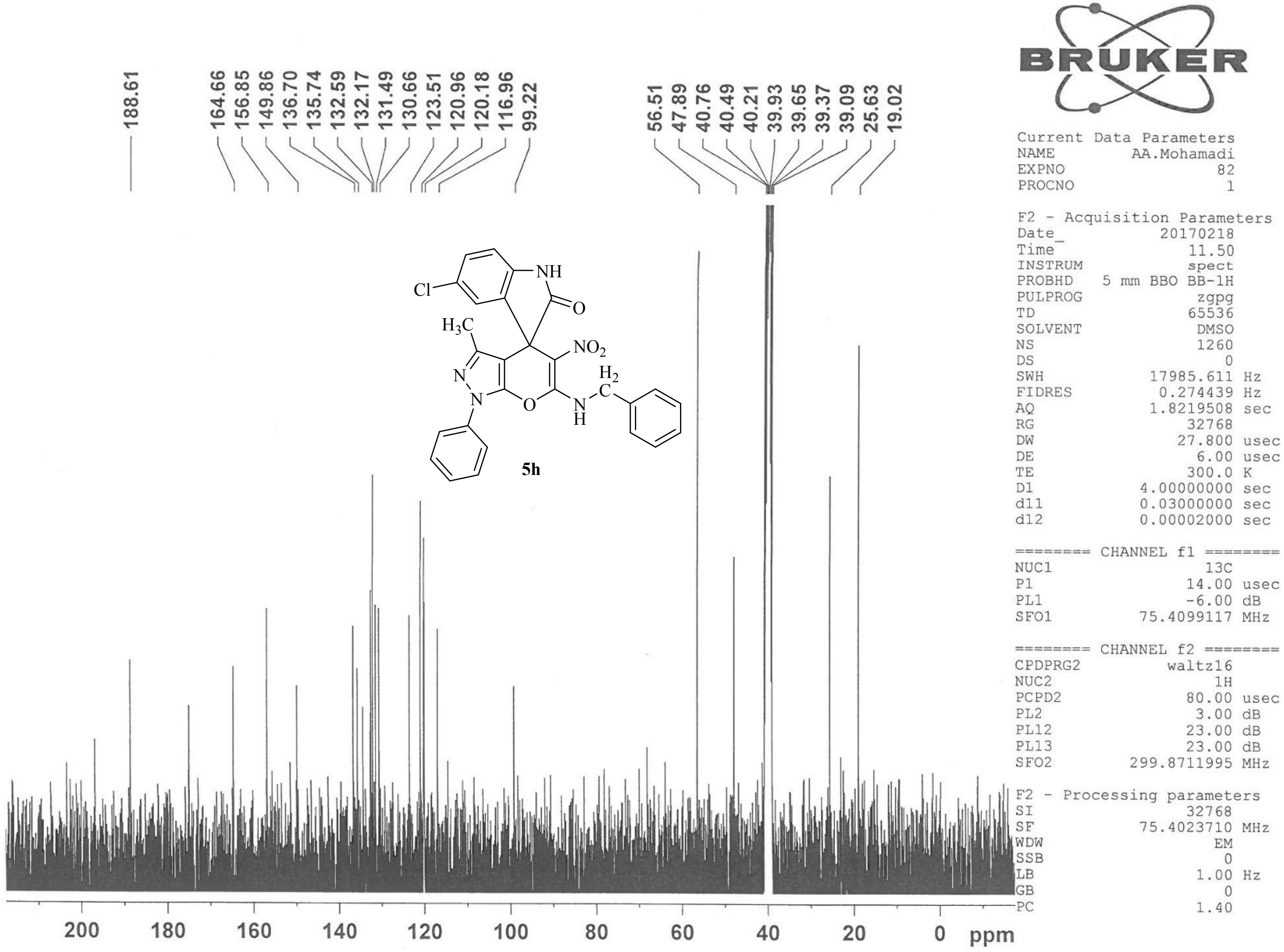

188.61
164.66
156.85
149.86
136.70
135.74
132.59
132.17
131.49
130.66
123.51
120.96
120.18
116.96
99.22
56.51
47.89
40.76
40.49
40.21
39.93
39.65
39.37
39.09
25.63
19.02
Cl
NH
O
H3C
NO2
H2
C
N
N
O
N
H
5h
200 180 160 140 120 100 80 60 40 20 0 ppm
Current Data Parameters
NAME AA.Mohamadi
EXPNO 82
PROCNO 1
F2 - Acquisition Parameters
Date_ 20170218
Time 11.50
INSTRUM spect
PROBHD 5 mm BBO BB-1H
PULPROG zgpg
TD 65536
SOLVENT DMSO
NS 1260
DS 0
SWH 17985.611 Hz
FIDRES 0.274439 Hz
AQ 1.8219508 sec
RG 32768
DW 27.800 usec
DE 6.00 usec
TE 300.0 K
D1 4.00000000 sec
d11 0.03000000 sec
d12 0.00002000 sec
======== CHANNEL f1 ========
NUC1 13C
P1 14.00 usec
PL1 -6.00 dB
SFO1 75.4099117 MHz
======== CHANNEL f2 ========
CPDPRG2 waltz16
NUC2 1H
PCPD2 80.00 usec
PL2 3.00 dB
PL12 23.00 dB
PL13 23.00 dB
SFO2 299.8711995 MHz
F2 - Processing parameters
SI 32768
SF 75.4023710 MHz
WDW EM
SSB 0
LB 1.00 Hz
GB 0
PC 1.40


**$^{13}$C NMR of 5h**

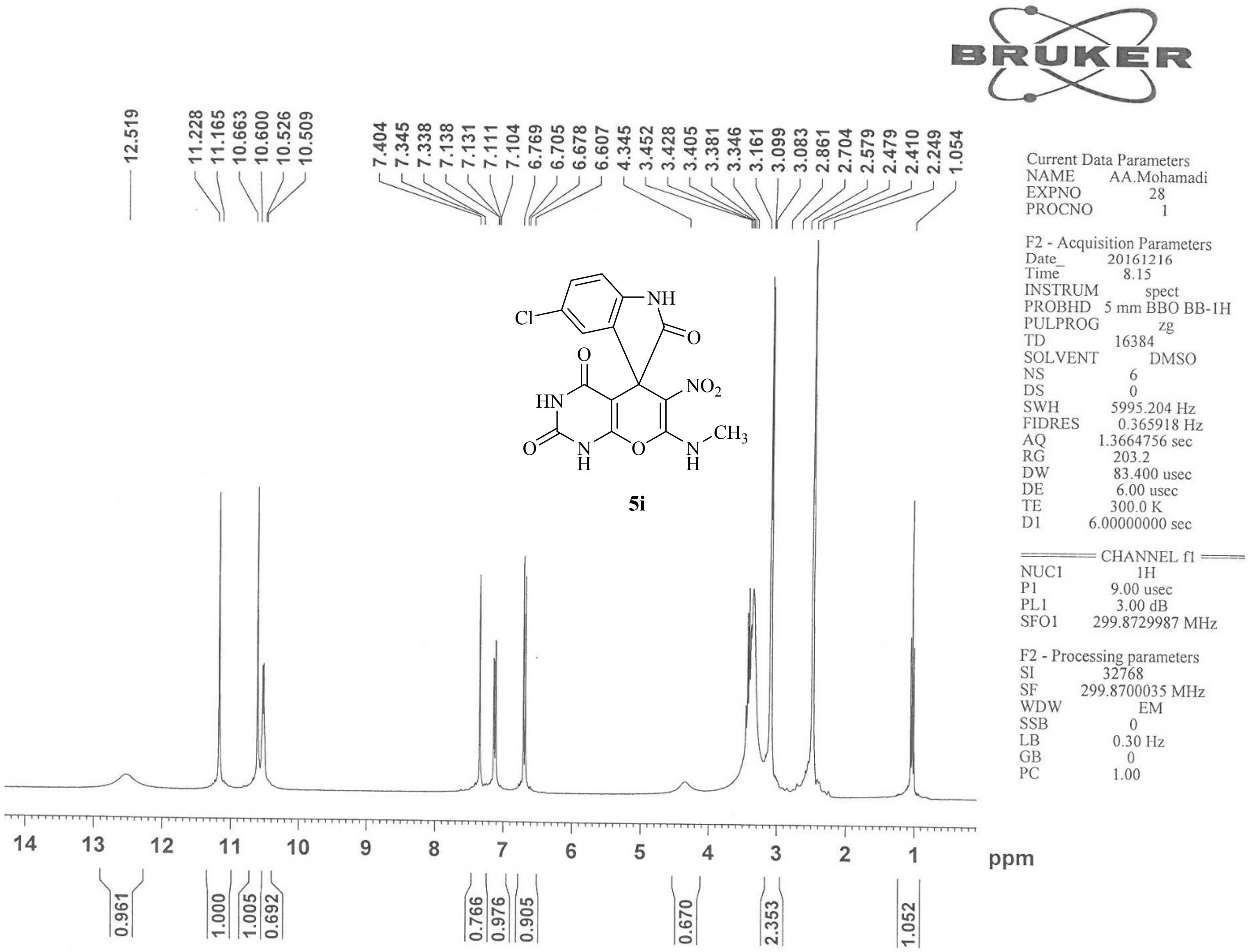
BRUKER
12.519
11.228
11.165
10.663
10.600
10.526
10.509
7.404
7.345
7.338
7.138
7.131
7.111
7.104
6.769
6.705
6.678
6.607
4.345
3.452
3.428
3.405
3.381
3.346
3.161
3.099
3.083
2.861
2.704
2.579
2.479
2.410
2.249
1.054
Cl
NH
O
O
HN
NO2
O
N
H
O
N
H
CH3
5i
Current Data Parameters
NAME AA.Mohamadi
EXPNO 28
PROCNO 1
F2 - Acquisition Parameters
Date_ 20161216
Time 8.15
INSTRUM spect
PROBHD 5 mm BBO BB-1H
PULPROG zg
TD 16384
SOLVENT DMSO
NS 6
DS 0
SWH 5995.204 Hz
FIDRES 0.365918 Hz
AQ 1.3664756 sec
RG 203.2
DW 83.400 usec
DE 6.00 usec
TE 300.0 K
D1 6.00000000 sec
CHANNEL f1
NUC1 1H
P1 9.00 usec
PL1 3.00 dB
SFO1 299.8729987 MHz
F2 - Processing parameters
SI 32768
SF 299.8700035 MHz
WDW EM
SSB 0
LB 0.30 Hz
GB 0
PC 1.00
14 13 12 11 10 9 8 7 6 5 4 3 2 1 ppm
0.961
1.000
1.005
0.692
0.766
0.976
0.905
0.670
2.353
1.052

**$^1$H NMR of 5i**

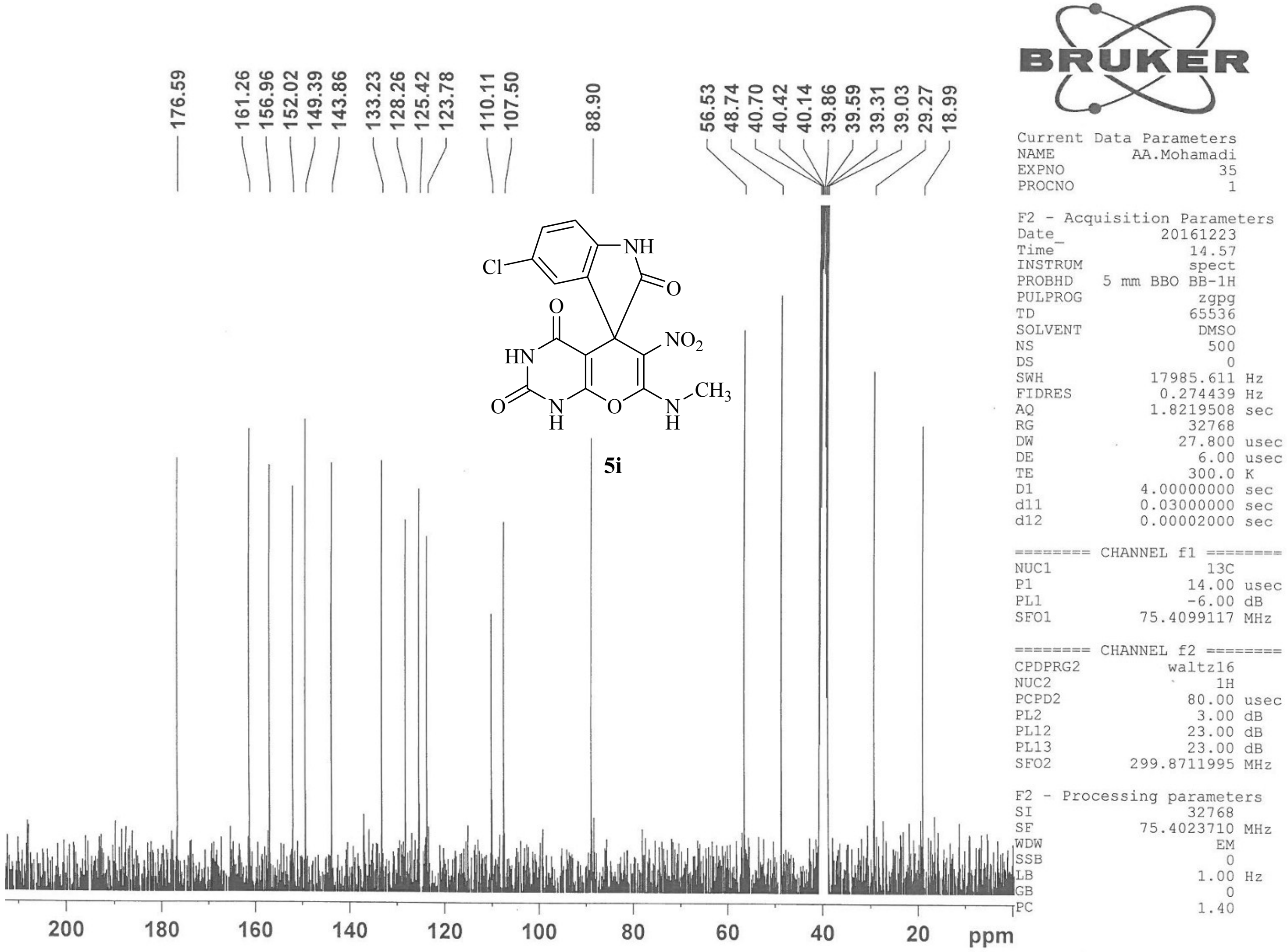
176.59
161.26
156.96
152.02
149.39
143.86
133.23
128.26
125.42
123.78
110.11
107.50
88.90
56.53
48.74
40.70
40.42
40.14
39.86
39.59
39.31
39.03
29.27
18.99
Cl
NH
O
O
HN
NO2
O
N
H
O
N
H
CH3
5i
200
180
160
140
120
100
80
60
40
20
ppm
BRUKER
Current Data Parameters
NAME AA.Mohamadi
EXPNO 35
PROCNO 1
F2 - Acquisition Parameters
Date_ 20161223
Time 14.57
INSTRUM spect
PROBHD 5 mm BBO BB-1H
PULPROG zgpg
TD 65536
SOLVENT DMSO
NS 500
DS 0
SWH 17985.611 Hz
FIDRES 0.274439 Hz
AQ 1.8219508 sec
RG 32768
DW 27.800 usec
DE 6.00 usec
TE 300.0 K
D1 4.00000000 sec
d11 0.03000000 sec
d12 0.00002000 sec
======== CHANNEL f1 ========
NUC1 13C
P1 14.00 usec
PL1 -6.00 dB
SFO1 75.4099117 MHz
======== CHANNEL f2 ========
CPDPRG2 waltz16
NUC2 1H
PCPD2 80.00 usec
PL2 3.00 dB
PL12 23.00 dB
PL13 23.00 dB
SFO2 299.8711995 MHz
F2 - Processing parameters
SI 32768
SF 75.4023710 MHz
WDW EM
SSB 0
LB 1.00 Hz
GB 0
PC 1.40

**$^{13}C$ NMR of 5i**

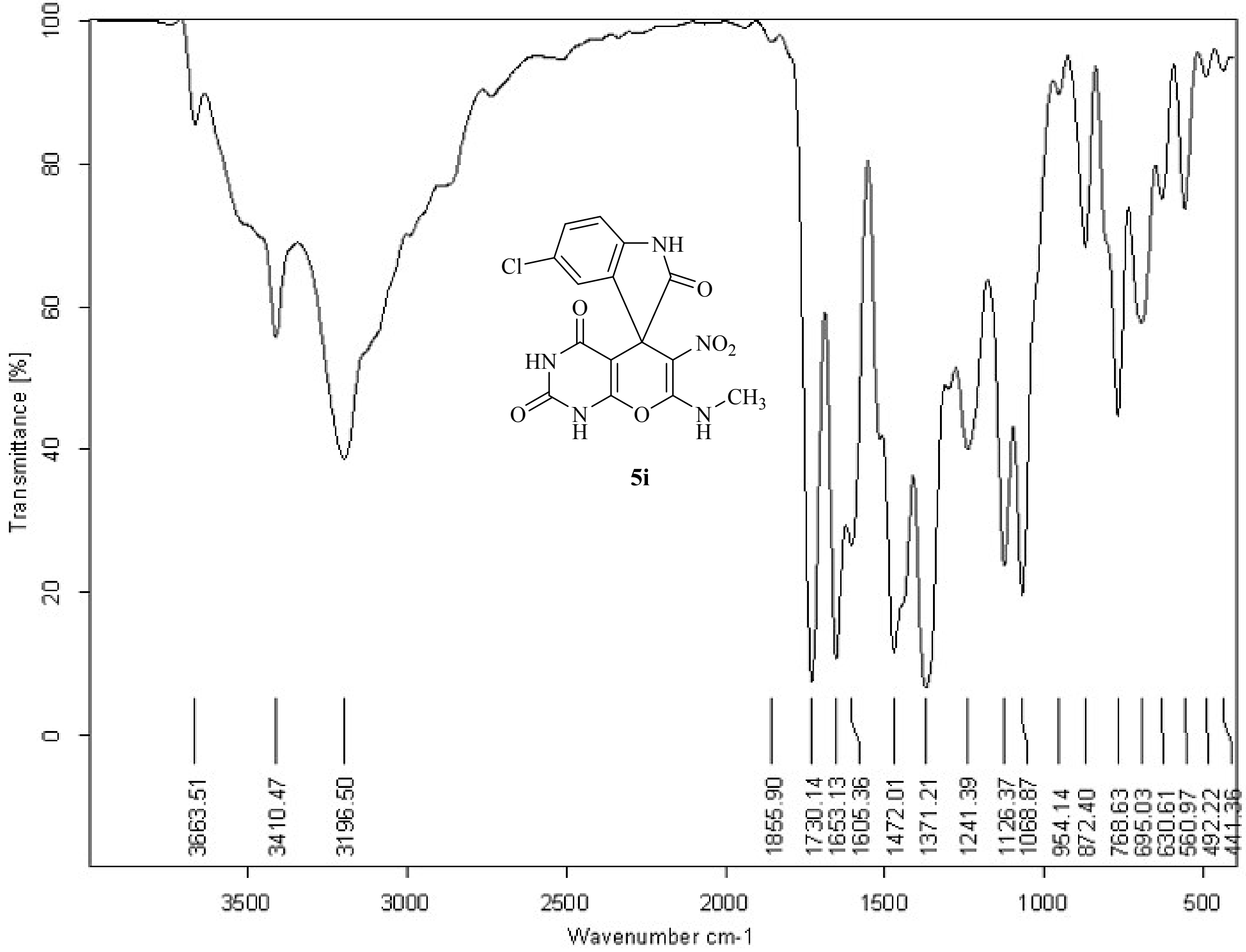
Transmittance [%]
Wavenumber cm-1
3663.51
3410.47
3196.50
1855.90
1730.14
1653.13
1605.36
1472.01
1371.21
1241.39
1126.37
1068.87
954.14
872.40
768.63
695.03
630.61
560.97
492.22
441.36
Cl
NH
O
O
NO2
HN
O
N
H
O
N
H
CH3
5i

**IR of 5i**

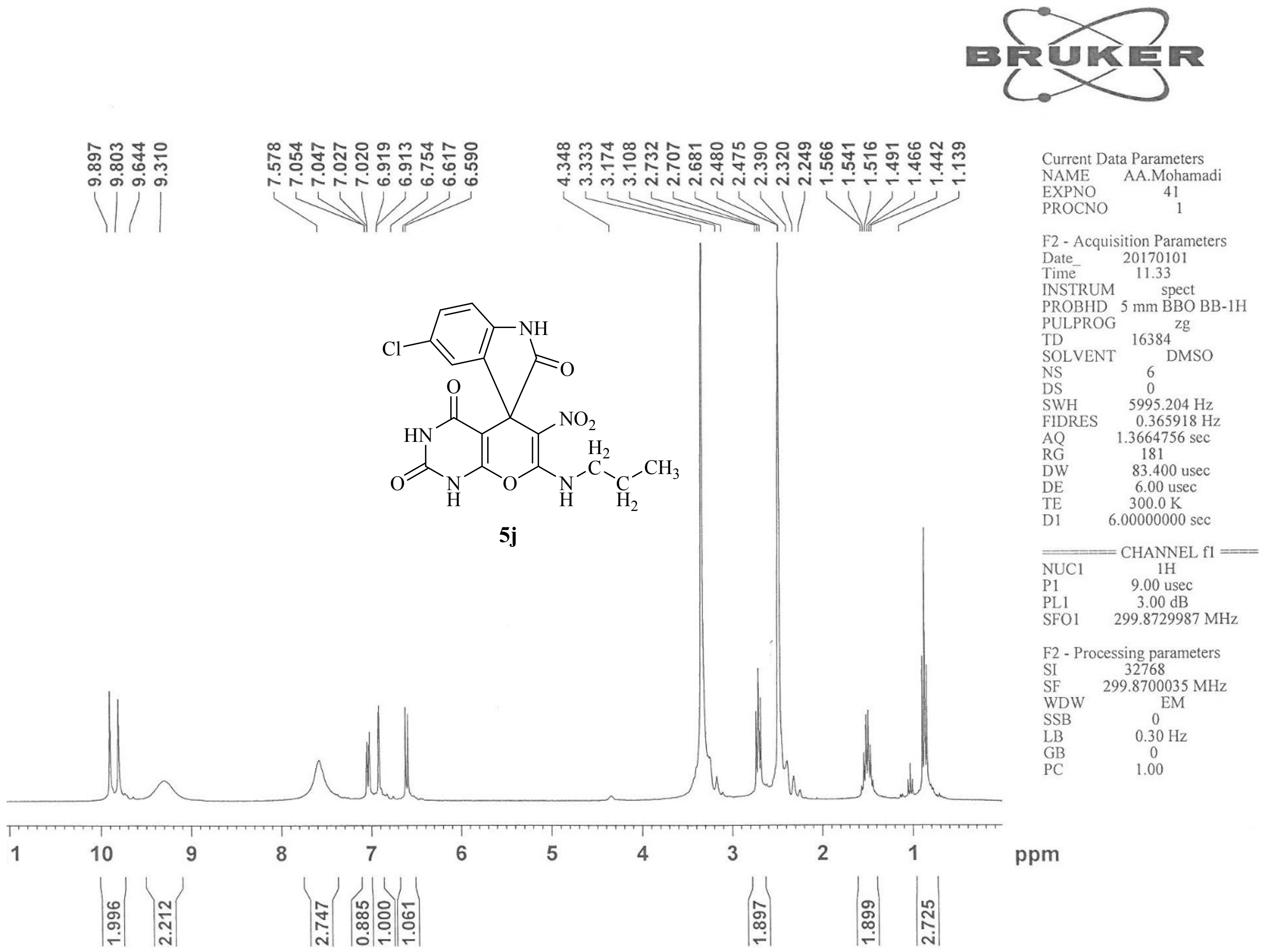
BRUKER
9.897
9.803
9.644
9.310
7.578
7.054
7.047
7.027
7.020
6.919
6.913
6.754
6.617
6.590
4.348
3.333
3.174
3.108
2.732
2.707
2.681
2.480
2.475
2.390
2.320
2.249
1.566
1.541
1.516
1.491
1.466
1.442
1.139
Cl
NH
O
O
$NO_2$
HN
$H_2$
$CH_3$
O
N
H
O
N
H
$H_2$
5j
Current Data Parameters
NAME AA.Mohamadi
EXPNO 41
PROCNO 1
F2 - Acquisition Parameters
Date_ 20170101
Time 11.33
INSTRUM spect
PROBHD 5 mm BBO BB-1H
PULPROG zg
TD 16384
SOLVENT DMSO
NS 6
DS 0
SWH 5995.204 Hz
FIDRES 0.365918 Hz
AQ 1.3664756 sec
RG 181
DW 83.400 usec
DE 6.00 usec
TE 300.0 K
D1 6.00000000 sec
CHANNEL f1
NUC1 1H
P1 9.00 usec
PL1 3.00 dB
SFO1 299.8729987 MHz
F2 - Processing parameters
SI 32768
SF 299.8700035 MHz
WDW EM
SSB 0
LB 0.30 Hz
GB 0
PC 1.00
1
10
9
8
7
6
5
4
3
2
1
ppm
1.996
2.212
2.747
0.885
1.000
1.061
1.897
1.899
2.725

**$^1$H NMR of 5j**

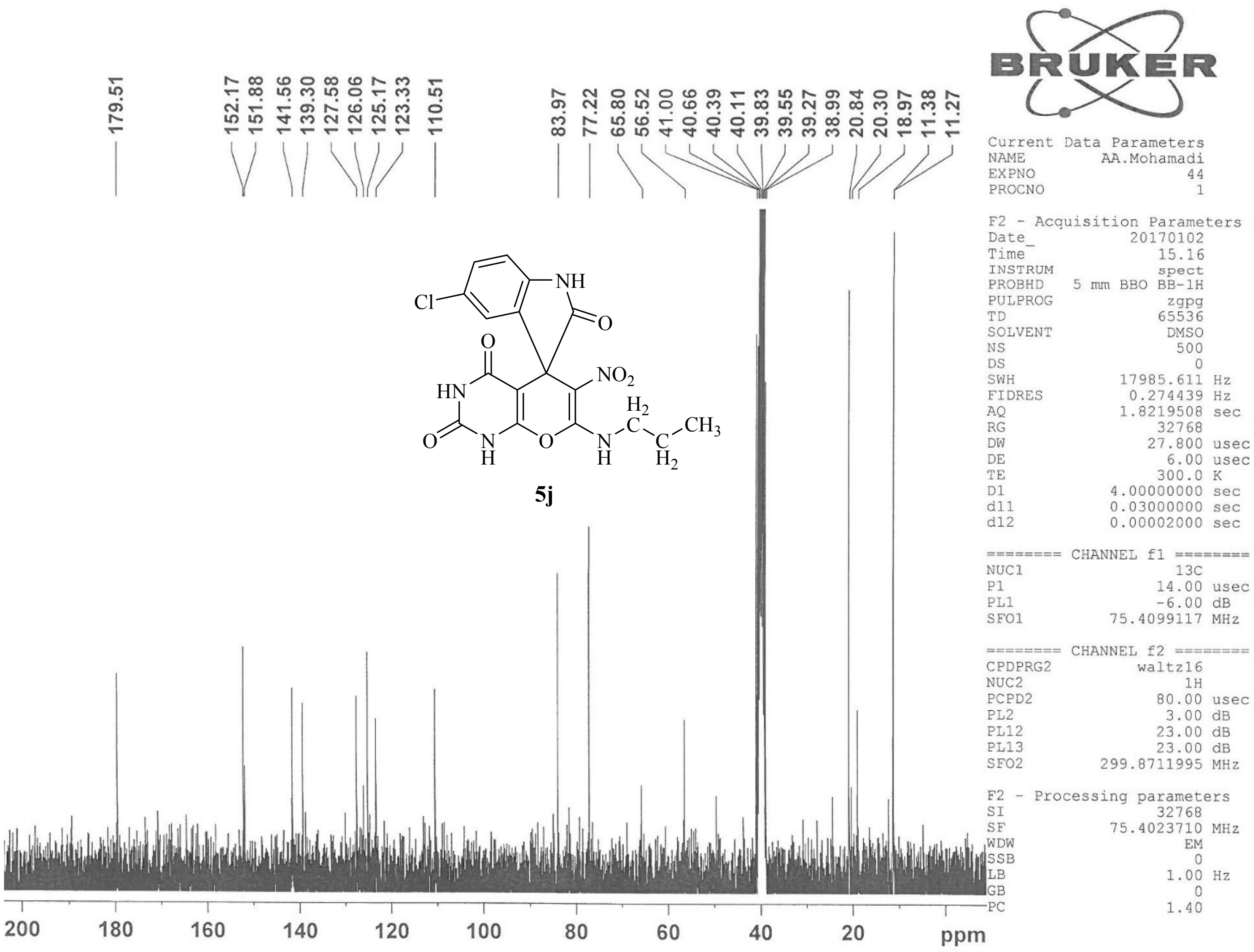
179.51
152.17
151.88
141.56
139.30
127.58
126.06
125.17
123.33
110.51
83.97
77.22
65.80
56.52
41.00
40.66
40.39
40.11
39.83
39.55
39.27
38.99
20.84
20.30
18.97
11.38
11.27
BRUKER
Current Data Parameters
NAME AA.Mohamadi
EXPNO 44
PROCNO 1
F2 - Acquisition Parameters
Date_ 20170102
Time 15.16
INSTRUM spect
PROBHD 5 mm BBO BB-1H
PULPROG zgpg
TD 65536
SOLVENT DMSO
NS 500
DS 0
SWH 17985.611 Hz
FIDRES 0.274439 Hz
AQ 1.8219508 sec
RG 32768
DW 27.800 usec
DE 6.00 usec
TE 300.0 K
D1 4.00000000 sec
d11 0.03000000 sec
d12 0.00002000 sec
======== CHANNEL f1 ========
NUC1 13C
P1 14.00 usec
PL1 -6.00 dB
SFO1 75.4099117 MHz
======== CHANNEL f2 ========
CPDPRG2 waltz16
NUC2 1H
PCPD2 80.00 usec
PL2 3.00 dB
PL12 23.00 dB
PL13 23.00 dB
SFO2 299.8711995 MHz
F2 - Processing parameters
SI 32768
SF 75.4023710 MHz
WDW EM
SSB 0
LB 1.00 Hz
GB 0
PC 1.40
200 180 160 140 120 100 80 60 40 20 ppm
Cl
NH
O
O
NO2
HN
H2
CH3
O
N
H
O
N
H
H2
5j

**$^{13}C$ NMR of 5j**

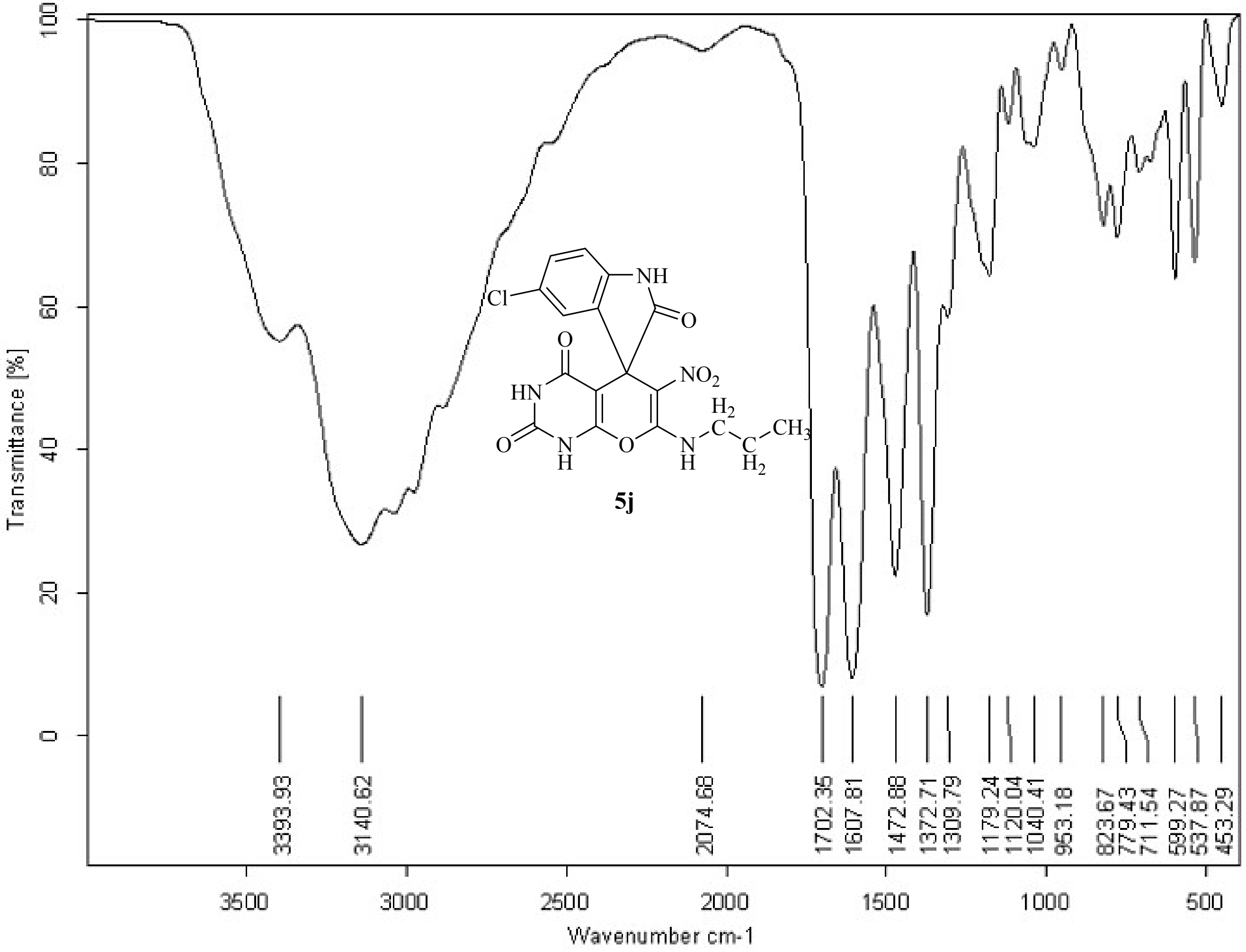
Transmittance [%]
100
80
60
40
20
0
3393.93
3140.62
2074.68
1702.35
1607.81
1472.88
1372.71
1309.79
1179.24
1120.04
1040.41
953.18
823.67
779.43
711.54
599.27
537.87
453.29
3500
3000
2500
2000
1500
1000
500
Wavenumber cm-1
Cl
NH
O
O
NO2
HN
H2
O
N
O
N
C
CH3
H
H
C
H2
5j

**IR of 5j**

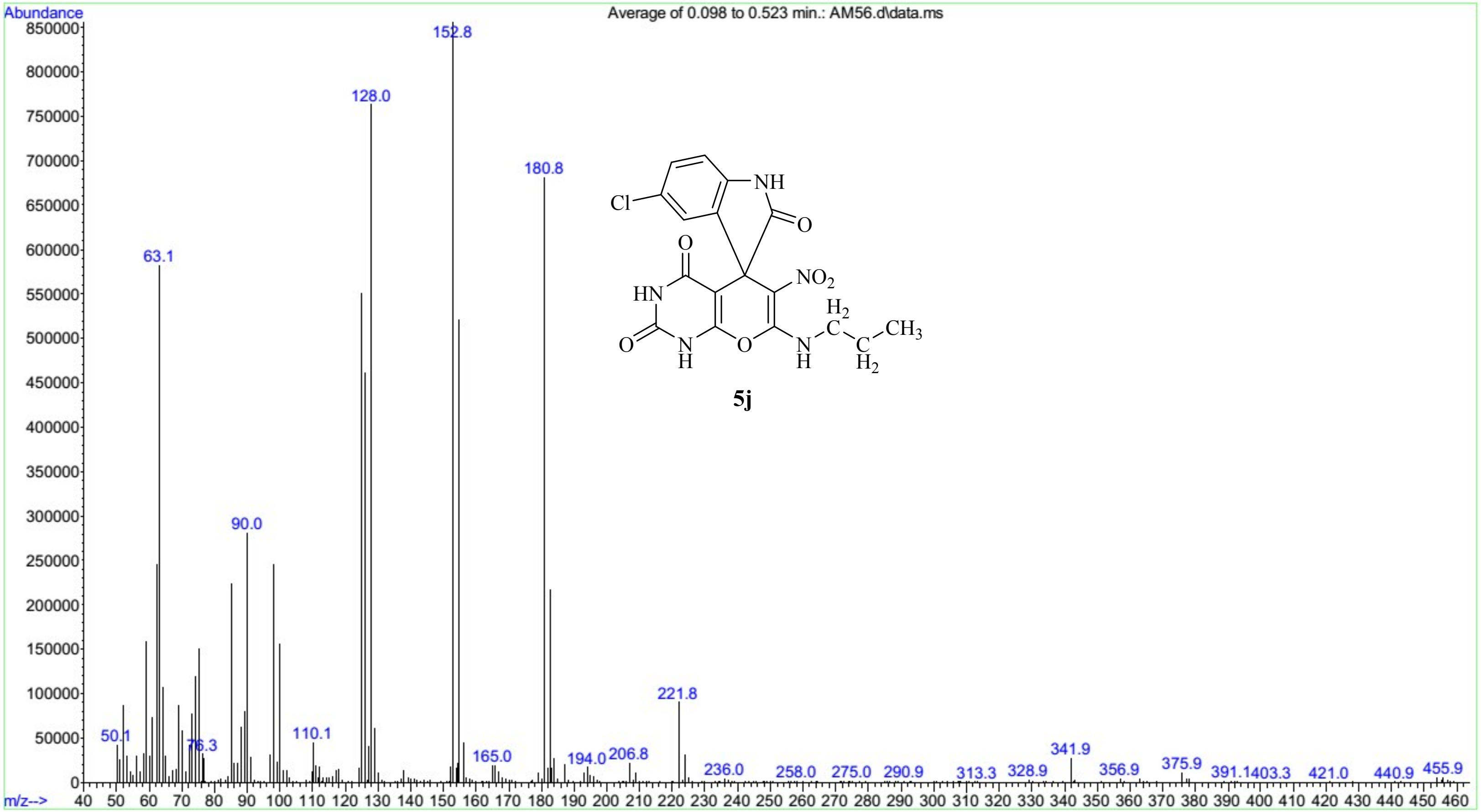
Abundance
Average of 0.098 to 0.523 min.: AM56.d\data.ms
m/z-->
152.8
128.0
180.8
63.1
90.0
221.8
110.1
50.1
76.3
165.0
194.0
206.8
236.0
258.0
275.0
290.9
313.3
328.9
341.9
356.9
375.9
391.1
403.3
421.0
440.9
455.9
Cl
NH
O
NO2
HN
H2
C
CH3
N
H
O
5j

**Mass of 5j**

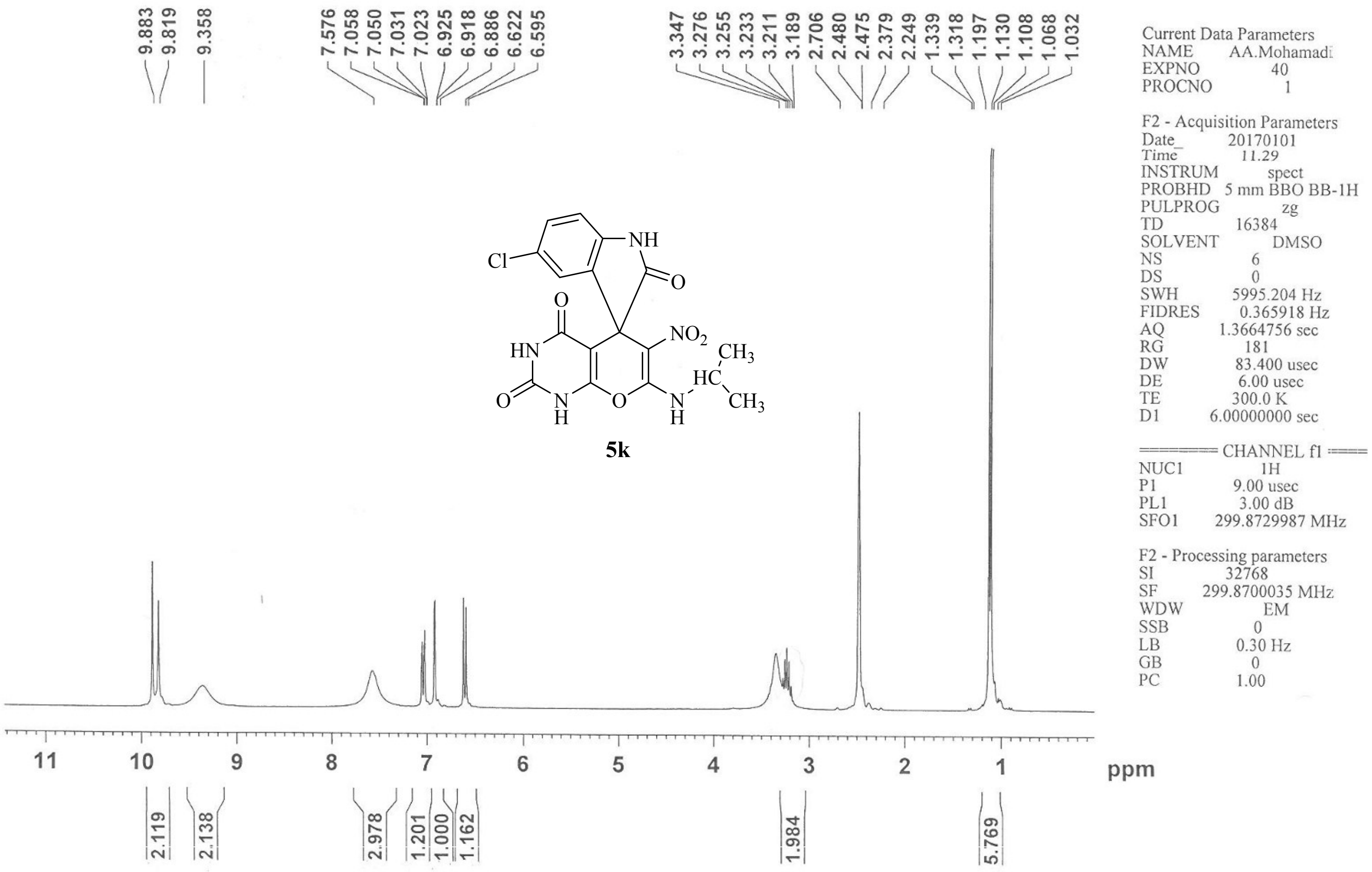
9.883
9.819
9.358
7.576
7.058
7.050
7.031
7.023
6.925
6.918
6.886
6.622
6.595
3.347
3.276
3.255
3.233
3.211
3.189
2.706
2.480
2.475
2.379
2.249
1.339
1.318
1.197
1.130
1.108
1.068
1.032
Cl
NH
O
O
NO2
HN
CH3
HC
O
N
H
O
N
H
CH3
5k
Current Data Parameters
NAME AA.Mohamadi
EXPNO 40
PROCNO 1
F2 - Acquisition Parameters
Date_ 20170101
Time 11.29
INSTRUM spect
PROBHD 5 mm BBO BB-1H
PULPROG zg
TD 16384
SOLVENT DMSO
NS 6
DS 0
SWH 5995.204 Hz
FIDRES 0.365918 Hz
AQ 1.3664756 sec
RG 181
DW 83.400 usec
DE 6.00 usec
TE 300.0 K
D1 6.00000000 sec
CHANNEL f1
NUC1 1H
P1 9.00 usec
PL1 3.00 dB
SFO1 299.8729987 MHz
F2 - Processing parameters
SI 32768
SF 299.8700035 MHz
WDW EM
SSB 0
LB 0.30 Hz
GB 0
PC 1.00
11
10
9
8
7
6
5
4
3
2
1
ppm
2.119
2.138
2.978
1.201
1.000
1.162
1.984
5.769

**$^1$H NMR of 5k**

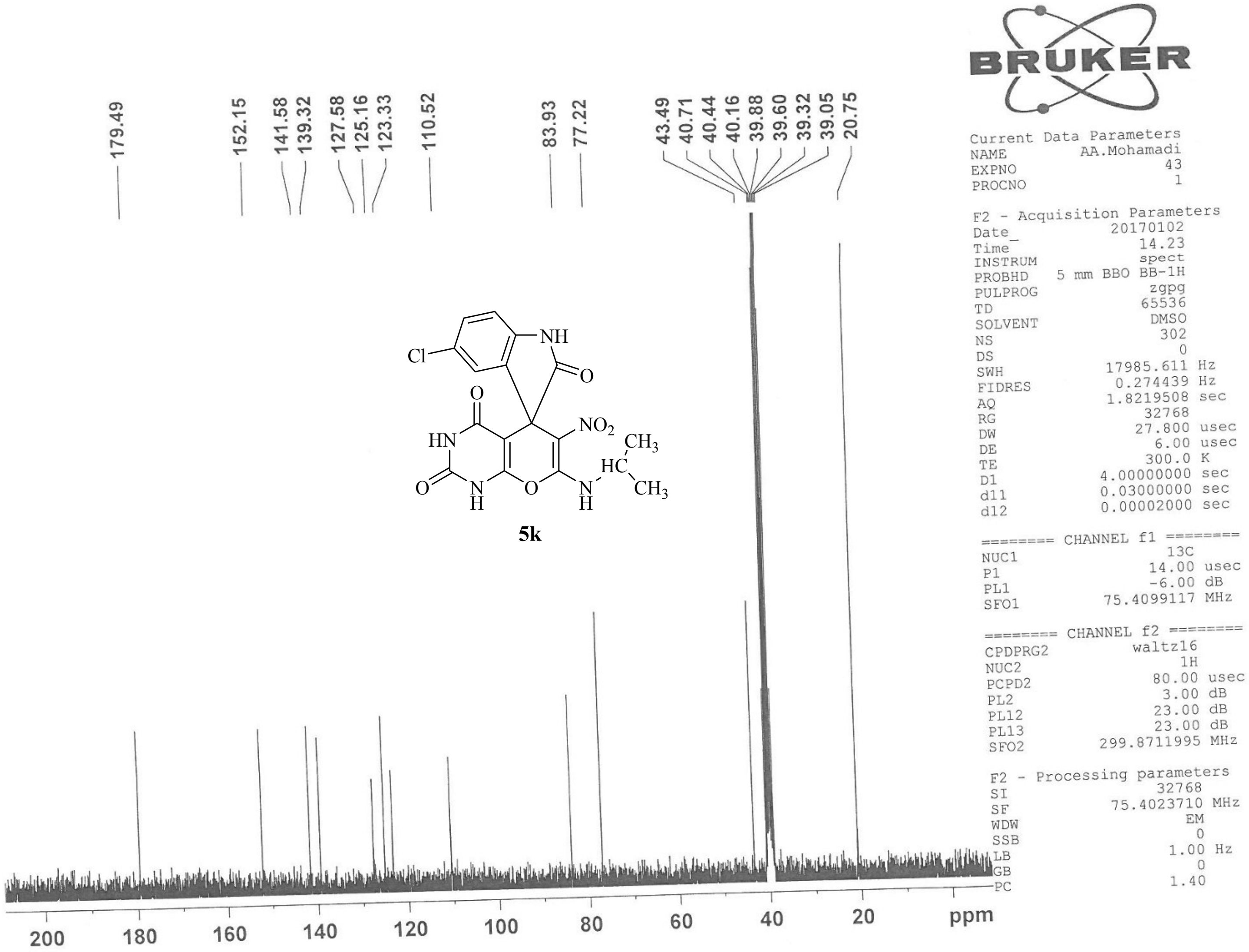
179.49
152.15
141.58
139.32
127.58
125.16
123.33
110.52
83.93
77.22
43.49
40.71
40.44
40.16
39.88
39.60
39.32
39.05
20.75
Cl
NH
O
O
NO2
HN
CH3
HC
O
N
H
O
N
H
CH3
5k
200
180
160
140
120
100
80
60
40
20
ppm
BRUKER
Current Data Parameters
NAME AA.Mohamadi
EXPNO 43
PROCNO 1
F2 - Acquisition Parameters
Date_ 20170102
Time 14.23
INSTRUM spect
PROBHD 5 mm BBO BB-1H
PULPROG zgpg
TD 65536
SOLVENT DMSO
NS 302
DS 0
SWH 17985.611 Hz
FIDRES 0.274439 Hz
AQ 1.8219508 sec
RG 32768
DW 27.800 usec
DE 6.00 usec
TE 300.0 K
D1 4.00000000 sec
d11 0.03000000 sec
d12 0.00002000 sec
======== CHANNEL f1 ========
NUC1 13C
P1 14.00 usec
PL1 -6.00 dB
SFO1 75.4099117 MHz
======== CHANNEL f2 ========
CPDPRG2 waltz16
NUC2 1H
PCPD2 80.00 usec
PL2 3.00 dB
PL12 23.00 dB
PL13 23.00 dB
SFO2 299.8711995 MHz
F2 - Processing parameters
SI 32768
SF 75.4023710 MHz
WDW EM
SSB 0
LB 1.00 Hz
GB 0
PC 1.40

**$^{13}$C NMR of 5k**

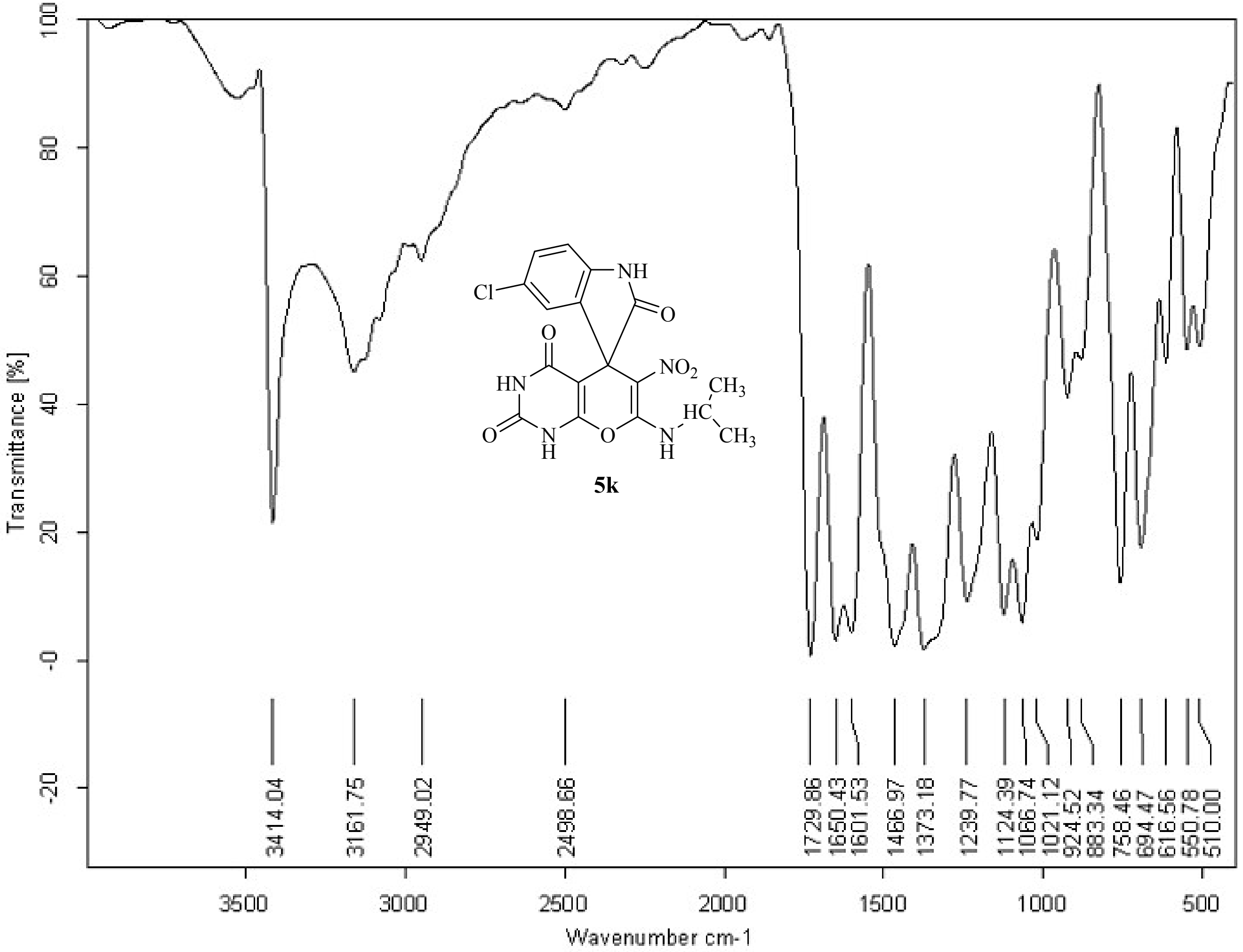

Transmittance [%]
Wavenumber cm-1
100
80
60
40
20
-0
-20
3500
3000
2500
2000
1500
1000
500
3414.04
3161.75
2949.02
2498.66
1729.86
1650.43
1601.53
1466.97
1373.18
1239.77
1124.39
1066.74
1021.12
924.52
883.34
758.46
694.47
616.56
550.78
510.00
Cl
NH
O
O
HN
NO2
CH3
HC
CH3
O
N
H
O
N
H
5k


**IR of 5k**

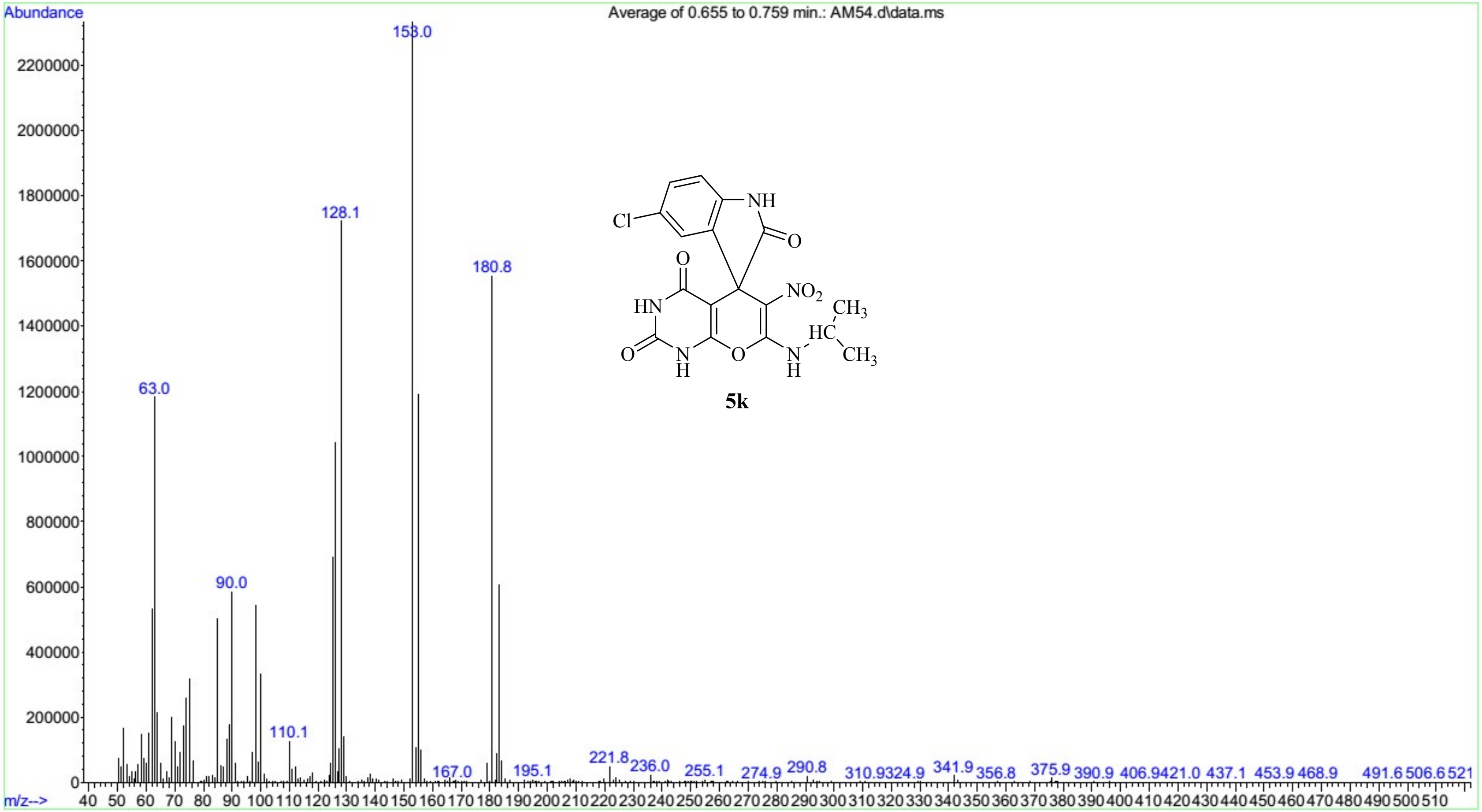

Abundance
Average of 0.655 to 0.759 min.: AM54.d\data.ms
153.0
128.1
180.8
63.0
90.0
110.1
167.0
195.1
221.8
236.0
255.1
274.9
290.8
310.9
324.9
341.9
356.8
375.9
390.9
406.9
421.0
437.1
453.9
468.9
491.6
506.6
521
m/z-->
Cl
NH
O
NO2
HN
CH3
HC
CH3
N
O
H
5k


**Mass of 5k**

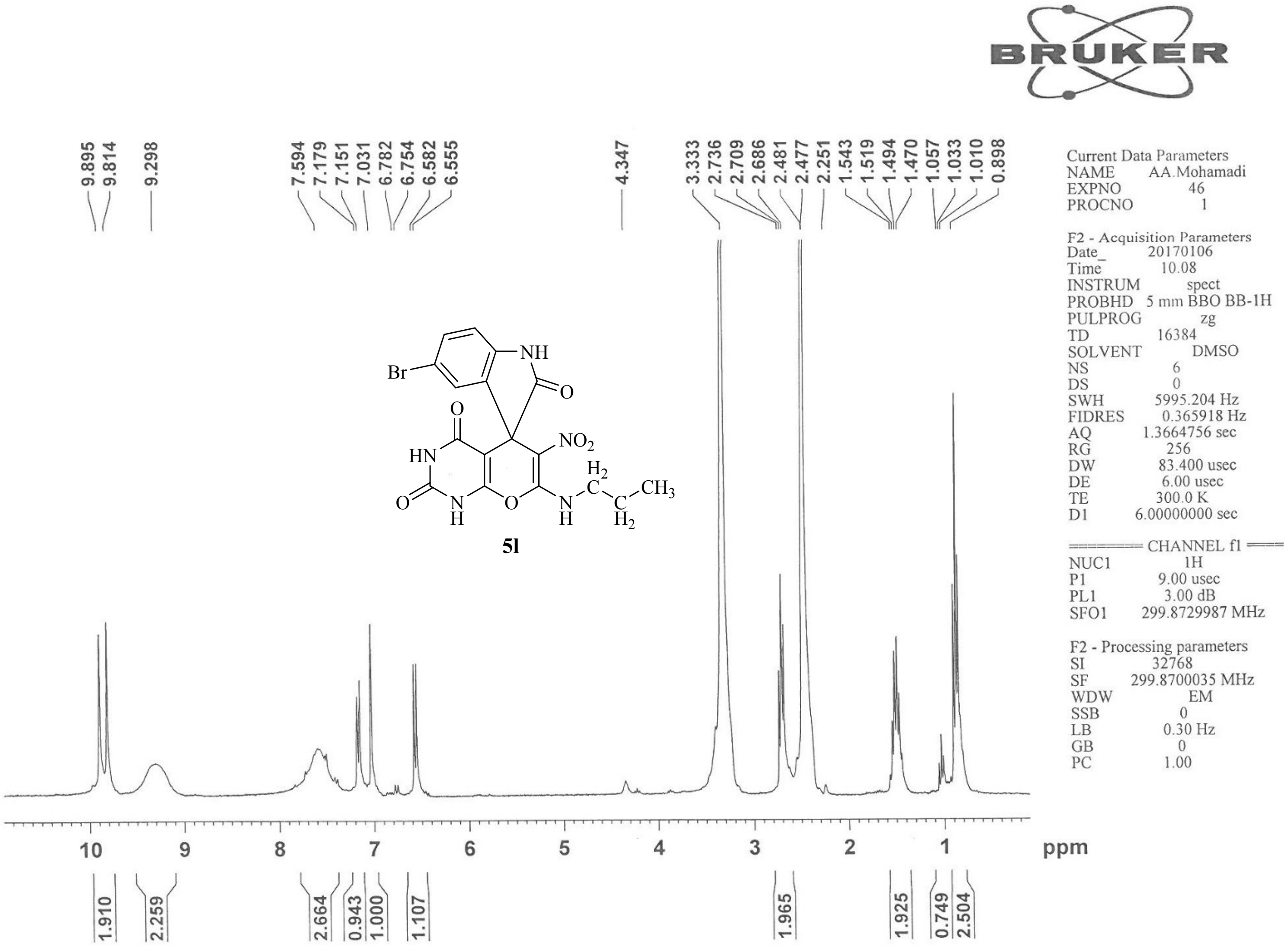

9.895
9.814
9.298
7.594
7.179
7.151
7.031
6.782
6.754
6.582
6.555
4.347
3.333
2.736
2.709
2.686
2.481
2.477
2.251
1.543
1.519
1.494
1.470
1.057
1.033
1.010
0.898
Current Data Parameters
NAME AA.Mohamadi
EXPNO 46
PROCNO 1
F2 - Acquisition Parameters
Date_ 20170106
Time 10.08
INSTRUM spect
PROBHD 5 mm BBO BB-1H
PULPROG zg
TD 16384
SOLVENT DMSO
NS 6
DS 0
SWH 5995.204 Hz
FIDRES 0.365918 Hz
AQ 1.3664756 sec
RG 256
DW 83.400 usec
DE 6.00 usec
TE 300.0 K
D1 6.00000000 sec
CHANNEL f1
NUC1 1H
P1 9.00 usec
PL1 3.00 dB
SFO1 299.8729987 MHz
F2 - Processing parameters
SI 32768
SF 299.8700035 MHz
WDW EM
SSB 0
LB 0.30 Hz
GB 0
PC 1.00
Br
NH
O
O
HN
NO2
O
N H
O
N H
H2 C
C H2
CH3
5l
10
9
8
7
6
5
4
3
2
1
ppm
1.910
2.259
2.664
0.943
1.000
1.107
1.965
1.925
0.749
2.504


**$^{1}$H NMR of 5l**

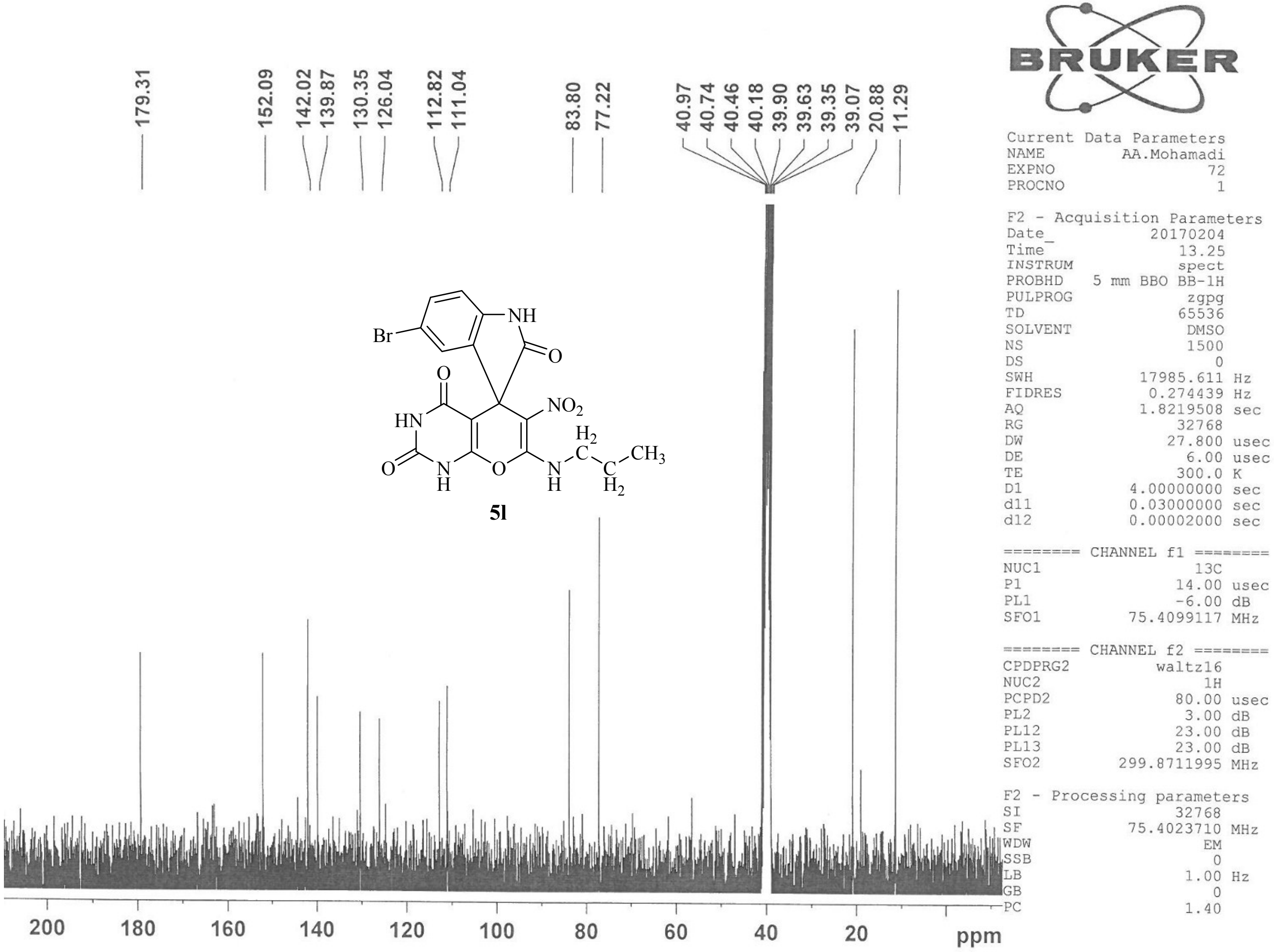
179.31
152.09
142.02
139.87
130.35
126.04
112.82
111.04
83.80
77.22
40.97
40.74
40.46
40.18
39.90
39.63
39.35
39.07
20.88
11.29
Br
NH
O
O
NO2
HN
H2
C
CH3
O
N
H
O
N
H
C
H2
5l
200
180
160
140
120
100
80
60
40
20
ppm
BRUKER
Current Data Parameters
NAME AA.Mohamadi
EXPNO 72
PROCNO 1
F2 - Acquisition Parameters
Date_ 20170204
Time 13.25
INSTRUM spect
PROBHD 5 mm BBO BB-1H
PULPROG zgpg
TD 65536
SOLVENT DMSO
NS 1500
DS 0
SWH 17985.611 Hz
FIDRES 0.274439 Hz
AQ 1.8219508 sec
RG 32768
DW 27.800 usec
DE 6.00 usec
TE 300.0 K
D1 4.00000000 sec
d11 0.03000000 sec
d12 0.00002000 sec
======== CHANNEL f1 ========
NUC1 13C
P1 14.00 usec
PL1 -6.00 dB
SFO1 75.4099117 MHz
======== CHANNEL f2 ========
CPDPRG2 waltz16
NUC2 1H
PCPD2 80.00 usec
PL2 3.00 dB
PL12 23.00 dB
PL13 23.00 dB
SFO2 299.8711995 MHz
F2 - Processing parameters
SI 32768
SF 75.4023710 MHz
WDW EM
SSB 0
LB 1.00 Hz
GB 0
PC 1.40

**$^{13}C$ NMR of 5l**

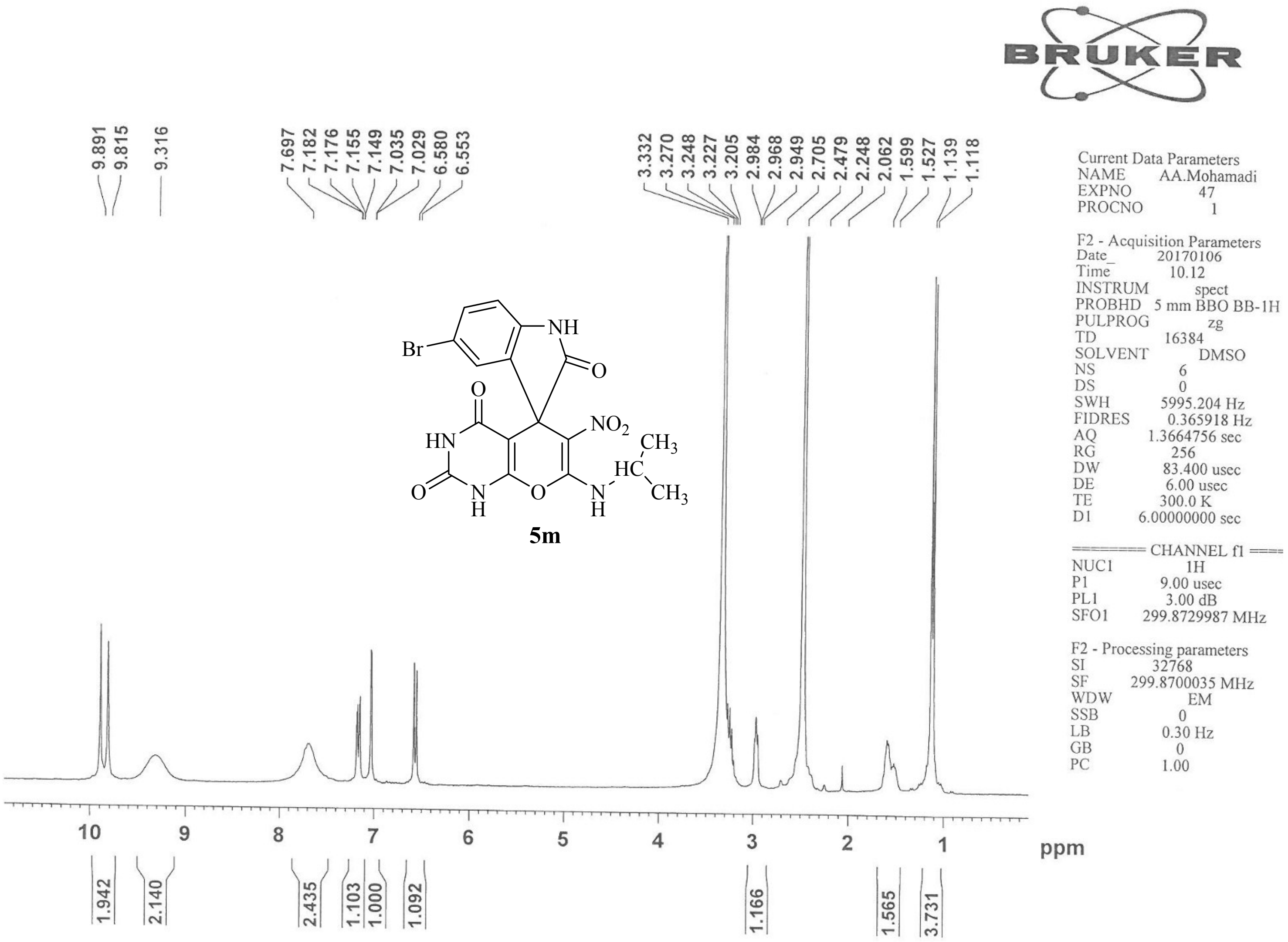
9.891
9.815
9.316
7.697
7.182
7.176
7.155
7.149
7.035
7.029
6.580
6.553
3.332
3.270
3.248
3.227
3.205
2.984
2.968
2.949
2.705
2.479
2.248
2.062
1.599
1.527
1.139
1.118
Br
NH
O
O
NO2
HN
CH3
HC
O
N
H
O
N
H
CH3
5m
Current Data Parameters
NAME AA.Mohamadi
EXPNO 47
PROCNO 1
F2 - Acquisition Parameters
Date_ 20170106
Time 10.12
INSTRUM spect
PROBHD 5 mm BBO BB-1H
PULPROG zg
TD 16384
SOLVENT DMSO
NS 6
DS 0
SWH 5995.204 Hz
FIDRES 0.365918 Hz
AQ 1.3664756 sec
RG 256
DW 83.400 usec
DE 6.00 usec
TE 300.0 K
D1 6.00000000 sec
CHANNEL f1
NUC1 1H
P1 9.00 usec
PL1 3.00 dB
SFO1 299.8729987 MHz
F2 - Processing parameters
SI 32768
SF 299.8700035 MHz
WDW EM
SSB 0
LB 0.30 Hz
GB 0
PC 1.00
10
9
8
7
6
5
4
3
2
1
ppm
1.942
2.140
2.435
1.103
1.000
1.092
1.166
1.565
3.731

**$^{1}$H NMR of 5m**

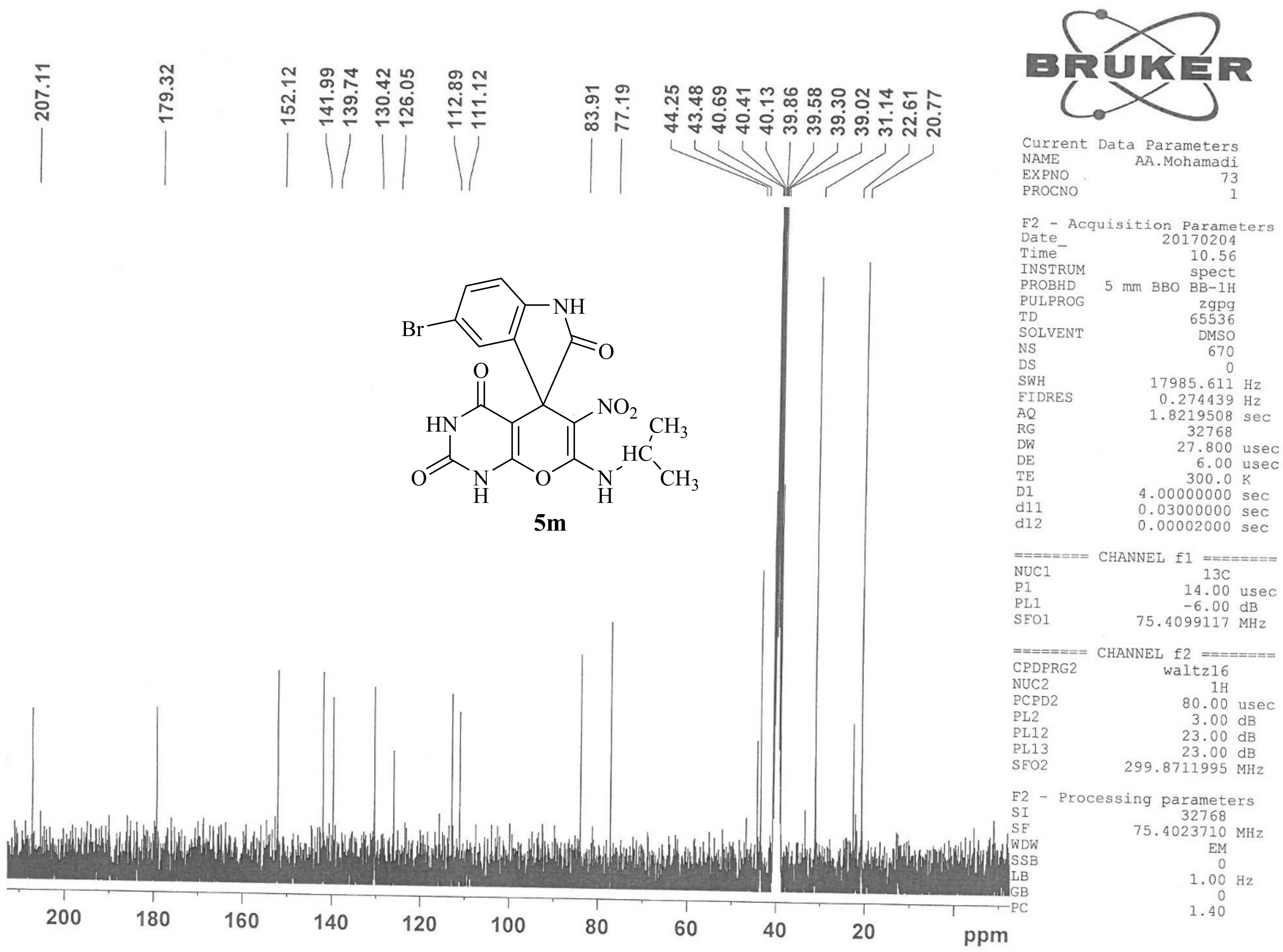
207.11
179.32
152.12
141.99
139.74
130.42
126.05
112.89
111.12
83.91
77.19
44.25
43.48
40.69
40.41
40.13
39.86
39.58
39.30
39.02
31.14
22.61
20.77
Br
NH
O
O
HN
NO2
CH3
HC
O
N
H
O
N
H
CH3
5m
200
180
160
140
120
100
80
60
40
20
ppm
BRUKER
Current Data Parameters
NAME AA.Mohamadi
EXPNO 73
PROCNO 1
F2 - Acquisition Parameters
Date_ 20170204
Time 10.56
INSTRUM spect
PROBHD 5 mm BBO BB-1H
PULPROG zgpg
TD 65536
SOLVENT DMSO
NS 670
DS 0
SWH 17985.611 Hz
FIDRES 0.274439 Hz
AQ 1.8219508 sec
RG 32768
DW 27.800 usec
DE 6.00 usec
TE 300.0 K
D1 4.00000000 sec
d11 0.03000000 sec
d12 0.00002000 sec
======== CHANNEL f1 ========
NUC1 13C
P1 14.00 usec
PL1 -6.00 dB
SFO1 75.4099117 MHz
======== CHANNEL f2 ========
CPDPRG2 waltz16
NUC2 1H
PCPD2 80.00 usec
PL2 3.00 dB
PL12 23.00 dB
PL13 23.00 dB
SFO2 299.8711995 MHz
F2 - Processing parameters
SI 32768
SF 75.4023710 MHz
WDW EM
SSB 0
LB 1.00 Hz
GB 0
PC 1.40

**$^{13}C$ NMR of 5m**

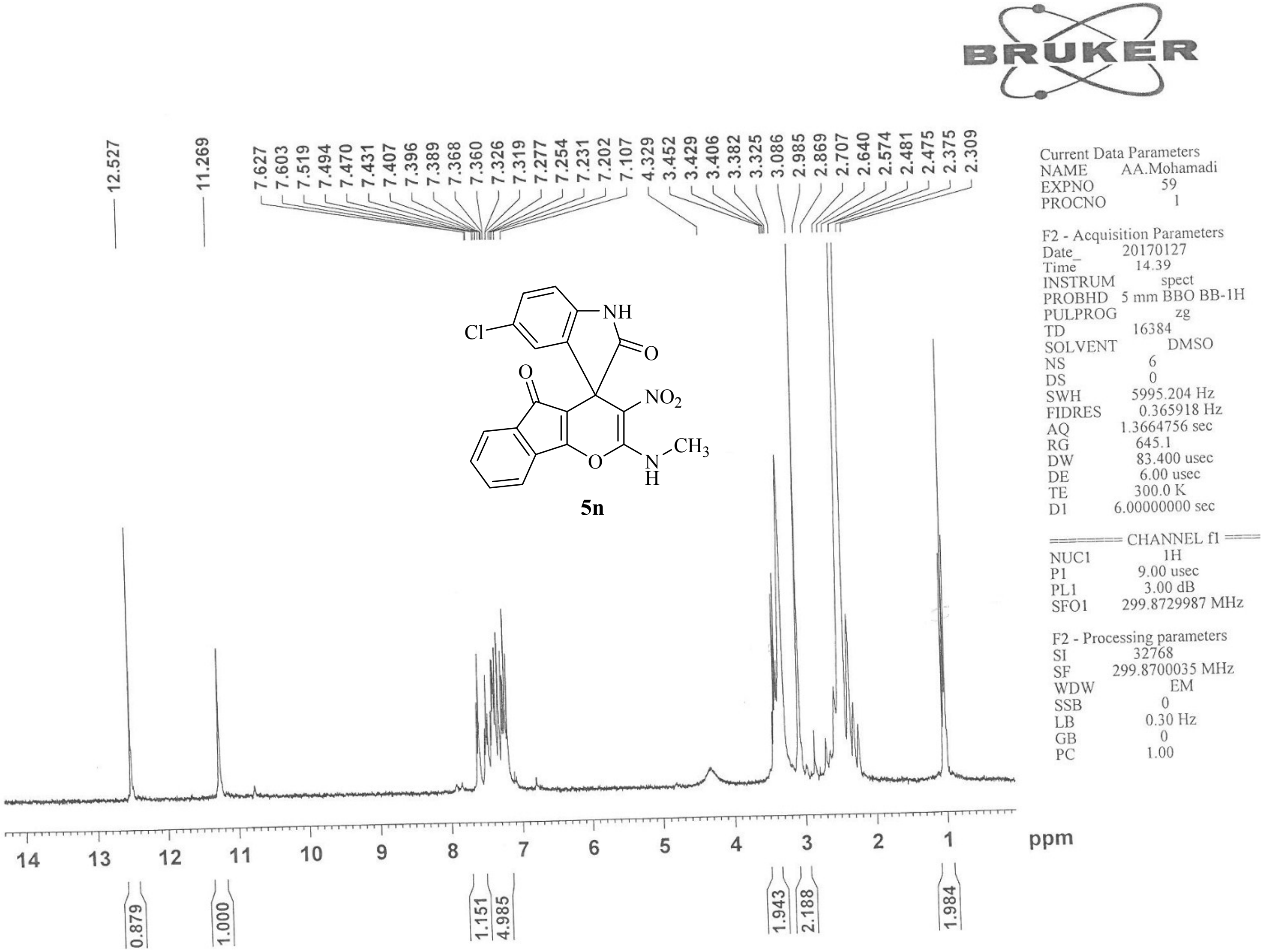
Current Data Parameters
NAME AA.Mohamadi
EXPNO 59
PROCNO 1
F2 - Acquisition Parameters
Date_ 20170127
Time 14.39
INSTRUM spect
PROBHD 5 mm BBO BB-1H
PULPROG zg
TD 16384
SOLVENT DMSO
NS 6
DS 0
SWH 5995.204 Hz
FIDRES 0.365918 Hz
AQ 1.3664756 sec
RG 645.1
DW 83.400 usec
DE 6.00 usec
TE 300.0 K
D1 6.00000000 sec
CHANNEL f1
NUC1 1H
P1 9.00 usec
PL1 3.00 dB
SFO1 299.8729987 MHz
F2 - Processing parameters
SI 32768
SF 299.8700035 MHz
WDW EM
SSB 0
LB 0.30 Hz
GB 0
PC 1.00
ppm
5n

**$^{1}$H NMR of 5n**

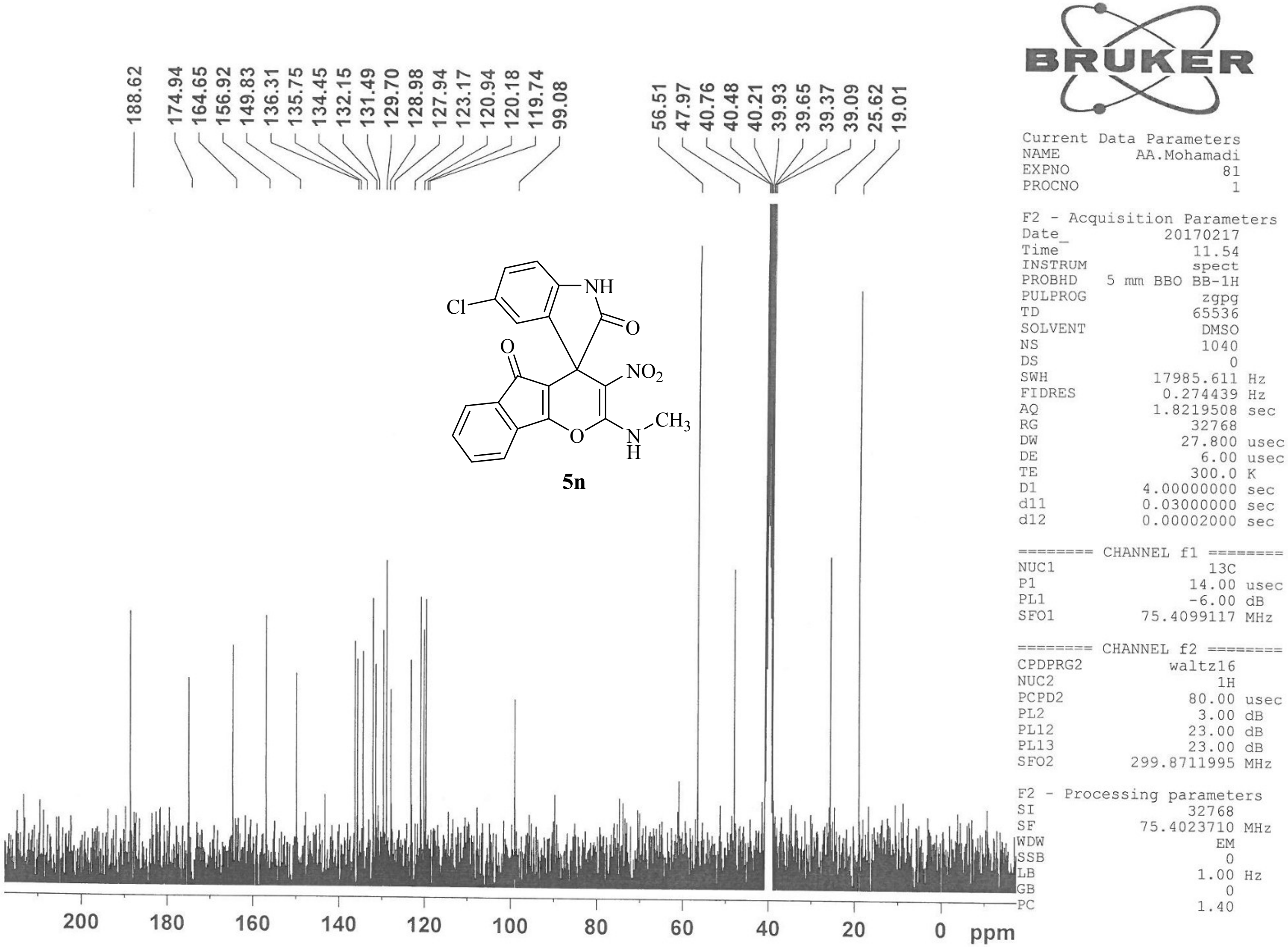
188.62
174.94
164.65
156.92
149.83
136.31
135.75
134.45
132.15
131.49
129.70
128.98
127.94
123.17
120.94
120.18
119.74
99.08
56.51
47.97
40.76
40.48
40.21
39.93
39.65
39.37
39.09
25.62
19.01
BRUKER
Current Data Parameters
NAME AA.Mohamadi
EXPNO 81
PROCNO 1
F2 - Acquisition Parameters
Date_ 20170217
Time 11.54
INSTRUM spect
PROBHD 5 mm BBO BB-1H
PULPROG zgpg
TD 65536
SOLVENT DMSO
NS 1040
DS 0
SWH 17985.611 Hz
FIDRES 0.274439 Hz
AQ 1.8219508 sec
RG 32768
DW 27.800 usec
DE 6.00 usec
TE 300.0 K
D1 4.00000000 sec
d11 0.03000000 sec
d12 0.00002000 sec
======== CHANNEL f1 ========
NUC1 13C
P1 14.00 usec
PL1 -6.00 dB
SFO1 75.4099117 MHz
======== CHANNEL f2 ========
CPDPRG2 waltz16
NUC2 1H
PCPD2 80.00 usec
PL2 3.00 dB
PL12 23.00 dB
PL13 23.00 dB
SFO2 299.8711995 MHz
F2 - Processing parameters
SI 32768
SF 75.4023710 MHz
WDW EM
SSB 0
LB 1.00 Hz
GB 0
PC 1.40
Cl
NH
O
O
NO2
O
N
CH3
H
5n
200 180 160 140 120 100 80 60 40 20 0 ppm

**$^{13}$C NMR of 5n**

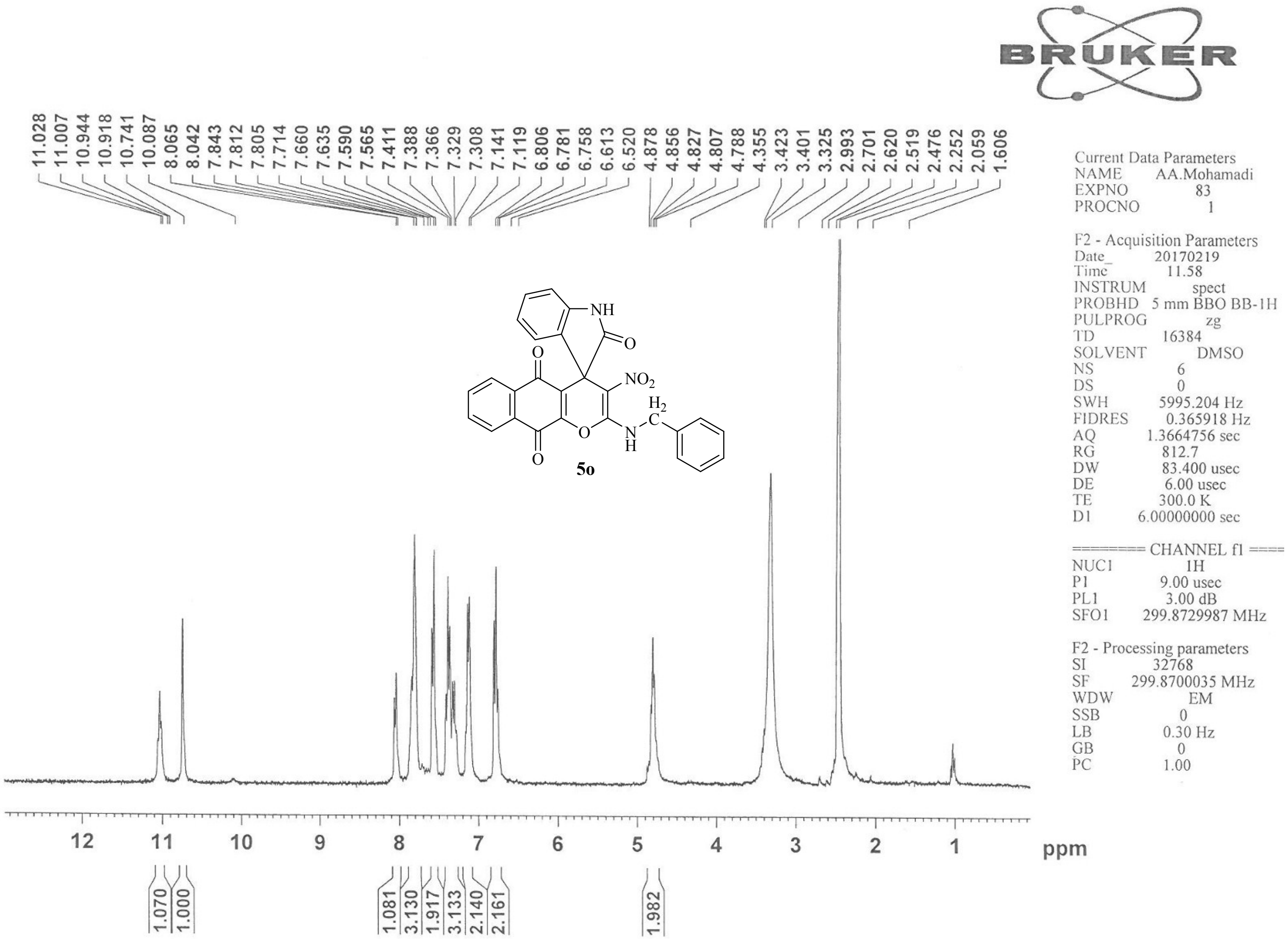
11.028
11.007
10.944
10.918
10.741
10.087
8.065
8.042
7.843
7.812
7.805
7.714
7.660
7.635
7.590
7.565
7.411
7.388
7.366
7.329
7.308
7.141
7.119
6.806
6.781
6.758
6.613
6.520
4.878
4.856
4.827
4.807
4.788
4.355
3.423
3.401
3.325
2.993
2.701
2.620
2.519
2.476
2.252
2.059
1.606
NH
O
O
$NO_2$
$H_2$
C
N
H
O
O
5o
12 11 10 9 8 7 6 5 4 3 2 1 ppm
1.070
1.000
1.081
3.130
1.917
3.133
2.140
2.161
1.982
BRUKER
Current Data Parameters
NAME AA.Mohamadi
EXPNO 83
PROCNO 1
F2 - Acquisition Parameters
Date_ 20170219
Time 11.58
INSTRUM spect
PROBHD 5 mm BBO BB-1H
PULPROG zg
TD 16384
SOLVENT DMSO
NS 6
DS 0
SWH 5995.204 Hz
FIDRES 0.365918 Hz
AQ 1.3664756 sec
RG 812.7
DW 83.400 usec
DE 6.00 usec
TE 300.0 K
D1 6.00000000 sec
======== CHANNEL f1 ====
NUC1 1H
P1 9.00 usec
PL1 3.00 dB
SFO1 299.8729987 MHz
F2 - Processing parameters
SI 32768
SF 299.8700035 MHz
WDW EM
SSB 0
LB 0.30 Hz
GB 0
PC 1.00

**$^{1}H$ NMR of 5o**

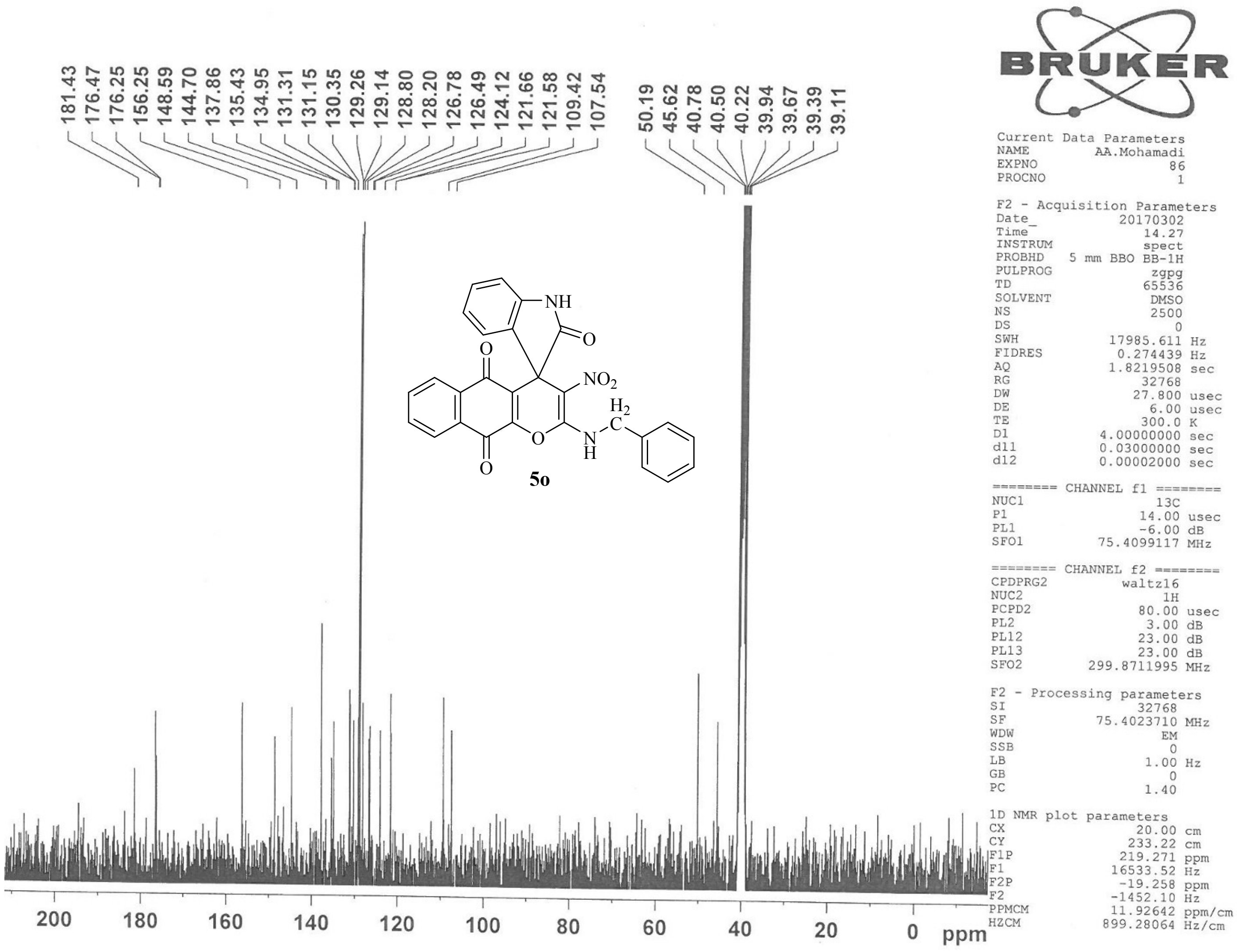
181.43
176.47
176.25
156.25
148.59
144.70
137.86
135.43
134.95
131.31
131.15
130.35
129.26
129.14
128.80
128.20
126.78
126.49
124.12
121.66
121.58
109.42
107.54
50.19
45.62
40.78
40.50
40.22
39.94
39.67
39.39
39.11
NH
O
O
$NO_2$
$H_2$
C
N
H
O
O
5o
BRUKER
Current Data Parameters
NAME AA.Mohamadi
EXPNO 86
PROCNO 1
F2 - Acquisition Parameters
Date_ 20170302
Time 14.27
INSTRUM spect
PROBHD 5 mm BBO BB-1H
PULPROG zgpg
TD 65536
SOLVENT DMSO
NS 2500
DS 0
SWH 17985.611 Hz
FIDRES 0.274439 Hz
AQ 1.8219508 sec
RG 32768
DW 27.800 usec
DE 6.00 usec
TE 300.0 K
D1 4.00000000 sec
d11 0.03000000 sec
d12 0.00002000 sec
======== CHANNEL f1 ========
NUC1 13C
P1 14.00 usec
PL1 -6.00 dB
SFO1 75.4099117 MHz
======== CHANNEL f2 ========
CPDPRG2 waltz16
NUC2 1H
PCPD2 80.00 usec
PL2 3.00 dB
PL12 23.00 dB
PL13 23.00 dB
SFO2 299.8711995 MHz
F2 - Processing parameters
SI 32768
SF 75.4023710 MHz
WDW EM
SSB 0
LB 1.00 Hz
GB 0
PC 1.40
1D NMR plot parameters
CX 20.00 cm
CY 233.22 cm
F1P 219.271 ppm
F1 16533.52 Hz
F2P -19.258 ppm
F2 -1452.10 Hz
PPMCM 11.92642 ppm/cm
HZCM 899.28064 Hz/cm
200
180
160
140
120
100
80
60
40
20
0
ppm

**$^{13}$C NMR of 5o**

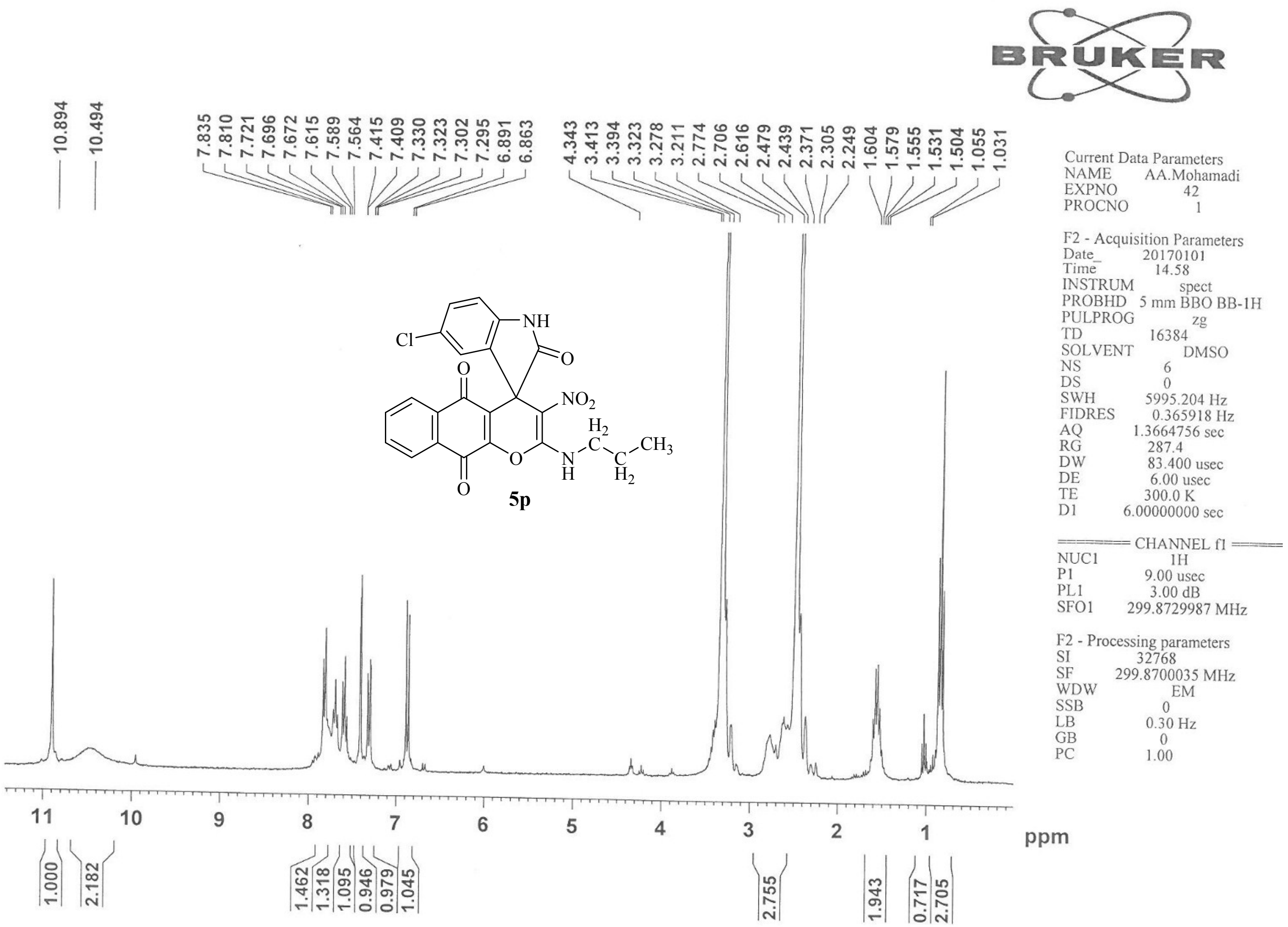
BRUKER
10.894
10.494
7.835
7.810
7.721
7.696
7.672
7.615
7.589
7.564
7.415
7.409
7.330
7.323
7.302
7.295
6.891
6.863
4.343
3.413
3.394
3.323
3.278
3.211
2.774
2.706
2.616
2.479
2.439
2.371
2.305
2.249
1.604
1.579
1.555
1.531
1.504
1.055
1.031
Current Data Parameters
NAME AA.Mohamadi
EXPNO 42
PROCNO 1
F2 - Acquisition Parameters
Date_ 20170101
Time 14.58
INSTRUM spect
PROBHD 5 mm BBO BB-1H
PULPROG zg
TD 16384
SOLVENT DMSO
NS 6
DS 0
SWH 5995.204 Hz
FIDRES 0.365918 Hz
AQ 1.3664756 sec
RG 287.4
DW 83.400 usec
DE 6.00 usec
TE 300.0 K
D1 6.00000000 sec
CHANNEL f1
NUC1 1H
P1 9.00 usec
PL1 3.00 dB
SFO1 299.8729987 MHz
F2 - Processing parameters
SI 32768
SF 299.8700035 MHz
WDW EM
SSB 0
LB 0.30 Hz
GB 0
PC 1.00
Cl
NH
O
O
NO2
H2
C
CH3
N
H
C
H2
O
O
5p
11
10
9
8
7
6
5
4
3
2
1
ppm
1.000
2.182
1.462
1.318
1.095
0.946
0.979
1.045
2.755
1.943
0.717
2.705

**$^{1}$H NMR of 5p**

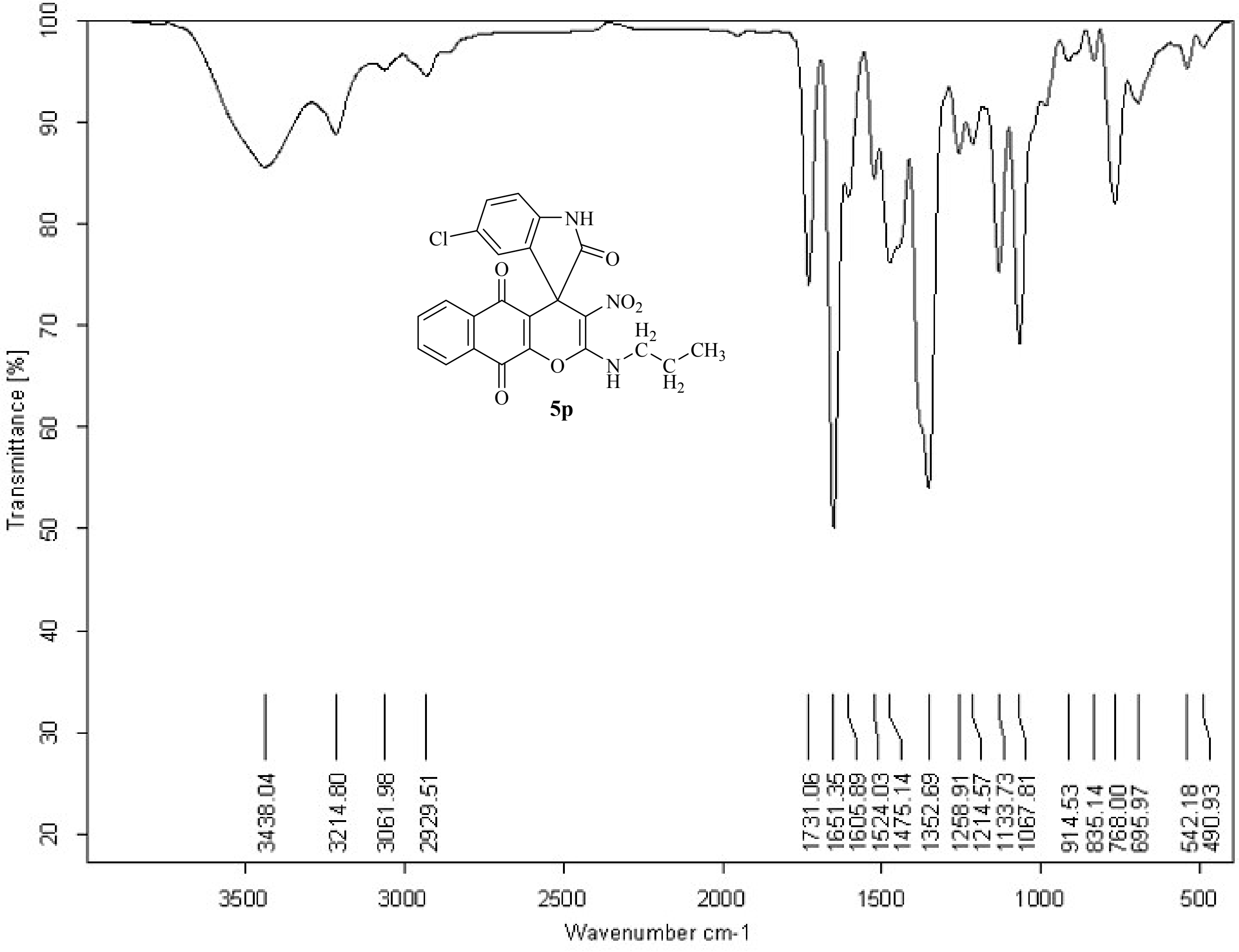
100
90
80
70
60
50
40
30
20
Transmittance [%]
3438.04
3214.80
3061.98
2929.51
1731.06
1651.35
1605.89
1524.03
1475.14
1352.69
1258.91
1214.57
1133.73
1067.81
914.53
835.14
768.00
695.97
542.18
490.93
3500
3000
2500
2000
1500
1000
500
Wavenumber cm-1
Cl
NH
O
O
NO2
O
O
N
H
H2
C
C
H2
CH3
5p

**IR of 5p**

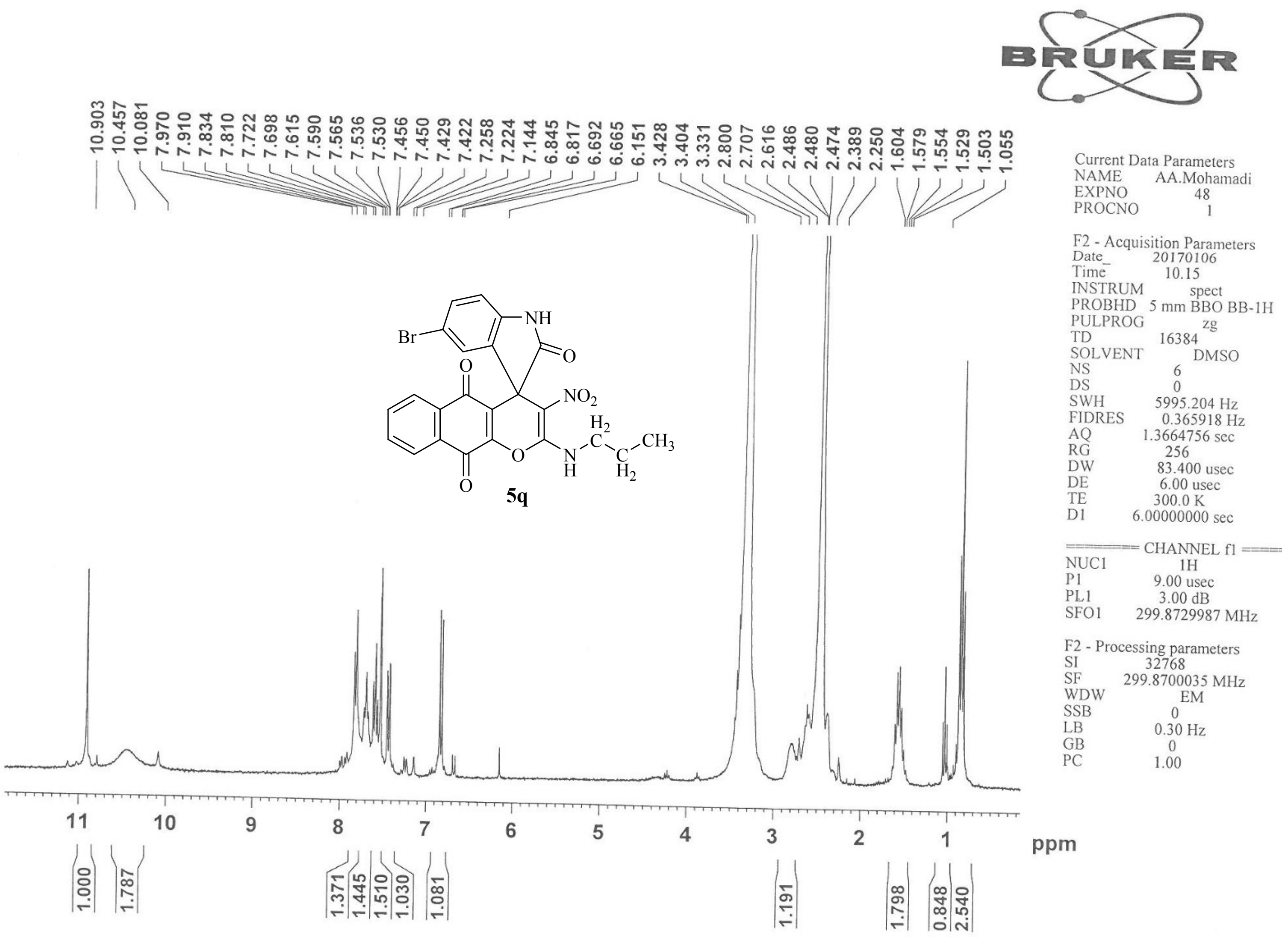
BRUKER
Current Data Parameters
NAME AA.Mohamadi
EXPNO 48
PROCNO 1
F2 - Acquisition Parameters
Date_ 20170106
Time 10.15
INSTRUM spect
PROBHD 5 mm BBO BB-1H
PULPROG zg
TD 16384
SOLVENT DMSO
NS 6
DS 0
SWH 5995.204 Hz
FIDRES 0.365918 Hz
AQ 1.3664756 sec
RG 256
DW 83.400 usec
DE 6.00 usec
TE 300.0 K
D1 6.00000000 sec
CHANNEL f1
NUC1 1H
P1 9.00 usec
PL1 3.00 dB
SFO1 299.8729987 MHz
F2 - Processing parameters
SI 32768
SF 299.8700035 MHz
WDW EM
SSB 0
LB 0.30 Hz
GB 0
PC 1.00
5q
ppm

**$^1$H NMR of 5q**

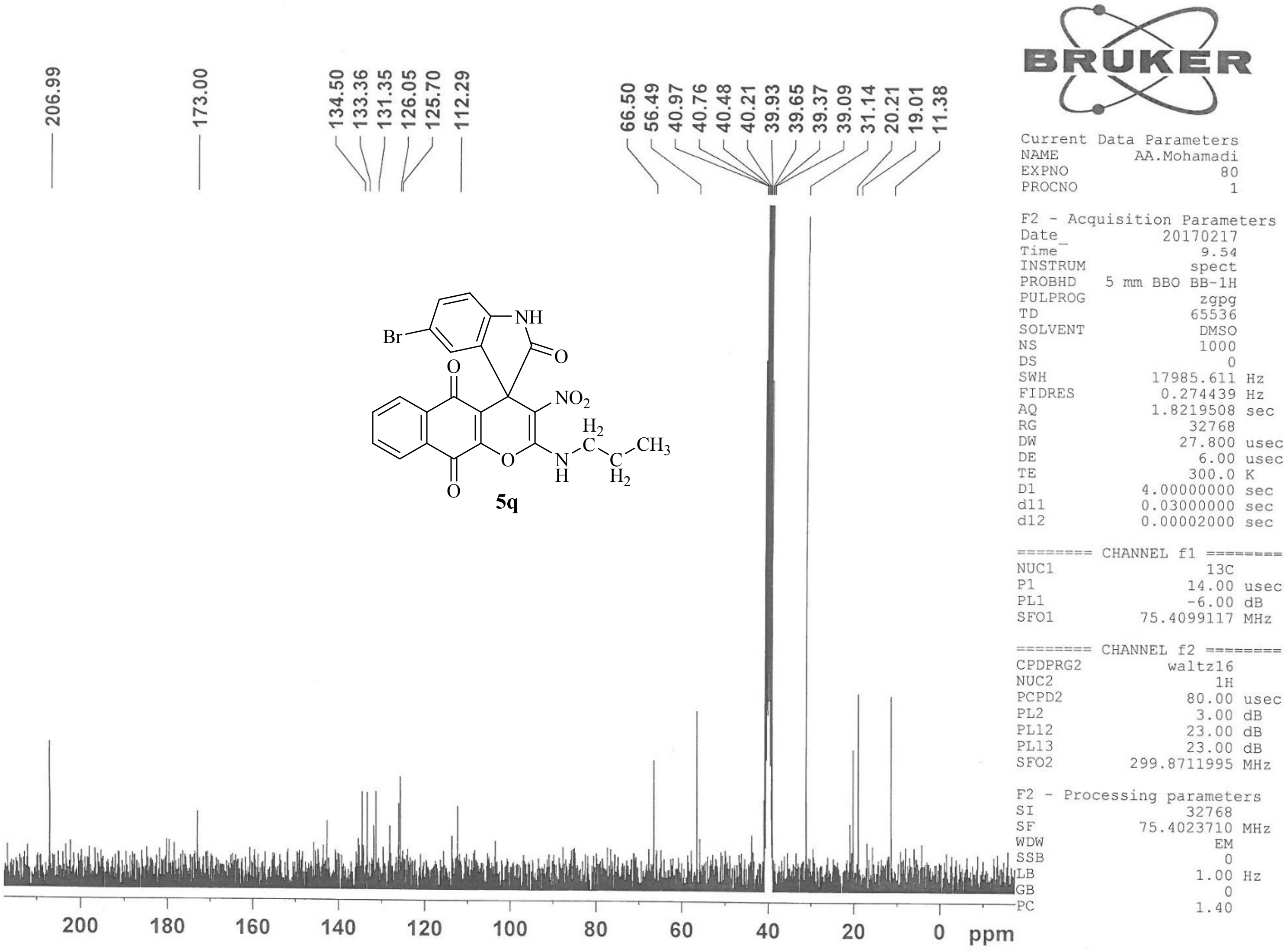
206.99
173.00
134.50
133.36
131.35
126.05
125.70
112.29
66.50
56.49
40.97
40.76
40.48
40.21
39.93
39.65
39.37
39.09
31.14
20.21
19.01
11.38
BRUKER
Current Data Parameters
NAME AA.Mohamadi
EXPNO 80
PROCNO 1
F2 - Acquisition Parameters
Date_ 20170217
Time 9.54
INSTRUM spect
PROBHD 5 mm BBO BB-1H
PULPROG zgpg
TD 65536
SOLVENT DMSO
NS 1000
DS 0
SWH 17985.611 Hz
FIDRES 0.274439 Hz
AQ 1.8219508 sec
RG 32768
DW 27.800 usec
DE 6.00 usec
TE 300.0 K
D1 4.00000000 sec
d11 0.03000000 sec
d12 0.00002000 sec
======== CHANNEL f1 ========
NUC1 13C
P1 14.00 usec
PL1 -6.00 dB
SFO1 75.4099117 MHz
======== CHANNEL f2 ========
CPDPRG2 waltz16
NUC2 1H
PCPD2 80.00 usec
PL2 3.00 dB
PL12 23.00 dB
PL13 23.00 dB
SFO2 299.8711995 MHz
F2 - Processing parameters
SI 32768
SF 75.4023710 MHz
WDW EM
SSB 0
LB 1.00 Hz
GB 0
PC 1.40
Br
NH
O
O
NO2
H2
C
CH3
N
H
C
H2
O
O
5q
200
180
160
140
120
100
80
60
40
20
0
ppm

**$^{13}$C NMR of 5q**
